# Microarray Analyses of Gene Expression during the *Tetrahymena thermophila* Life Cycle

**DOI:** 10.1371/journal.pone.0004429

**Published:** 2009-02-10

**Authors:** Wei Miao, Jie Xiong, Josephine Bowen, Wei Wang, Yifan Liu, Olga Braguinets, Jorg Grigull, Ronald E. Pearlman, Eduardo Orias, Martin A. Gorovsky

**Affiliations:** 1 Department of Biology, University of Rochester, Rochester, New York, United States of America; 2 State Key Laboratory of Freshwater Ecology and Biotechnology, Institute of Hydrobiology, Chinese Academy of Sciences, Wuhan, China; 3 Institute of Biotechnology, Shanxi University, Taiyuan, China; 4 Pathology Department, University of Michigan Medical School, Ann Arbor, Michigan, United States of America; 5 Department of Biology, York University, Toronto, Ontario, Canada; 6 Department of Mathematics and Statistics, York University, Toronto, Ontario, Canada; 7 Department of Molecular, Cellular, and Developmental Biology, University of California Santa Barbara, Santa Barbara, California, United States of America; Universidade de Sao Paulo, Brazil

## Abstract

**Background:**

The model eukaryote, *Tetrahymena thermophila,* is the first ciliated protozoan whose genome has been sequenced, enabling genome-wide analysis of gene expression.

**Methodology/Principal Findings:**

A genome-wide microarray platform containing the predicted coding sequences (putative genes) for *T. thermophila* is described, validated and used to study gene expression during the three major stages of the organism's life cycle: growth, starvation and conjugation.

**Conclusions/Significance:**

Of the ∼27,000 predicted open reading frames, transcripts homologous to only ∼5900 are not detectable in any of these life cycle stages, indicating that this single-celled organism does indeed contain a large number of functional genes. Transcripts from over 5000 predicted genes are expressed at levels >5× corrected background and 95 genes are expressed at >250× corrected background in all stages. Transcripts homologous to 91 predicted genes are specifically expressed and 155 more are highly up-regulated in growing cells, while 90 are specifically expressed and 616 are up-regulated during starvation. Strikingly, transcripts homologous to 1068 predicted genes are specifically expressed and 1753 are significantly up-regulated during conjugation. The patterns of gene expression during conjugation correlate well with the developmental stages of meiosis, nuclear differentiation and DNA elimination. The relationship between gene expression and chromosome fragmentation is analyzed. Genes encoding proteins known to interact or to function in complexes show similar expression patterns, indicating that co-ordinate expression with putative genes of known function can identify genes with related functions. New candidate genes associated with the RNAi-like process of DNA elimination and with meiosis are identified and the late stages of conjugation are shown to be characterized by specific expression of an unexpectedly large and diverse number of genes not involved in nuclear functions.

## Introduction

*Tetrahymena* is a genus of free-living ciliated protozoans that is widely distributed in freshwater environments around the world. Ciliates are evolutionarily grouped with the exclusively parasitic Apicomplexa and with the Dinoflagellates to form the Alveolates, indicating that studying them is likely to illuminate novel properties of these organisms with significant medical and ecological impact.

*Tetrahymena thermophila* is well-established as a model eukaryote, elaborating typical eukaryotic components (eg, microtubules, membrane systems) into a highly organized cell whose structural and functional complexity is comparable to, or exceeds that, of human and other metazoan cells [1]. Importantly, *Tetrahymena*'s special elaborations of certain basic eukaryotic mechanisms have facilitated discoveries opening major new fields of fundamental research over the last five decades, including the discovery of dynein, the first microtubule motor [2], elucidation of the fundamental structure of telomeres [3], the discovery of catalytic RNA [4], the discovery and characterization of telomerase [5], the first demonstration that a transcription factor (GCN5p) acts by catalyzing a histone post-translational modification [6] and the co-discovery that an RNAi-like process acts to target changes in chromatin function [7].

Perhaps the most salient feature of *Tetrahymena* is its nuclear dimorphism, whose study has provided the basis for many of the major advances in *Tetrahymena* genetics and for many discoveries in this organism [8]–[11]. Each cell has two nuclei that contain distinct but closely related genomes. The micronucleus (MIC) is the germline. Like the nuclei of germline cells in multicellular organisms, it is the storehouse of genetic information that is passed on to sexual progeny. The MIC is diploid (2C), contains 5 pairs of metacentric chromosomes and divides mitotically. No RNA synthesis or RNA containing structures (e.g., nucleolus, heterogeneous nuclear RNPs) can be observed and no genes are detectably expressed in the MIC during vegetative proliferation. The macronucleus (MAC) is the somatic nucleus. Like the nuclei of somatic cells in multicellular organisms, it is actively transcribed during vegetative proliferation and determines the cell's phenotype. The MAC is composed of ∼225 chromosomes that behave as if they are acentric. It is polyploid (∼45C) and divides amitotically, randomly distributing chromosome copies to the sister cells produced during vegetative growth.

MACs and MICs contain largely the same DNA sequences (see below) and have a common origin during conjugation, the sexual stage of the life cycle. Conjugation can be induced with a high degree of synchrony when cells of any 2 of the 7 different mating types are starved and mixed together. Thus, starvation is not only a distinct physiological state that *Tetrahymena* likely encounters in its freshwater environment, but it also induces numerous phenotypic and behavioral changes resulting in the acquisition of competence for mating. Cells that are not starved do not mate and mixing starved cells of different mating types initiates a series of developmental events that lead to mating, and that are independent of the mating types of the cells involved [12]–[14]. The nuclear events that occur in conjugating *Tetrahymena* have clear parallels in multicellular eukaryotes and include meiosis, formation of pronuclei, pronuclear fusion, postzygotic divisions, and cytoplasmic determination of nuclear fate. The large size of *Tetrahymena* cells and the distinct cytology of the nuclear events enable staging of the conjugation process [15]–[17]. The ability to perform highly synchronous large-scale matings and the ease with which *Tetrahymena* can be analyzed cytologically, biochemically and genetically make it highly attractive for studying the expression and regulation of genes during conjugation.

The most intensely studied events that occur during conjugation involve genome-wide DNA rearrangement. During development of a new MAC from the mitotic division products of the zygotic MIC, the MAC genome undergoes a remarkable series of programmed epigenetic changes and genome rearrangements, becoming streamlined for efficient replication and transcription. During rapid vegetative growth, *Tetrahymena* cells duplicate an amount of DNA similar to that of a mammalian cell and double their much larger cytoplasmic volume in an interval approximately ten times shorter than a mammalian cell generation time. Genome rearrangements include chromosome fragmentation, elimination of centromeres, selfish genetic elements and other repetitive DNA, and ribosomal gene amplification, followed by endoreplication of the gene-enriched MAC genome (for review, see [11]). A precise process of chromosome fragmentation creates ∼225 MAC chromosomes from the 5 MIC chromosomes. Chromosome breakage is accompanied by loss of a small amount (<75 bp) of DNA termed “breakage eliminated sequences” (BESs), coordinated with new telomere addition to form stable MAC chromosome ends. The breakage sites are determined by a relatively conserved 15 bp “chromosome breakage sequence” (CBS) with a completely conserved 10 bp core that is both necessary and sufficient for telomere formation [18], [19]. The factors that recognize the CBS sequence motif, catalyze endonucleolytic cleavage and specify new telomere synthesis at non-telomeric sites are unknown. Telomere formation in *Tetrahymena* offers a special opportunity to identify genes involved in telomerase-mediated chromosome healing, a process with clear medical relevance to human disease [20].

A second type of programmed genome rearrangement results in less precise elimination of a much greater percentage (∼15%) of the MIC genome, and is subject to epigenetic regulation. About 6,000 “internal eliminated sequences” (IESs) ranging from 0.5 to >20 kb in length, are removed, and the flanking macronucleus destined sequences (MDSs) are ligated. IES excision can occur reproducibly at a specific region or at a small number of alternative positions to remove most repetitive, non-genic sequences from the macronucleus. The discovery of an RNAi pathway involving 28 nt “scan RNAs” (scnRNAs) in genome rearrangement of *Tetrahymena* [21], [22] demonstrated that the mechanism by which IES-containing chromatin is targeted for elimination is strikingly similar to the mechanism by which centromeric heterochromatin is targeted for silencing in other organisms and provides a conserved mechanism by which ‘foreign’ genetic elements that invade eukaryotic genomes are identified for silencing or elimination[21]–[25]; see Figure 8 in [26] for the most recent description of this process.

A third type of genome reorganization occurring during conjugation is ribosomal gene amplification. During MAC development, the single-copy gene encoding the 28S, 17S and 5.8S ribosomal RNAs (rRNAs) is excised (via flanking CBS), rearranged to form a palindromic dimer, capped with telomeres and amplified to a final copy number of ∼9,000, ∼200-fold more than each of the other MAC chromosomes (for review and references, see [27]). These *Tetrahymena* macronuclear minichromosmes (referred to collectively as rDNA) provide a rich source of telomeres (half of the telomeres in the cell) and a well-characterized replication origin, and have been exploited to create autonomously replicating, high copy number transforming plasmids for antisense and over-expression studies (for review and references see [28]–[30]). Because it is a single copy gene in the germline micronucleus, the ribosomal RNA gene in *Tetrahymena* can also be analyzed by conventional Mendelian genetics [31].

With the maturation of molecular genetic technologies in *Tetrahymena* [32]–[34], publication of the *T. thermophila* macronuclear genome sequence [35] and establishment of the *T. thermophila* Genome Database (TGD; www.ciliate.org) [36], new opportunities have opened up to address fundamental questions of biology using genomic techniques in this organism. DNA microarray technology offers the possibility to study gene expression on a genome-wide scale, and rapid advances are being made toward understanding the transcriptional programs of several model organisms. Here we describe a user-friendly microarray system for genome-wide analysis of gene expression in *Tetrahymena*. We provide baseline data for expression of all annotated *Tetrahymena* putative genes during all three major stages of the *Tetrahymena* life cycle: growth, nutrient starvation (a presumably recurring condition in their natural habitat as well as a required condition for the next stage) and conjugation (the sexual stage of the life cycle). We also identify non-transcribed, constitutive, and extremely highly transcribed open reading frames during the three stages, as well as differentially induced and stage specific ORFs. Because conjugation offers special opportunities for studying nuclear differentiation, we have concentrated on identifying putative genes whose expression is specific to, or highly up-regulated, during conjugation. We have also utilized previous studies of gene expression during conjugation to demonstrate the validity of the microarray platform and to identify genes that are co-expressed with genes of known function during this process, as well as new cohorts of co-expressed genes. These studies strongly support the belief that most of the large number of predicted genes in *Tetrahymena* are functional, provide a striking picture of the remarkable degree of control of mRNA abundance during conjugation, and identify a number of candidate genes for further study of both the nuclear and non-nuclear events of conjugation.

## Results and Discussion

### Validating the *Tetrahymena* microarray platform

Microarray platforms that utilize oligonucleotide DNA probes corresponding to predicted open reading frames (ORFs) have provided a reproducible approach to analyze changes in mRNA levels, allowing meaningful analyses of patterns of gene expression in a variety of cellular systems and model organisms [37]. The first-generation predictions of ORFs based on the nearly complete sequence of the *Tetrahymena thermophila* macronuclear genome have recently become available [35], enabling microarray-based genome-wide analysis of mRNA abundance in different stages of the life cycle in this model organism. Because the *Tetrahymena* genome is extremely AT-rich and this represents the first genome-wide microarray analysis in any ciliate species, we felt it necessary to establish the validity of this microarray platform.

#### Technical and biological reproducibility

We assessed the genome-wide reproducibility between independent hybridizations of replicates of the same samples. Each of the samples of the first growth culture (L1-l, L1-m, L1-h, ∼100,000, 350,000 and 1,000,000 cells/ml respectively) was split into two and hybridized to two independent arrays on different days. In all 3 cases, the *r* ^2^ value of a genome-wide comparison of expression values between independent hybridizations of the same growth sample was >0.99 (data not shown), indicating that the fabrication of the microarrays and the hybridization procedures are highly reproducible.

We also compared reproducibility among biological replicates. We were able to compare a total of 50 replicates of 20 different experimental conditions (see Materials and Methods for details). Three stages of growth, each done 3× gave pairwise *r* ^2^ values of 0.90–0.96 (data not shown); seven time points during starvation, each done 3× gave *r* ^2^ values of 0.90–0.97 (data not shown) and ten time points during conjugation, each done 2× gave *r* ^2^ values of 0.93–0.97 (Table 1). Detailed examination of Table 1 demonstrates the utility of using the r^2^ values to monitor the suitability of combining data sets. Clearly, the pairwise r^2^ values from the same time points during conjugation give the highest values, indicating that the timing of the two experiments was similar. We conclude that, while the r^2^ values of biological repetitions are clearly not as high as the technical repetitions, the correlations are high on a genome-wide basis, and that it might be possible to combine and average them in subsequent analyses. However, additional analyses were required to justify averaging the data for individual genes (see below).

**Table 1:** Comparison of variation between two independent biological replicates.

Samples	C1-0	C1-2	C1-4	C1-6	C1-8	C1-10	C1-12	C1-14	C1-16	C1-18
**C2-0**	***0.93***	0.76	0.75	0.70	0.74	0.77	0.77	0.81	0.84	0.84
**C2-2**	0.71	***0.96***	0.89	0.82	0.76	0.76	0.71	0.77	0.76	0.74
**C2-4**	0.69	0.84	***0.96***	0.89	0.82	0.78	0.76	0.79	0.79	0.76
**C2-6**	0.66	0.76	0.85	***0.94***	0.91	0.84	0.79	0.79	0.80	0.77
**C2-8**	0.69	0.72	0.76	0.82	***0.95***	0.93	0.88	0.86	0.85	0.83
**C2-10**	0.75	0.76	0.77	0.78	0.89	***0.97***	0.94	0.91	0.89	0.87
**C2-12**	0.73	0.78	0.78	0.77	0.85	0.93	***0.95***	0.92	0.89	0.87
**C2-14**	0.77	0.80	0.83	0.81	0.87	0.90	0.94	***0.98***	0.95	0.92
**C2-16**	0.80	0.79	0.81	0.81	0.87	0.89	0.91	0.95	***0.97***	***0.95***
**C2-18**	0.80	0.74	0.76	0.76	0.83	0.87	0.88	0.91	0.94	***0.95***

10 samples were taken from different time points in two conjugation series (C1 and C2). Numbers represent r^2^ values. The highest r^2^ values between points in the two different experiments are indicated in bold and italics.

The two conjugation experiments in Table 1 were done by the same person (WM) at the University of Rochester. Although the manufacturer of the microarrays (Roche NimbleGen) does not recommend it, we also examined the correlation between these 2 experiments and a third one done, nominally under the same conditions, in the Pearlman laboratory at York University (Table S1). The pairwise correlations between experiments done in the two different laboratories are not as good as those done in the same laboratory, but they are still high. Although only the two experiments illustrated in Table 1 were used for the more detailed analyses of conjugation presented below, the conclusions based on using data from all 3 biological replicates are highly similar to the ones described here (REP, unpublished observations). All of the data sets used in this paper are publicly available at NCBI Gene Expression Omnibus (GEO; accession numbers are shown in Table S11).

#### Background subtraction and normalization

**Figure 1:**
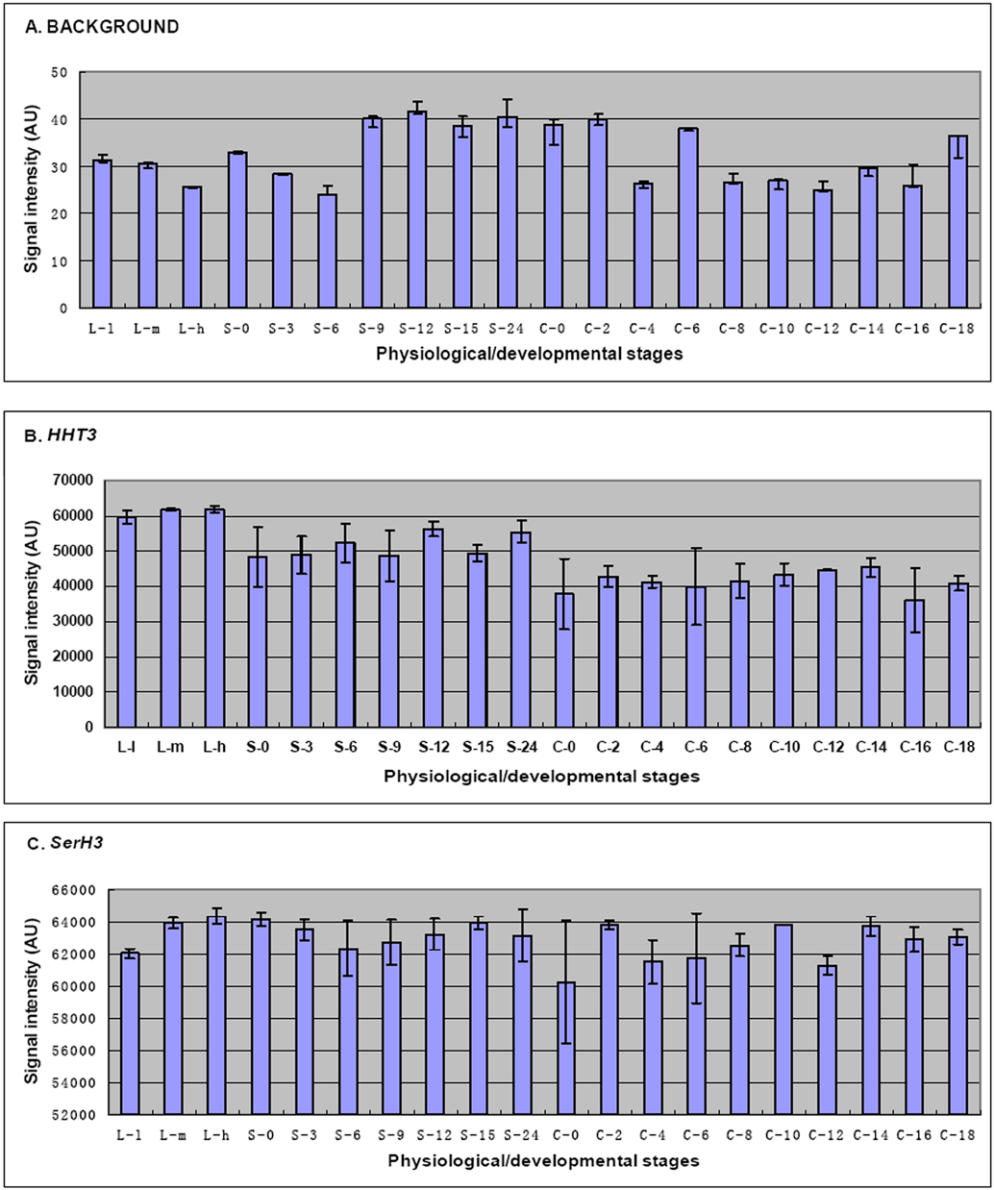
Background determination and normalization controls. (A) Background signal intensities during growth (L-l to L-h), starvation (S-0 to S-24) and conjugation (C-0 to C-18) stages were determined using 4308 different random probes on each array. The signal intensities shown for these probes are the average of 12924 values for triplicate growth and starvation samples and 8616 values for duplicate conjugation samples. The bars represent the standard errors. (B, C) Relative levels of *HHT3* and *SerH3* mRNAs at all 20 stages. The results shown here are the average of triplicate growth and starvation samples and duplicate conjugation samples, and the bars represent the standard errors.

**Figure 2:**
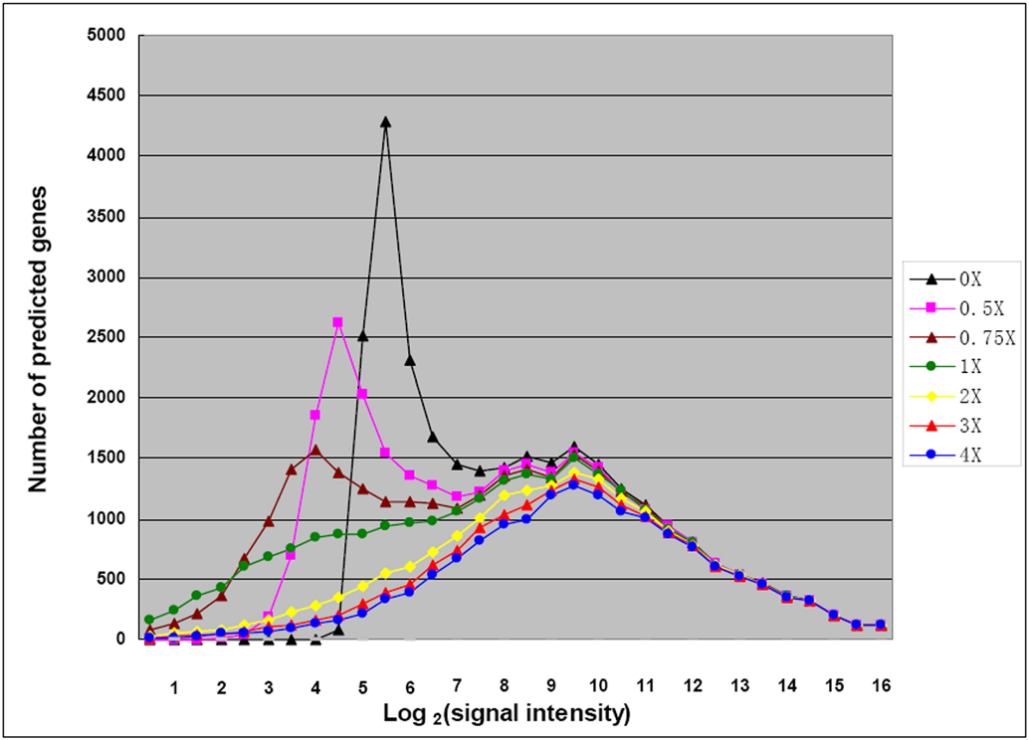
Microarray-wide distribution of signal intensities as a function of the level of subtraction of the average signal intensity shown by 4308 unrelated (negative control) array probes. As described under Materials and Methods, each curve was obtained by subtracting, from the signal intensity of every probe, the particular multiple of the negative control signal intensity (33 AUs, see Fig. 1A) shown in the box, according to the approach described in Wei [38]. Note that at 3× subtraction, the distribution has converged to a robust profile that changes little at >3× subtraction and retains no hint of the large non-specific hybridization observed in the un-subtracted distribution. This determination was done using the 2 hr conjugation sample.

We wished to establish that the microarray platform described here produced reliable results that correlated with global measurements of gene expression and analyses of expression of specific genes performed in *Tetrahymena* by other methods. To this end, we established background levels of hybridization (negative controls) and demonstrated, at the level of individual genes, that the expression values obtained were reproducible among biological replicates. To estimate non-specific, background binding, included on each array were 4308 randomly generated oligonucleotide probes comparable in length and GC content to the experimental probes on the array. The low, average mean signal for these negative probes, 33 arbitrary units (AU; Figure 1A), represents the best estimate of methodological background–due to preparation of samples, array manufacture and processing–but does not indicate to what extent probes on the array might cross-hybridize weakly to the labeled RNA sequences. We estimated these weak cross-hybridizations (as described under Materials and Methods, using the approach described in [38]) by plotting the distribution of signal intensities at different levels of background subtraction (see Figure 2 for the 2 hr conjugation sample). As was also observed by Wei [38] for sea urchin microarrays, at 3× subtraction the distribution in the *Tetrahymena* microarray has converged to a robust profile that retains no hint of the very large non-specific hybridization peak observed in the unsubtracted distribution. Thus 3× subtraction of the negative control background seems sufficient to remove the vast majority (if not all) of the methodological and non-specific cross-hybridization background. Wei et al [38] were able to conclude from independent data available in sea urchins (similar data are not available in *Tetrahymena*) that this 3× subtraction was not likely to eliminate signals from known transcripts of very low abundance. Based on all these findings, we define 3× the methodological background (99 AUs) as “1× corrected background” in subsequent analyses.

The Roche NimbleGen microarrays are normalized to enable comparisons among arrays [39]. To confirm that relative signals among the microarrays were accurately normalized, we checked two genes (*HHT3*, a replacement histone H3 variant and *SerH3*, a cell surface antigen) that have been reported to be expressed constitutively at approximately constant levels throughout all physiological/developmental stages. The normalized values from each microarray are relatively constant at all stages for both of these genes (Figure 1B, C). These results, coupled with the similar background levels among all 50 microarrays, argue that signals for a given mRNA can be reliably compared among all of the stages we have analyzed.

#### Comparison of microarray data with northern blots

**Figure 3:**
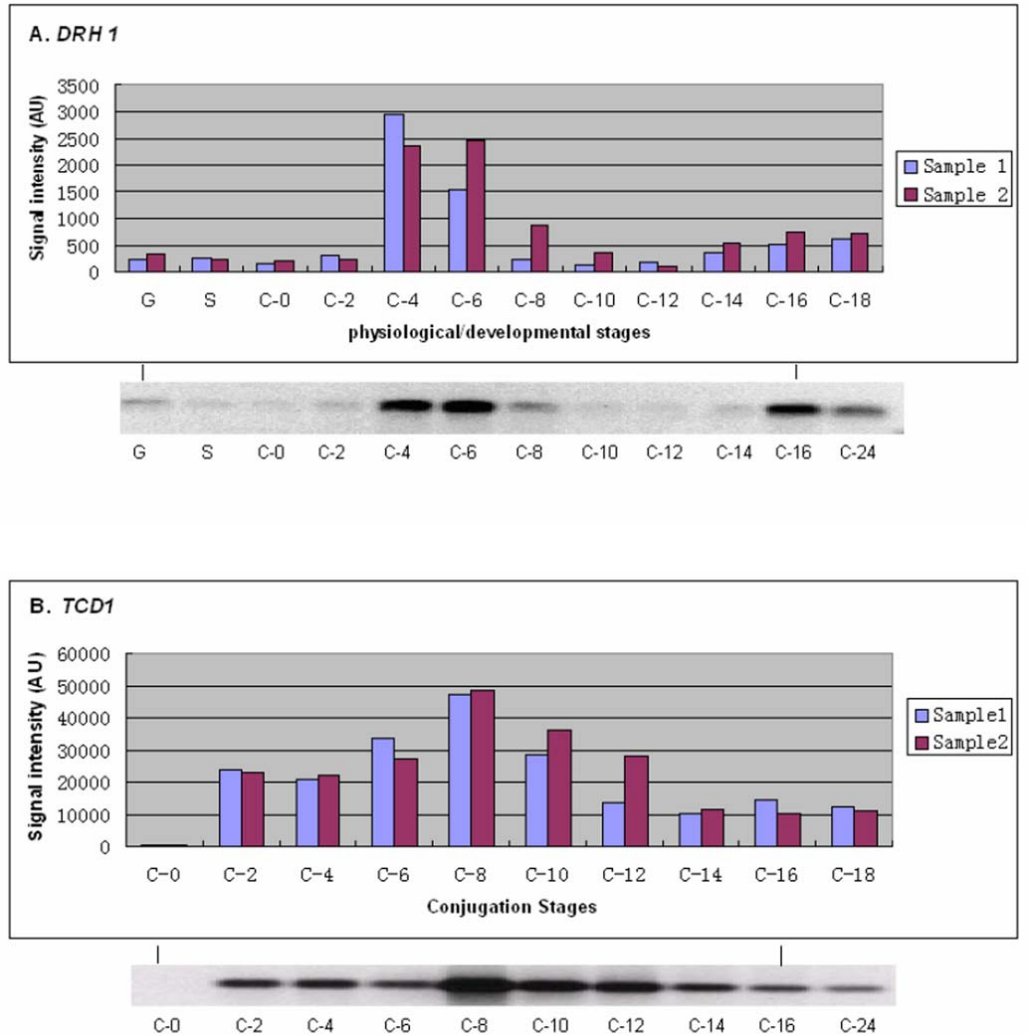
Comparison between microarray and northern blot expression profiles. Top panel, microarray data; lower panel, northern blots from independent RNA preparations and hybridizations. A: *DRH1*, encoding a putative helicase (J. Bowen, unpublished), B: *TCD1* encoding a putative chromodomain protein (W. Wang, unpublished). Values from individual conjugation experiments are shown to indicate the reproducibility of the expression of specific genes in replicate experiments. G, growing cells; S, starved cells; C, conjugating cells at 0 to 24 hr after mixing of two different mating types.

Microarray data from seventeen genes (*DCL1, TWI1, CnjB, DRH1, TCD1, ERI1, LIA1-5, tBRG1, CHD3, CHD7, ASF1, PDD1,* and *ARP1)* were compared with northern blots. For fifteen of the seventeen genes, microarray data matched northern blot results closely, as illustrated for *DRH1* and *TCD1* genes in Figure 3A, B. For *LIA2* the two methods differed in that expression was detected in starved cells and in early conjugation in the microarray data, but not in the northern blot, and *ARP1* showed differences between the northern blots and the microarray data only in starved cells. Thus, as in other studies [38], the expression of specific genes determined by microarrays correlates well, but not perfectly with measurements by other methods. As in those studies, the exact basis for the occasional discrepancies between methods is not clear. We conclude that, for most *Tetrahymena* genes, the temporal patterns of expression derived from the microarray data are likely to be accurate.

#### Distinguishing “true” co-expression from cross-hybridization among closely related genes

In *T. thermophila*, ∼40% of the predicted genes have at least one paralogue with detectable similarity, and many genes are members of large multigene families. Although the software that designs the oligonucleotide probes attempts to maximize the mismatch between related genes, it is still possible that probes designed to hybridize to one member of a gene family will detect expression from another, causing an over-estimate of the expression of one or more genes in the family. This becomes particularly important when apparent co-expression is observed for genes having high coding sequence identity and when estimating the fraction of *Tetrahymena* ORFs that are transcribed.

In practice, it is difficult, if not impossible, to identify, by microarray data alone, which specific cases of apparent co-expression are solely due to cross-hybridization. We attempted an estimate based on actual probe-transcript sequence identity among duplicated genes in the *T. thermophila* genome (Table S10 in [35]), using the conservative estimate that 70% sequence identity could result in cross-hybridization. Among the 474 genes in all 16 families with 27–100 members, we found that, on average, a maximum of less than 20% of expressed genes in families could be accounted for by cross-hybridization (Table 2). When corrected for the fraction of genes not in families, less than ∼8% of all expressed genes could be false positives for gene expression. The actual fractions are probably significantly lower, because of the following factors that make this estimate a likely *upper limit* to the fraction of false positive expressed genes. 1) 70% probe-transcript sequence identity is a very conservative low threshold for seeing above-background signal intensity due solely to cross-hybridization. By examining the effect of random mismatches, Hughes *et al.* [40] showed that, for 60-nucleotide long probes, on average the contribution of cross-hybridization rises above background only when the sequence identity between probe and transcript exceeds 80% (12 mismatches). Furthermore, we have seen examples of expressed vs. unexpressed pairs where the probe-transcript sequence identity was as high as 94% without any sign of cross-hybridization. 2) Use of the maximum value of the reciprocal probe-transcript sequence will also count, as false positive, every gene whose probe shows lower signal intensity and <70% sequence identity to the predicted transcript of the pair member with higher signal intensity. This combination cannot be readily explained by cross-hybridization, and neither of these two genes is likely to be false positive. 3) In many cases, the signal intensity of both members of a co-expressed gene pair represents true expression of both, even when showing probe-transcript sequence identity >70% and thus possibility of cross-hybridization. For example, in relatively large clusters of such genes it seems unlikely that all but one would be non-expressed genes. 4) Expressed genes are under-represented in gene families (as described elsewhere in this article), so that we should multiply by a factor smaller than 0.4 when correcting the fraction of false positives for the fraction of genes not in families. Possible false positives that would not be counted would be non-expressed genes giving cross-hybridization with a related gene which is expressed during one set of time-points as well as with another relative which is expressed during a different set of time-points. In this case, the false positive would not show apparent co-expression with either related gene. Such special cases would likely be rare.

**Table 2:** Upper estimate of the fraction of false positive expressed genes.

			Clusters of co-expressed gene pairs (R>0.9)					
Gene Family	Genes in the family	Expressed genes	Clusters	Genes	Range of coding sequence identity	Range of probe-transcript sequence identity	Maximum number of false positive genes*	Maximum fraction of false positive genes**
Cysteine proteinase	83	69	3	33	37 – 100%	16.1 – 100%	28	41%
Interaptin family 1	68	58	5	19	4.5 – 94.4%	1.7 – 98.3%	10	17%
Interaptin family 2	51	5	1	2	13.7 – 14.5%	40 – 43.3%	0	0%
Hypothetical protein family 1	50	37	4	22	6.1 – 98%	1.7 – 100%	18	49%
Histidine kinase DhkL	48	42	2	19	2.5 – 56.7%	15 – 68.3%	0	0%
Proprotein convertase	47	2	0	0	-	-	0	0%
Hypothetical protein family 2	43	42	2	5	4.5 – 54.1%	25 – 56.7%	0	0%
Probable serine/threonine-protein kinase	43	43	9	27	51.1 – 98.6%	1.7 – 100%	18	42%
Hypothetical protein family 3	41	6	1	2	52.1%	26.7 – 48.3%	0	0%
ABC transporter AbcG1	39	26	3	8	16.5 – 66.9%	15 – 61.7%	0	0%
Cytochrome P450 monooxygenase	34	26	1	4	4.7 – 99.9%	35 – 100%	2	8%
Hypothetical protein family 4	33	25	2	8	38.2 – 90.4%	15 – 93.3%	3	12%
MORN repeat protein	33	33	4	10	26.7 – 42.7%	23.3 – 78.3%	4	12%
Hypothetical WD-repeat protein alr3466	29	21	2	4	55.5 – 78%	33.3 – 93.3%	2	10%
ATP-binding cassette transporter C4	27	17	1	2	45.5%	26.7 – 31.7%	0	0%
Immobilization antigen LD	27	22	5	11	16.3 – 96.2%	26.7 – 100%	4	18%
Total		474	44	175			89	18.8%
Corrected for only 40% of genes in gene families								7.5%

* Threshold: probe-transcript sequence identity >70%.

** Obtained by dividing the number in the previous column by the total number of expressed genes in the family

A more detailed analysis of the influence of cross-hybridization on apparent expression or co-expression is beyond the scope of this genome-wide survey. In special cases, when scientific interest warrants the additional effort, other tests may be available to test true expression. Examples of such tests are: real time quantitative PCR when paralog-specific PCR primers can be designed, and northern blot analysis when the transcripts have distinguishable lengths [41]–[44].

### Global analyses of gene expression

#### The number of transcribed genes

Early hybridization-saturation studies demonstrated that a large fraction of the *Tetrahymena* genome was transcribed into polysomal RNAs in growing and starved cells, leading to the estimate that ∼45,000 different mRNAs were expressed [45]. This estimate must be revised downward to ∼26,000 mRNAs with the recent demonstration that the macronuclear genome size is ∼1.04×10^7^, [35], not ∼1.8 ×10^7^ bp as assumed in [45]. This new estimate also is subject to experimental error and does not include any genes whose transcription is conjugation specific. Initial analysis of the *Tetrahymena* macronuclear genome sequence revealed the existence of >27,000 putative open reading frames [35]. This estimate is subject to considerable computational error, owing to a relative lack of ESTs with which to train the gene finder programs. Also, while both of these estimates are quite similar, they are surprisingly large. We sought therefore to use our microarray data to provide an independent estimate of the total number of *Tetrahymena* ORFs that are transcibed in any of the 3 major physiological/developmental stages of the *Tetrahymena* life cycle.

We compiled a search program (Intel Visual Fortran Compiler, version 6.5, Compaq Computer Corporation, Houston, TX) (Figure S1), designed to calculate the maximum and minimum expression values of putative ORFs in the growth, starvation and conjugation samples respectively, and then to set up definable search conditions. A search was done to identify all of the gene models whose maximum expression at all 3 life-cycle stages was less than 1× corrected background (99 AU), conditions requiring that these genes be unexpressed, or expressed at extremely low levels; 5876 (22%) putative ORFs were identified that can be considered as candidate non-transcribed genes. When the 5876 predicted protein-coding genes were used in a Blastp search of the NCBI database, 2421 genes (41%) had matches with E values less than 1e-5 , indicating the existence of a possible ortholog, and 3455 genes (59%) had values greater than 1e-5, indicating that they shared little or no similarity with known proteins.

The subset of 2421 putatively non-transcribed genes encoding proteins that had significant similarity to other proteins is unlikely to include non-coding regions of random sequence mis-identified as ORFs by the gene finder [46]. Of these, 1749 (72%) were members of multigene families (Table S2), significantly more than the 39% of all genes found in gene families in the entire genome [35]. The absence of detectable transcripts indicates that these genes are either undergoing pseudonization or that they are potentially active genes that are expressed under specialized conditions. For example, some members of the Cytochrome P450 and Glutathione S-transferase families might be expected to have an important function requiring that they be transcribed only in the presence of certain pollutants or toxicological agents that were not present in our laboratory culture conditions [47], [48].

We determined whether any of the 3455 predicted ORFs that lacked significant overall similarity to known proteins (E-values greater than 1e-5) were likely to be actual genes or, instead, were more likely to have been wrongly predicted by the gene finder. Sixty-two of them had a domain with some similarity to a previously described domain in the database. In addition, Blastp and EST searching showed 237 of the ORFs had at least one EST; 8 ORFS had both an EST and a conserved domain (Table 3). Thus, these 291 ORFs could be real genes. The remaining 3164 predicted ORFs (∼11.5% of the total predicted ORFs) were not detectably transcribed during growth, starvation or conjugation in *Tetrahymena,* lacked similarity to known proteins or known protein domains and were not represented in the EST databases.

**Table 3:** Summary of non-transcribed ORFs lacking homology to known proteins (E values>1e-5).

	Number of ORFs	ORFs with an EST	ORFs with a Conserved domain
<50 aa	717	13	0
50–100 aa	801	41 a	1^a^
>100 aa	1937	183 b	61^b^
All	3455	237	62

a One gene had both an EST and a conserved domain.

b Seven genes had both an EST and a conserved domain.

In summary, the microarray analyses provide direct evidence for the existence of transcripts from ∼21,100 predicted genes. This number could be even higher if some of the putative non-transcribed genes are expressed under conditions other than growth, starvation or conjugation. The number of expressed genes could be somewhat lower if there is cross-hybridization between transcribed and non-transcribed members of multigene families, but we have presented evidence arguing that this is likely to occur in only a small fraction of genes. These observations indicate that most of the large number of predicted ORFs in the *Tetrahymena* genome are indeed transcribed genes.

#### Constitutively expressed genes

We next sought to identify genes that were expressed at high and relatively constant levels in all 3 physiological/developmental stages. This analysis should provide the first estimate of the number of “housekeeping” genes in a ciliate and identify expression markers for comparison of the expression of other genes. Identification of the most highly expressed genes, should also identify strong promoters for constitutive expression of transgenes encoding homologous or foreign proteins. The number of such genes is extremely high, even if a stringent cut-off of 5× corrected background is used (Table 4).

**Table 4:** Search conditions and number of constitutively expressed genes.

Expression level*	Gene No.
>2×	9016
>5×	5281
>10×	3229
>50×	939
>100×	524
>250×	95
>500×	12

* Genes were included if their minimum level of expression at every time during every stage (growth, starvation and conjugation) exceeded the stated multiple of corrected background.

Within the 95 putative genes whose signal intensities were more than 250× corrected background (Table 4), fifteen genes had E values on BLASTp search to the GenBank non-redundant database greater than 1e-5. The gene annotations of the other 80 genes are shown in Table S3: 26 of them (32.5%) are ribosomal proteins; five are tubulin-tyrosine ligase family proteins; five are papain family cysteine protease-like proteins; four are associated with translation elongation factors; five are associated with cilia (*ATU1*, *BTU2*, *CAM1*, *FTT18*, *FTT49*); three are CARD15-like proteins; two are secretory granule lattice proteins; two are histones (*HHT3, HHT4*); two are associated with membrane fusion and fission events (AAA family ATPase, *RAB1A*). Others are ATP synthase, *CAT1,* eukaryotic aspartyl protease; inorganic pyrophosphatase; and *SerH*. All of these highly, constitutively expressed genes are involved in essential/important cellular processes of metabolism or cell growth. All 12 genes whose signal intensity is more than 500× corrected background gave expression profiles similar to those illustrated in Figure 1B and C, providing evidence that the normalization methods accomplished their intended purpose. The promoters of these genes are strong candidates for use in constitutive expression of foreign proteins.

#### Changes in gene expression during growth, starvation and conjugation

**Figure 4:**
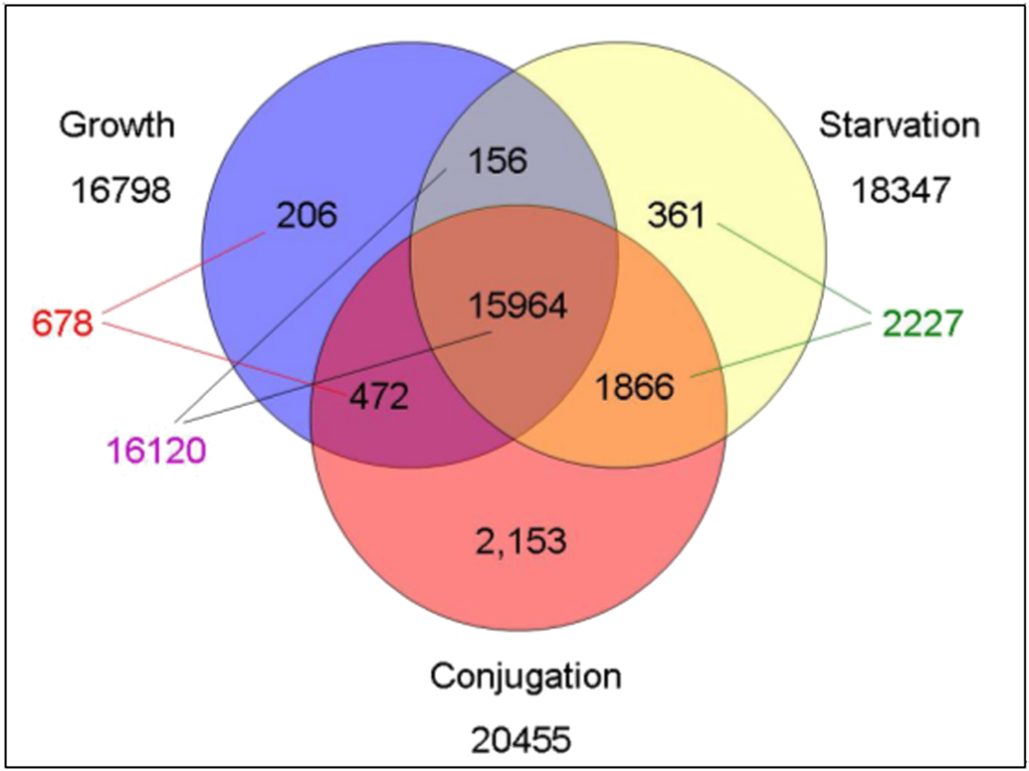
Number of genes expressed in at least one time point during each of the three major stages of the *T. thermophila* life cycle. Expression is defined as signal intensity above 1× adjusted background (99 AUs), as described in the text. The number of genes expressed at each of the stages is shown beneath the identifier for each circle. Number of genes in composite categories discussed in the text (e.g., genes expressed during growth but not starvation) are indicated along the margins. A total of 21,178 genes are accounted for in the diagram. An additional 5,876 genes failed to show signal intensity >99 AUs at every time point and stage tested (discussed in the text). One gene had a value of exactly 99 AUs and consequently was excluded by the search criteria. The search conditions were those listed in Table S4.

Growth and starvation are commonly studied physiological states in *Tetrahymena* and starving cells also undergo a series of developmental changes that are prerequisites for conjugation to occur. During starvation, *Tetrahymena* cells also experience a morphological transformation in which they elongate and develop a long caudal cilium and swim rapidly [49], [50], presumably to facilitate dispersion as an adaptive strategy in seeking either a mate or richer environment. We sought to determine the extent of changes in gene expression that occur during these physiological/develolpmental states. Based on the earliest global studies of RNA hybridization in *T. thermophila*, it was argued that, of the total number of genes expressed in growing or starved cells, ∼80% were expressed in both conditions while ∼20% were specific to one of the two stages being compared [45]. Consistent with this proposal, the microarray analyses (Figure 4, Table S4) showed that ∼85% (16120) of the putative ORFs were expressed at both stages. Interestingly, greater than three times as many genes were expressed specifically during starvation (2227) as were expressed specifically during growth (678). Of the 2227 putative genes expressed in starvation, but not during growth, mRNAs from 1866 (84%) of them were also detectable during conjugation, and many (1118, nearly 50% of them) were present during the first three hours of starvation. It is important to emphasize that starvation initiates the first steps in the sexual stages of the life cycle of *T. thermophila*, during which cells change rapidly from vegetative cells unable to mate to mating-competent cells [12], [13], and that the sexual phase of the life cycle can proceed to completion in starved cells. These considerations likely account for many of the large number of mRNAs that are present in starved and early conjugating cells, but not in growing cells. Interestingly, a similar number of genes (2153, described below) are expressed specifically during conjugation.

**Figure 5:**
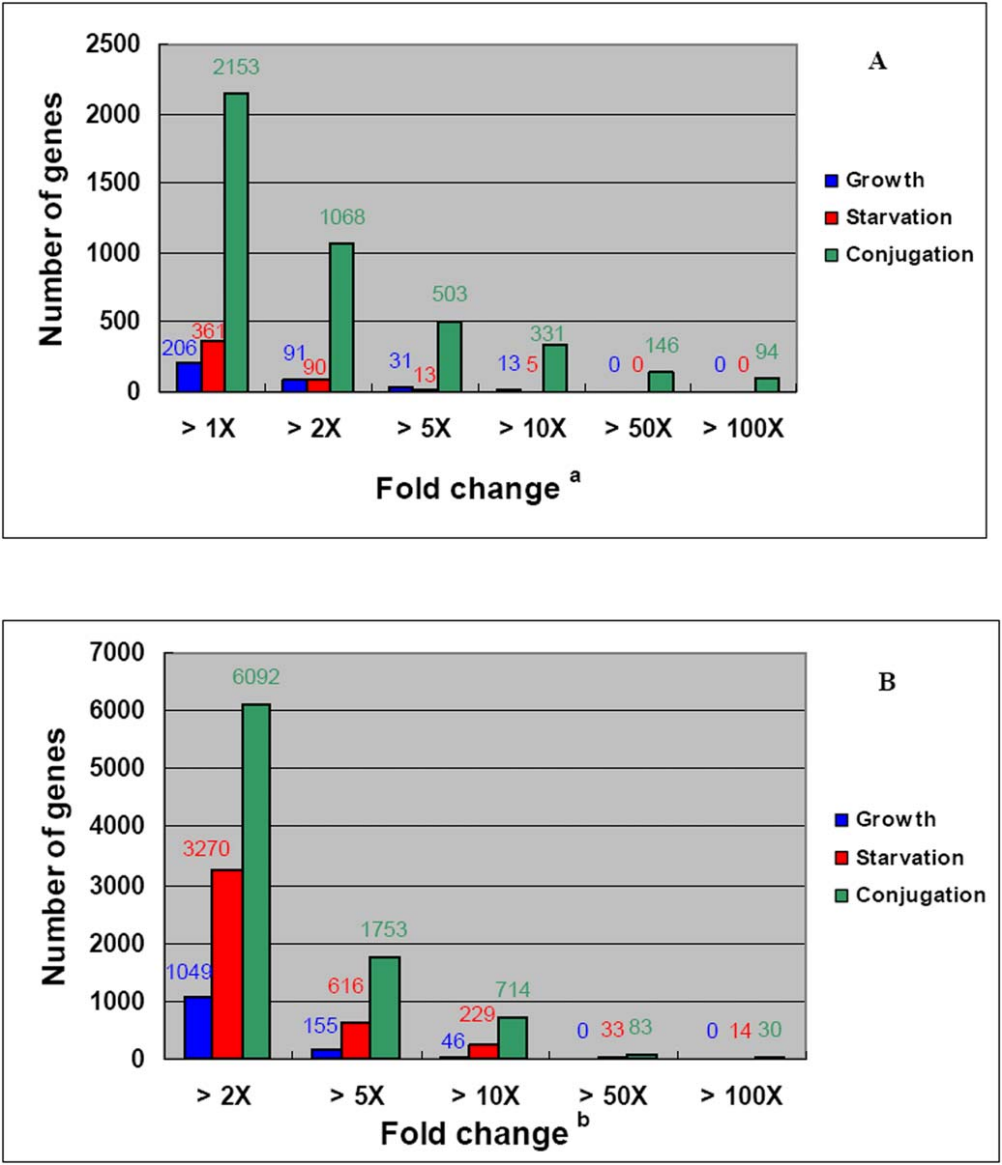
Comparison of numbers of predicted genes specifically expressed (A) or upregulated (B) during growth, starvation and conjugation. ^a, b^: the search conditions were those listed in Table S5.

We used the search program to identify predicted genes that were specifically expressed (i.e., hybridization was detectable only) during growth, starvation or conjugation, or that were significantly up-regulated during these stages (see the search conditions in Table S5). In Figure 5, we have plotted the number of genes showing different levels of specific or up-regulated expression. The most striking feature of this analysis is the large number of genes whose expression is specific to, or highly induced (>5×) during conjugation and the remarkably high levels of expression of these induced/expressed genes. Thus, almost 500 genes are expressed at levels >5× corrected background during conjugation. That more genes are expressed during conjugation than during either growth or starvation (Figure 4) may not be so surprising if one considers an analogy to multicellular animals: ciliate conjugation encompasses meiosis, production of haploid pronuclei, pronuclear fusion and events with likely parallels to events occurring in early embryonic stages in animals (eg, histone transitions, chromatin remodeling, initiation of rRNA and mRNA transcription, cytoplasmic determination of germ-line and somatic nuclear lineages).

**Figure 6:**
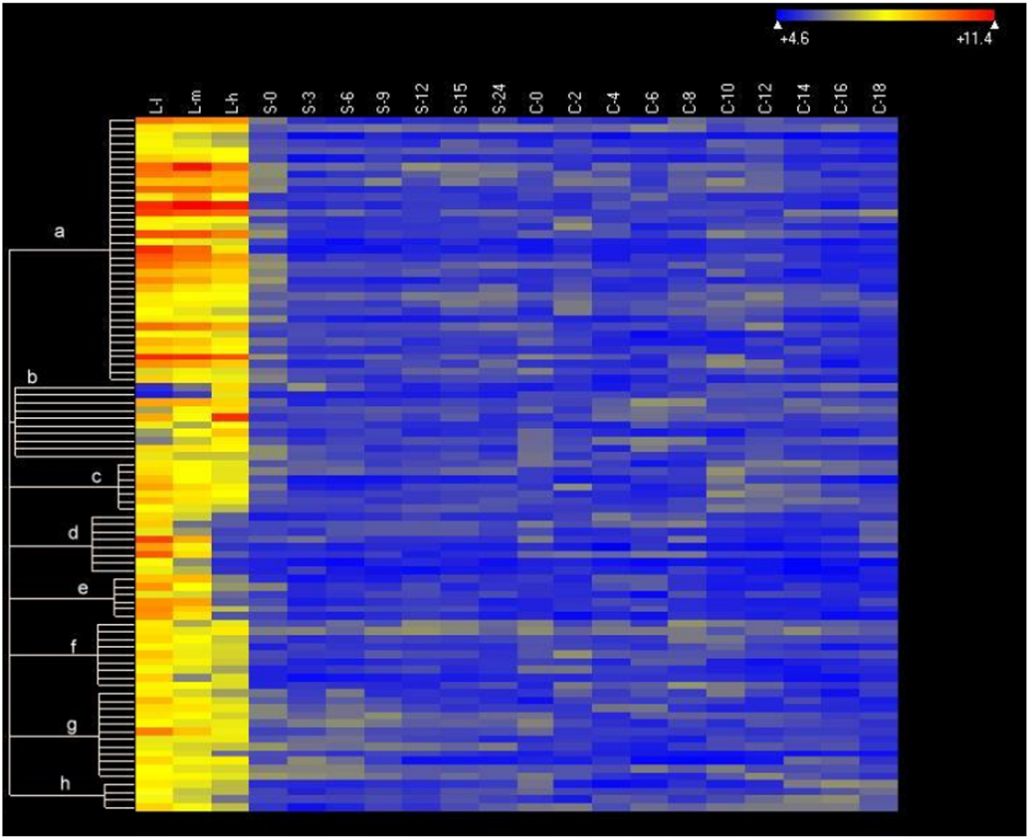
Heat map of the expression of 91 growth-specific genes. Genes expressed at levels >2× corrected background (see Figure 5A) are included. Clustering done using ArrayStar 2 (Clustering type: K-mean, Distance metric: standard Pearson). The heat map uses colors to display the relative values of all tiles within a given experimental condition wih blue indicating low expression, yellow indicating intermediate expression and red indicating high expression. The numerical values give the actual values on a log 2 scale that are associated with each color. Stages are as described in Materials and Methods. The color scale is shown by the bar at the top right corner of the figure.

**Figure 7:**
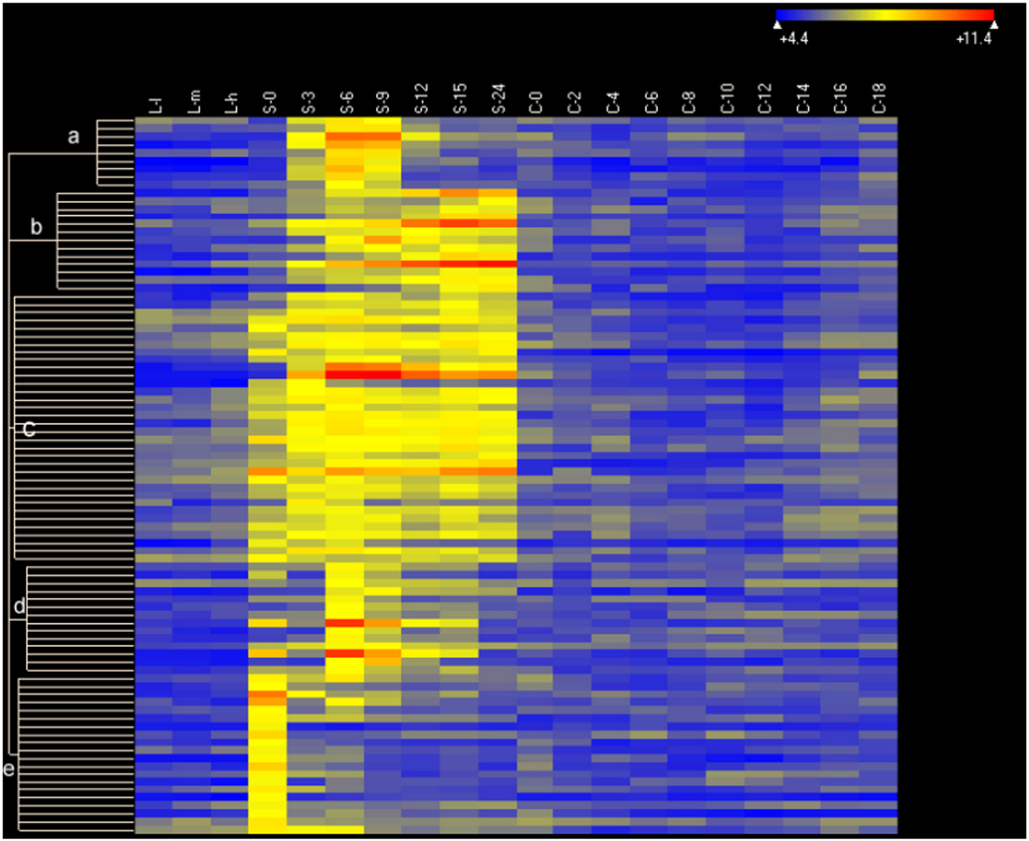
Heat map of 90 starvation-specific genes. Genes expressed at levels >2× corrected background (see Figure 5A) are included. Clustering parameters, conditions and other symbols are as in Figure 6.

**Figure 8:**
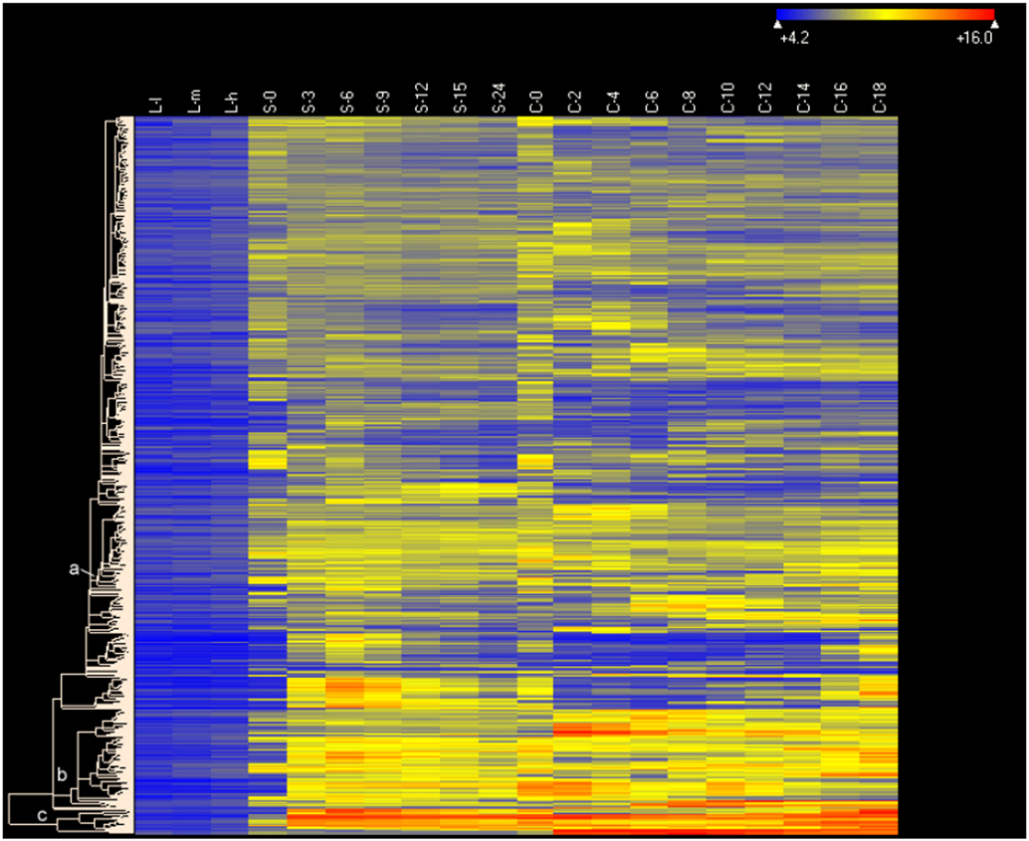
Heat map of 706 genes expressed during both starvation and conjugation but not during growth. Genes whose maximum expression levels during starvation and conjugation were >2× corrected background, and were less than 1× corrected background during growth, were included, Clustering type: Hierarchical, with Euclidean distance metric. Software, conditions and other symbols are as in Figure 6.

**Figure 9:**
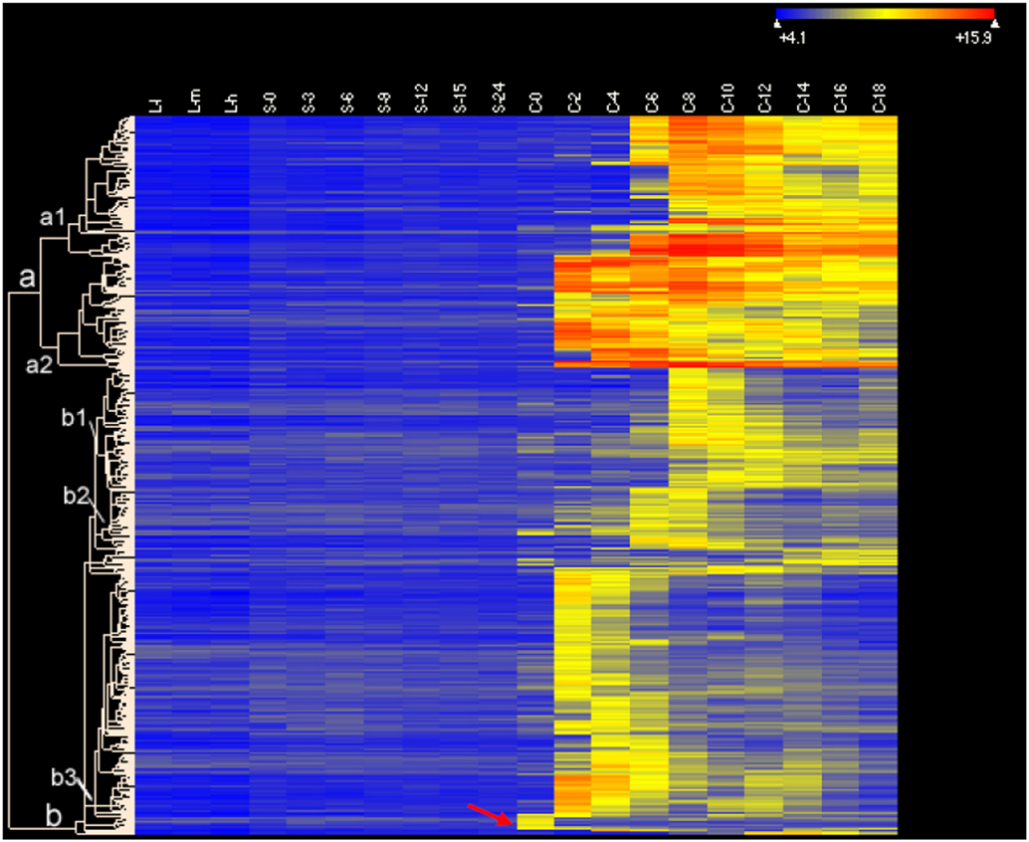
Heat map of 503 conjugation-specific genes. Genes expressed at levels >5× corrected background (see Figure 5A) were included. Clustering parameters, conditions and other symbols are as in Figure 6. The red arrow indicates 8 genes that are expressed almost exclusively at C-0.

Heat maps and cluster analyses indicate that groups of genes in growing, starved and conjugating cells have distinct patterns of expression (Figures 6, 7, 8, 9). In growing cells (Figure 6), analyses of 91 growth-specific genes indicate that there are eight distinct clusters. Genes in cluster a are expressed relatively uniformly in L-l, L-m and L-h stages of growth (see Materials and Methods for a characterization of these stages). Genes in clusters c, d, e, f, g, and h are expressed in L-l stage growing cultures but the expression of these genes declines as growth proceeds. The disappearance of transcripts in L-h cells of clusters d and e is particularly striking, suggesting these genes are not expressed in stationary phase cells. Genes in cluster b show increased expression as the culture grows. The specific genes associated with each of these clusters are listed in Table S6; their continued investigation should provide insights into the specific changes that accompany altered growth states in *Tetrahymena*.

Figure 7 illustrates the patterns of genes expressed specifically during starvation. Genes in cluster a are induced between 0 and 6 hr after starvation is initiated and their gene products largely disappear after ∼9 hr. The transient expression of these genes suggests they are not required for long-term viability during starvation and could be involved in the initial stages of conversion of vegetative cells to mating-competent cells which is known to occur during the first few hours of conjugation [12], or in the morphological conversion to slender cells with an elongated cilium. The accumulation of products of genes in cluster b initiates at about the same time as in cluster a, and continues to be expressed throughout the 24 hr the cells were starved. Some of the genes in this cluster (Table S7) are likely to be involved in adaptation to starvation conditions and/or for long-term survival in the absence of nutrients. Genes in cluster c initiate expression in S-0 cells, indicating they are induced very rapidly during the short time required to wash the cells out of growth medium, re-suspend them in Tris buffer and then pellet them prior to isolating RNA. Their expression also continues through the later stages of starvation; these genes could have functions that overlap with those in clusters a and b. Genes in cluster d are expressed transiently, a little later than those in cluster a. Genes in cluster e are expressed almost exclusively in S-0 cells, suggesting that their brief expression is a stress response caused by centrifugation and/or the transfer from growth medium to Tris buffer. The rapid disappearance of a number of growth-induced genes in S-0 cells (Figure 6) may also reflect this stress response. A number of genes whose expression is specific to starvation encode proteins that could function in signal transduction pathways (eg., nucleotide binding proteins, kinases, ubiquitin hydrolases; see Table S7).

Interestingly, a large number of genes (>700) that are not expressed during growth are expressed during both starvation and conjugation (Figure 8). At least some of the genes in cluster c encode proteins that are thought to function specifically in conjugation such as *CnjB* (Noto, Bednenko and Gorovsky, unpublished observations) and *Lia 6* [51], indicating either that these genes have multiple functions or, more likely, that preparation for the expression of some proteins required in large amounts during conjugation begins during starvation.

**Figure 10:**
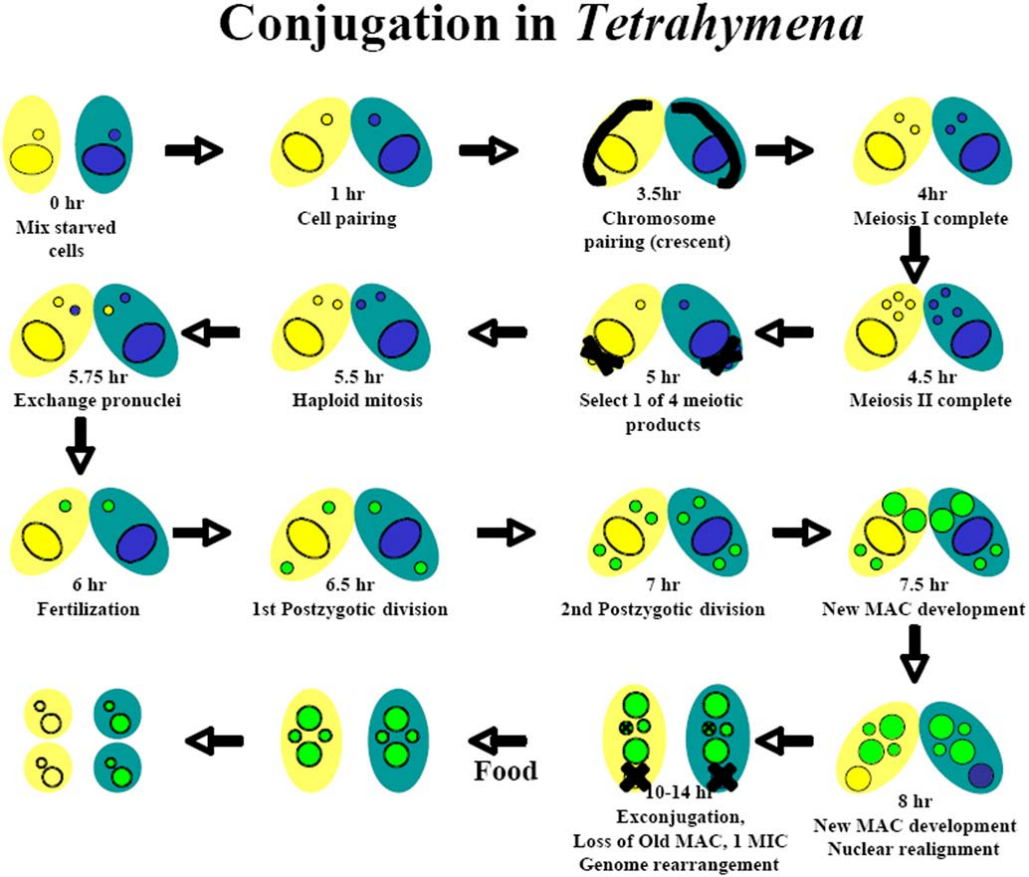
Stages of conjugation in *Tetrahymena.*

Figure 9 illustrates the heat map and cluster analysis of 503 conjugation-specific genes. A number of distinct patterns of expression can be observed. Eight genes (indicated by the red arrow) are expressed almost exclusively at C-0, suggesting that they respond rapidly and transiently to the mixing of cells of different mating types. Genes in cluster a show high levels of conjugation-specific expression and, once expression is initiated, are expressed for long periods. Included in this cluster are previously characterized, conjugation-specific genes, including *DCL1* [52], [53], *TWI1* [7], *PDD2* [54], [55], *PDD3* [56], *GIW1* (K. Mochizuki, personal communication), *LIA1*, *LIA3* and *LIA5* [51]. Three other published conjugation genes, *PDD1* [57], *ASI1* [58] and *ASI2* [59] were found in the conjugation-induced genes (data not shown). Thus, all of the published and known conjugation genes were found in either the conjugation-specific or conjugation-inducible genes. Cluster a can be further divided into 2 sub-clusters. Most genes in cluster a2 initiate expression between 0 and 2 hours after cells are mixed, when the early steps in RNAi mediated IES elimination [7], [52], [53] and in meiosis are initiated (Figure 10). Most genes in cluster a1 initiate expression between 4 and 6 hours, during later stages of meiosis, pronuclear formation, nuclear exchange and fertilization (Figure 10), and when the scanning events of IES elimination are occurring [21]. Most genes in the b cluster are expressed at lower levels than those in the a cluster and are expressed more transiently. Most genes in cluster b3 initiate expression between 0 and 2 hr and transcripts from these genes have largely disappeared by 8 hr. RNAs from most genes in cluster b2 begin accumulating between 4 and 6 hr after cells are mixed and disappear between 10 and 12 hr. Genes in cluster b1 begin being expressed shortly after those in cluster b2 (between 6 and 8 hr). RNAs from some of the genes in this cluster become undetectable by 14–16 hr while others are detectable up to 18 hr, when our analysis was terminated.

It is clear from these heat map analyses of gene expression during conjugation that a large number of genes are expressed specifically during conjugation and that there are clusters of genes that exhibit distinct patterns of expression. In studies described below, we examine the co-expression patterns of genes in an effort to identify candidate genes involved in specific processes during conjugation.

#### Expression of codon biased genes

**Figure 11:**
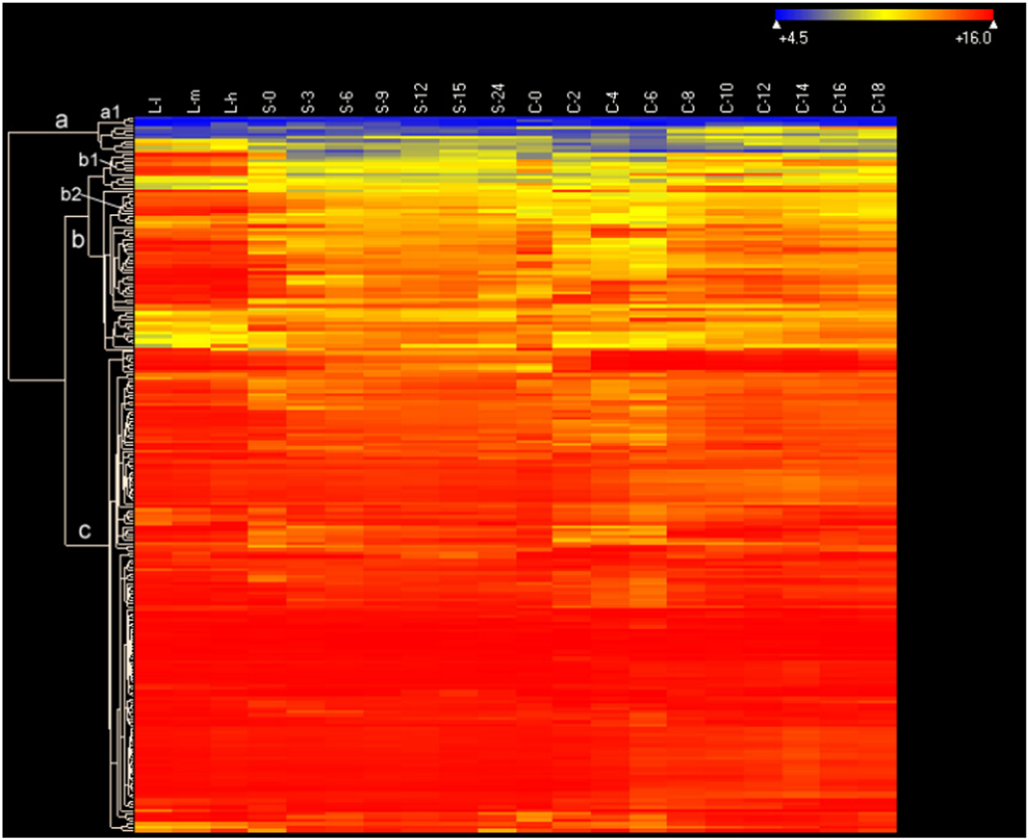
Heat map of 217 codon biased genes described in reference [35]. Clustering parameters, conditions and other symbols are as in Figure 6.

Analysis of the codon usage of predicted ORFs in the sequenced macronuclear genome [35] identified a subset of 232 genes that utilized a codon set that differed from that of the average gene. Marked codon biases are thought to be associated with mRNAs that are translated more rapidly or more accurately than average messages. These genes were represented in the EST database, on average, ∼15× more frequently than other genes, suggesting that a likely function of this codon bias is promoting more efficient translation of abundant proteins. Consistent with this suggestion, many of the genes in this subset encoded housekeeping proteins. Sixty-seven of these codon-biased genes lacked ESTs, leading Eisen *et al.* [35] to suggest they were either falsely predicted or might need to be transcribed rapidly and/or efficiently at some specific stage of the *Tetrahymena* life-cycle. As our microarray analyses covered a wide range of physiological/developmental stages and are subject to less bias than non-saturated, random analyses of cDNAs, we examined the expression of 217 of the 232 codon-biased genes that were included in the microarray design (Figure 11). Ninety-five percent of these genes (clusters b and c) showed high expression, especially the 146 genes (67.3%) in cluster c (Figure 11). Most were highly expressed during all stages. Only 3 genes in cluster a1 (TTHERM_00648580, TTHERM_00283180 and TTHERM_00654000; nomenclature as per http://www.ciliate.org) were not detectably transcribed at any of the stages examined; all 3 are likely to be wrongly predicted genes or wrongly designed probes (WM, unpublished observations). Thus, these codon-biased genes are mostly constitutively expressed, highly transcribed genes. Interestingly, of the 939 genes that are constitutively expressed at >50× corrected background, only 133 of them also show strong codon biases. A similar comparison of all genes constitutively expressed >100× corrected background indicates that only 99 of them (18.9%) also show codon biases. Thus, while most codon-biased genes are expressed constitutively and at high levels, not all highly expressed genes are codon-biased.

#### Transcription of genes containing selenocysteine codons

**Figure 12:**
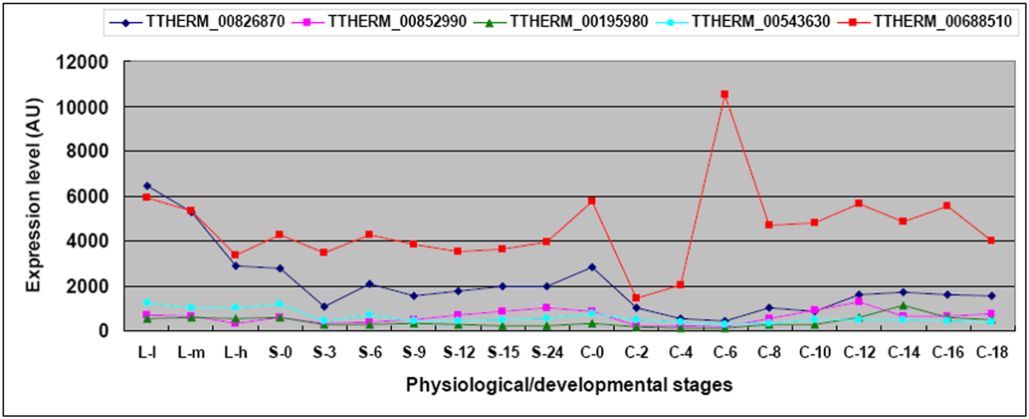
Expression profiles of five putative selenocysteine genes. Stages are as described in Materials and Methods.

*Tetrahymena* nuclear genes utilize the canonoical stop codons UAA and UAG to encode glutamine, leaving UGA as the only known termination codon in the nuclear genetic code of this organism [60]. *Tetrahymena* contains a transcribed gene encoding a tRNA with an anticodon that could recognize the UGA stop codon. This tRNA is acylated and can be labeled with radioactive selenium [61], making it highly likely that, in some *Tetrahymena* proteins, UGA encodes selenocysteine. Six genes likely to encode proteins containing selenocysteine were identified based on the presence of an in-frame UGA codon and putative stem-loop sequence motif in their 3′ untranslated regions that is characteristic of selenocysteine containing genes [35]. Five of the 6 genes were included on the microarray. To determine if these putative selenocysteine genes are co-ordinately regulated, we examined their expression patterns (Figure 12). Clearly, the genes are expressed at all physiological/developmental stages of the life cycle and are not coordinately regulated. Consistent with this, the selenocysteine tRNA has also been shown to be expressed at all physiological/developmental stages (Figure S4 in [35]).

#### The relationship between chromosomal organization and gene expression

In small artificial chromosomes of *S. cerevisiae*, genes placed near telomeres exhibit a phenomenon known as telomere position effect (TPE) in which their expression is repressed, with the level of repression decreasing with distance from the telomere (for a review of TPE, see [62]). TPE also occurs in some, but not all *S. cerevisiae* natural chromosomes and in other organisms as well. It is particularly important in some protozoans (eg., trypanosomes, *Plasmodium*) where it plays a major role in regulating genes involved in antigenic variations that function in evading host immunological defense mechanisms [63], [64]. The sequence and structure of *Tetrahymena* telomeres resembles that of yeast, and *Tetrahymena* telomeres can protect the ends of artificial chromosomes in *S. cerevisiae* and can serve as substrates for the addition of yeast telomere sequences by the yeast telomerase [65], [66]. Based on these considerations, we sought to determine whether gene expression in *Tetrahymena* macronuclei exhibited any evidence of TPE or other chromosome-associated mechanisms of gene regulation.

**Figure 13:**
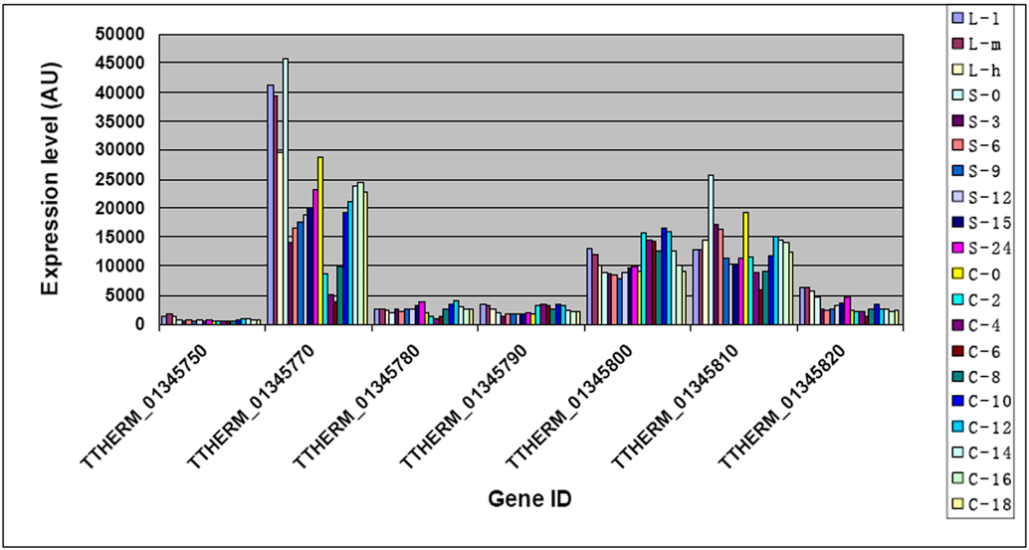
Expression of all seven genes in scaffold CH670435 during growth, starvation and conjugation. The genes are given in order of their locations on the scaffold with TTHERM_01345750 located at one end and TTHERM_01345820 at the other end. Stages are as described in Materials and Methods.

During conjugation, the 5 chromosomes, each present in two copies in the diploid micronucleus, are fragmented into ∼225 chromosomes during macronuclear development, and then endoreplicated to ∼45 ploid in the vegetative macronucleus. Scaffolds corresponding to 123 of these chromosomes have been sequenced almost entirely and contain telomeres on both ends (R. Coyne, personal communication). We examined the patterns of transcription of genes along 30 of the smallest of these “closed” macronuclear scaffolds, where the effects, if any, of telomeres on transcription of adjacent genes might be expected to be most obvious. The smallest of these scaffolds (CH670435) is only ∼38 kb and contains just 7 genes (Figure 13). On the 30 scaffolds we examined, genes transcribed at low, intermediate and high levels are found within 1–5 kb of telomeres and there is no obvious relationship between the physiological/stage-specific transcription of genes and their proximity to telomeres. Thus, we could find no evidence for a consistent pattern of telomere-related silencing or regulation of adjacent genes.

The physiological significance of the fragmentation of the small number of micronuclear chromosomes to give numerous, smaller macronuclear chromosomes is not clear. One possibility is that the existence of smaller linkage groups in the transcriptionally active macronucleus facilitates the co-ordinate regulation of genes on the same chromosome. To test this, we examined whether there was any evidence for chromosome-level regulation of gene expression. The 20 genes on scaffold CH670398 were widely distributed along the entire length of the scaffold and were all very weakly transcribed, indicating the possible existence of a chromosome-wide mechanism of transcriptional inhibition (data not shown). However, this appears to be an unusual case since among the 30 chromosomes we examined, some of which were larger and some smaller than scaffold CH670398, most contained genes exhibiting wide variations in their levels and stage-specificity of expression (data not shown). We also sought to determine whether adjacent genes or genes in the same chromosome had a tendency to show similar physiological/stage-specific patterns of expression, indicative of any chromosomal or sub-chromosomal level of gene regulation, by comparing the R values for all pairs of genes on each of the 30 chromosomes. There was no evidence that adjacent genes or genes on the same chromosome had a high likelihood of sharing the same expression patterns (data not shown). Thus, we found little evidence that chromosome fragmentation in *Tetrahymena* leads to a high level of co-ordinate regulation of genes on the same chromosome.

### Analysis of co-expressed genes during conjugation

**Figure 14:**
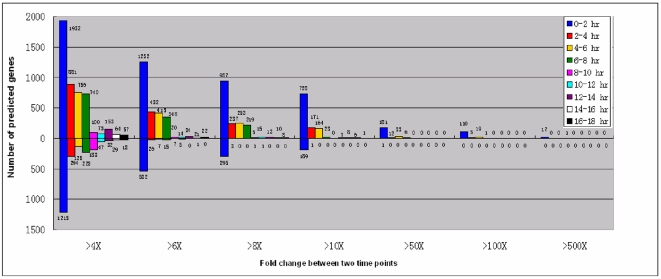
Summary of the number of genes differentially expressed at two hour intervals during conjugation. Values above and below the “0 line” represent the number of up-regulated and down-regulated genes, respectively.

One goal of our comprehensive microarray analyses of gene expression was to identify candidate genes involved in the striking developmental changes (cell pairing, meiosis, fertilization, RNAi-mediated scanning of MIC-specific sequences, chromosome fragmentation, telomere addition, rDNA amplification and the DNA splicing events of IES elimination) that occur during conjugation. As a first step toward this end, we divided the conjugation process into a series of 2 hr intervals and determined the number of genes showing significant changes in expression, either up or down from the beginning to the end of the time interval, ranging from 4 to 500 fold (Figure 14). Clearly, the first two hours of conjugation are marked by changes, both upward and downward in the expression of a large number (>3100) genes, with expression of >700 genes increasing >10× and expression of 17 genes increasing a remarkable >500× in only 2 hr. Subsequent intervals also show changes in large numbers of genes, although the numbers and extent of the changes diminish as conjugation proceeds.

Given the large number of genes showing significant increases and decreases in expression throughout conjugation, and the fact that some processes occur contemporaneously (e.g., the early, middle and later stages of RNAi-mediated IES targeting overlap respectively with meiosis, fertilization, and gene activation during macronuclear development), we sought other, more graphic methods to identify co-expressed genes associated with specific processes. Analyses of clustered heat maps are one approach to identifying co-expressed genes but, as recently discussed [67], heat maps have serious limitations in presentation, interpretation and in establishing statistical robustness. As an alternative approach, we identified individual genes, either known or likely to be involved in specific processes, and then identified additional genes whose expression was highly correlated with expression of those genes.

#### 

##### TWI1

**Figure 15:**
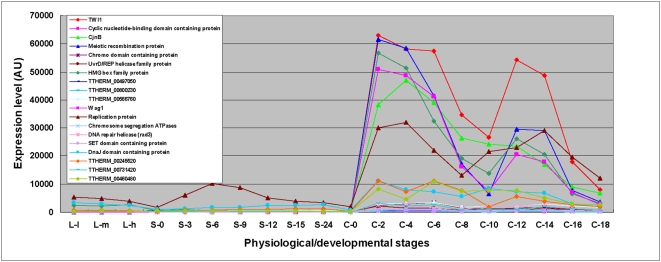
The expression profiles of *TWI1* (TTHERM_01161040) co-expressed genes. Stages are as described in Materials and Methods.

The first gene we examined was *TWI1*, which encodes an essential argonaute family protein that is associated with the small RNAs (scnRNAs) required for targeting the IES sequences for elimination [7], [21]. This gene has the added advantage that four proteins (CnjBp, Wag1p, Ema1p and Giw1p) have been shown to be physically associated with Twi1p by co-immunoprecipitation and TAP-tagging ([26], J. Bednenko, K. Mochizuki and M. Gorovsky, unpublished observations), allowing a test of whether co-expression can identify genes that encode proteins that likely have shared functions. Figure 15 illustrates the expression pattern during growth, starvation and conjugation of *TWI1* and of 18 other proteins whose expression patterns correlate highly (R>0.9) with *TWI1* (Table 5). Both *CnjB* and *WAG1* are found among these 18 genes, and expression of a third gene, *EMA1*, that encodes a protein known to be physically associated with Twi1p is also highly correlated (R = 0.85 ), but *GIW1* was not found (R<0.8). Of the eighteen *TWI1* co-expressed genes (R>0.9), 6 had no known homologs, while the other 12 were all homologous to proteins associated with DNA-related properties. These results indicate that co-expression analysis is able to identify genes that have shared functions, arguing that the other 16 genes listed in Table 5 are candidates for further investigation into the process of IES elimination.

**Table 5:** *TWI1* (TTHERM_01161040) co-expressed genes (R>0.9).

Gene ID	R	Gene annotation	E Value
TTHERM_00729090	0.96	Cyclic nucleotide-binding domain containing protein	0.0
TTHERM_01091290	0.95	*CnjB*	0.0
TTHERM_00459230	0.94	Meiotic recombination protein DMC1/LIM15 homolog	5e-81
TTHERM_00822220	0.94	Chromo domain protein	0.0
TTHERM_00829440	0.93	UvrD/REP helicase family protein	0.0
TTHERM_00155590	0.93	HMG box family protein	0.0
TTHERM_00497050	0.93	Predicted Tetrahymena ORF a	-
TTHERM_00800230	0.93	Predicted Tetrahymena ORF a	-
TTHERM_00566760	0.93	Predicted Tetrahymena ORF a	-
3691.m01119 b	0.93	*WAG1* c	0.0
TTHERM_00106890	0.93	Replication protein	3e-37
TTHERM_01295290	0.92	Viral A-type inclusion protein	5e-06
TTHERM_00688780	0.92	DNA repair helicase (rad3)	0.0
TTHERM_01108540	0.91	SET domain containing protein	3e-50
TTHERM_01013150	0.91	DnaJ domain containing protein	6e-132
TTHERM_00245520	0.91	Predicted Tetrahymena ORF a	-
TTHERM_00731420	0.91	Predicted Tetrahymena ORF a	-
TTHERM_00460480	0.90	Predicted Tetrahymena ORF a	-

a Similar to a *Tetrahymena thermophila* “hypothetical protein” but no significant similarity found to proteins in other organisms in other species in a BLASTP search of NCBI non-redundant protein database.

b Gene ID from TIGR database released in August 2006: ftp://ftp.tigr.org/pub/data/Eukaryotic_Projects/t_thermophila/annotation_dbs/interim_annotation_release_08313006/. All other gene IDs from TGD database: http://www.ciliate.org/.

c J. Bednenko, K. Mochizuki and M. Gorovsky, unpublished observations.

##### DCL1

To determine whether all of the genes expressed during the earlier stages of RNAi-mediated DNA elimination were expressed similarly, we examined the genes that were co-expressed with *DCL1*, which encodes a dicer-like protein (Dcl1p). Dcl1p localizes only to the micronucleus during meiosis and is required in the early stages of conjugation for cleavage of the double stranded RNAs produced in the micronucleus into the 28 nt scnRNAs [52], [53]. In contrast, Twi1p is found only in the cytoplasm and in the old and developing macronuclei, where it associates with the scnRNAs. Thus, it is likely that these proteins function sequentially in the early stages of IES targeting and do not interact directly. We identified 12 genes whose expression was highly correlated (R>0.9) with that of *DCL1* (Table 6). Two proteins (TTHERM_00585180 and TTHERM_00086720) contain chromodomains, a protein motif known to interact with histone modifications (H3 methylated at either K9 or K27); one of these has been shown to be required for IES elimination (Wang and Gorovsky, unpublished data). None of the genes co-ordinately expressed with *DCL1* was among the ones whose expression was highly correlated with that of *TWI1* (Table 5). Thus, these co-expression analyses identify two distinct classes of co-expressed genes, ones whose products are candidates for co-ordinate function with Dcl1p, and a second set of candidates that are more likely to function with Twi1p.

**Table 6:** *DCL1* (TTHERM_00284230) co-expressed genes (R>0.9).

Gene ID	R	Gene annotation	E value
TTHERM_00102760	0.99	Predicted Tetrahymena ORF ^a^	-
TTHERM_00721440	0.96	Predicted Tetrahymena ORF ^a^	-
TTHERM_00721450	0.96	Ser/Thr protein phosphatase family protein	0.0
TTHERM_00197660	0.95	TPR Domain containing protein	0.0
3686.m00954 ^b^	0.93	Predicted Tetrahymena ORF ^a^	-
TTHERM_01367700	0.93	Predicted Tetrahymena ORF ^a^	-
TTHERM_00471710	0.93	Predicted Tetrahymena ORF ^a^	-
TTHERM_00585180	0.92	Heterochromatin protein	0.0
TTHERM_00193790	0.91	EF hand family protein with WD40 domain	7e-31
TTHERM_01014470	0.91	Cell cycle switch protein with WD40 domain	5e-121
TTHERM_00222270	0.91	PHD-finger family protein	0.0
TTHERM_00086720	0.90	Chromo domain protein	5e-05

Footnotes a and b as in Table 5.

##### EZL1

*Tetrahymena EZL1* encodes an H3 methylase that likely catalyzes scnRNA-dependent K27 methylation in vivo and is required for IES elimination [68]. There were 51 *EZL1* co-expressed genes whose R values were more than 0.99, and 579 genes whose expression showed a correlation with that of *EZL1* greater than 0.9 (data not shown). Three proteins (encoded by *ESC1*, *RNF1* and *RNF2*) have been identified as components in an Ezl1p complex (Y. Liu, unpublished data). They were all included in the top 25 *EZL1* co-expressed genes (Table 7), demonstrating again that co-ordinate expression of genes can identify genes with protein products that function together. It remains to be determined which of the other genes that are co-ordinately expressed with *EZL1* also function with this gene. Also, the mechanisms behind this remarkable level of co-ordinate expression of such a large number of genes warrant further investigation.

**Table 7:** *EZL1* (TTHERM _00335780) co-expressed genes (25 highest R values).

Gene ID	R	Gene annotation	E value
TTHERM_00678460	0.999	Predicted Tetrahymena ORF ^a^	-
TTHERM_00219320	0.998	Predicted Tetrahymena ORF ^a^	-
TTHERM_00729020	0.998	Predicted Tetrahymena ORF ^a^	-
TTHERM_00790880	0.997	Linear amide C-N hydrolases, choloylglycine hydrolase family	3e-67
TTHERM_00483640	0.997	Serine/Threonine protein kinases (the CDC2 subfamily of the protein kinase CDK family )	2e^-^180
TTHERM_00442420	0.997	ESC1 with WD40 domain c	0
TTHERM_01285910	0.997	B-box zinc finger family protein	0.011
TTHERM_00927370	0.996	TPR Domain containing protein	6e-48
TTHERM_00637350	0.996	RNF1 with zinc finger c	0.0
TTHERM_00348540	0.995	Histidine kinase-like ATPases	0.0
TTHERM_01276320	0.995	Predicted Tetrahymena ORF ^a^	-
TTHERM_00695710	0.995	Transcription factor E2F	4e-138
3700.m01689^ b^	0.995	Predicted Tetrahymena ORF ^a^	-
TTHERM_00265150	0.994	ABC transporter family protein	0.0
TTHERM_00437650	0.994	DNA polymerase family B containing protein	0.0
TTHERM_00295910	0.994	Predicted Tetrahymena ORF ^a^	-
TTHERM_00375160	0.994	Predicted Tetrahymena ORF ^a^	-
TTHERM_00732690	0.994	Cyclic nucleotide-binding domain containing protein	3e-20
TTHERM_00370670	0.994	Predicted Tetrahymena ORF ^a^	-
TTHERM_00522660	0.994	RNF2 (Chromosome segregation ATPases) c	0.0
TTHERM_00161310	0.994	Coiled coil protein	1e-04
TTHERM_01194740	0.993	Histidine acid phosphatase family protein	1e-14
TTHERM_00841270	0.993	Predicted Tetrahymena ORF ^a^	-

Footnotes a and b as in Table 5.

c Liu, unpublished data

##### ASF1

Because of our interest in histones and their deposition onto chromatin [44], we examined the expression pattern of *ASF1,* which encodes a conserved histone (H3 and H4) chaperone that is involved in both nucleosome assembly and disassembly and has been functionally implicated in chromatin replication and repair, DNA damage checkpoint control, nucleosome disruption and replacement during transcription and heterochromatin formation and gene silencing [69]. The *Tetrahymena ASF1* homolog (THERM_00442300) is expressed at low levels in growing cells, at barely detectable levels in starved cells, and is highly induced between 2 and 10 hr of conjugation, during meiosis and post-zygotic DNA replication. Eighty-six genes are co-expressed with *Tetrahymena ASF1* (R>0.9). Amongst the genes whose expression is most highly correlated with that of *ASF1*, many are involved in DNA replication, sister chromatid cohesion and separation (Table 8). They represent a set of co-expressed genes that is distinct from those involved either in RNAi-mediated DNA rearrangement or meiosis (Tables 5 and 6), as only 4 of the top 25 co-expressed genes were genes involved in these processes.

**Table 8:** *ASF1* (TTHERM_00442300) co-expressed genes (25 highest R values).

Gene ID	R value	Gene annotation	E value
TTHERM_00442300	1	ASF1 c	0
TTHERM_01048090	0.99	SMC family, C-terminal domain containing protein	0
TTHERM_00277550	0.98	MCM2/3/5 family protein	0
TTHERM_00283330	0.98	*CET1*	0
TTHERM_00474670	0.98	Mitochondrial carrier protein	0
TTHERM_00204150	0.98	Zinc binding protein with RAD 18 domain [Ciona intestinalis]	2e-06
TTHERM_00297160	0.97	Separase, a protease involved in sister chromatid separation [Schizosaccharomyces pombe]	7e-26
TTHERM_00245200	0.97	RNA binding motif protein 35A isoform 5 [Homo sapiens]	7e-34
TTHERM_00647510	0.97	Predicted Tetrahymena ORF ^a^	-
TTHERM_00962200	0.97	Importin-beta N-terminal domain containing protein c	0
TTHERM_00398070	0.97	Predicted Tetrahymena ORF ^a^	-
TTHERM_00554270	0.97	MCM2/3/5 family protein	0
TTHERM_00277530	0.97	DNA replication factor Cdt1 [Culex pipiens quinquefasciatus]	4e-14
TTHERM_00773400	0.97	Predicted Tetrahymena ORF ^a^	-
TTHERM_01513300	0.97	Predicted Tetrahymena ORF ^a^	-
TTHERM_00765120	0.97	Predicted Tetrahymena ORF ^a^	-
TTHERM_00245410	0.97	heterochromatin protein 1	0
TTHERM_00046490	0.97	Tuple1/HirA with WD40 domain[Takifugu rubripes]	3e-20
TTHERM_00049080	0.97	Structure-specific recognition protein	0
TTHERM_00762900	0.97	ATPase, AAA family protein	0
TTHERM_00684590	0.97	Protein kinase domain containing protein: the Aurora protein kinase family	0
TTHERM_00161750	0.96	Predicted Tetrahymena ORF ^a^	-
TTHERM_00636920	0.96	DNA polymerase family B containing protein	0
TTHERM_00402060	0.96	Predicted Tetrahymena ORF ^a^	-
TTHERM_00101160	0.96	PREDICTED: similar to nucleoporin 210 [Monodelphis domestica]	3e-38
TTHERM_00372470	0.96	Predicted Tetrahymena ORF ^a^	-

Footnote a as in Table 5.

c R. Pearlman, unpublished observations.

Recent studies have shown that *ASF1* in human cells interacts with proteins found in the MCM2-7 complex [70]; 2 of the top 25 proteins co-expressed with *Tetrahymena ASF1* are MCM orthologs. Yeast *ASF1* has been shown to physically interact with *Hir1*, *Hir2* and *Hir3* and to interact genetically with *Rad18* (http://db.yeastgenome.org/cgi-bin/interactions.pl?dbidS000003651). Orthologs to both HirA and Rad18 are highly co-expressed with *Tetrahymena ASF1*. Most interestingly, a highly co-expressed importin (karyopherin)-beta gene has been shown to be physically complexed with *Tetrahymena ASF1* (J. Garg, J.S. Fillingham, and R.E. Pearlman, unpublished observations) and recent studies in yeast have indicated that Asf1p is also associated with a specific β-karyopherin [71]. These analyses of *ASF1* co-expression distinguish yet another distinct class of genes whose expression overlaps those described above and can identify proteins that physically interact.

#### Meiosis-specific genes

The process of meiosis temporally overlaps with the early steps in the RNAi-mediated process of IES elimination but has been little studied in *Tetrahymena*. In addition, some of the genes that were co-expressed with *TWI1* and *DCL1* were homologous to proteins that have meiotic functions in other organisms. To determine whether any genes involved in meiosis could be distinguished from genes involved in the early stages of RNAi-mediated IES elimination, we identified 54 genes that had been listed in TGD as having some similarity to meiosis-associated genes in other organisms. In addition, Mochizuki *et al.* identified 59 genes based on similarity searches with meiotic genes of other organisms [72]. These genes exhibited a variety of expression patterns, a few of which showed similarity to the patterns of *DCL1*, *TWI1* or *PDD1*, when inspected visually (data not shown). Among these putative meiotic genes, we identified 25 whose expression was highly correlated with at least 3 others, resulting in a cluster whose genes might have co-ordinate functions. Very few of these 25 meiotic genes showed highly co-ordinated expression (R>0.9) with either *PDD1*, *DCL1* or *TWI1* (Table 9), indicating that at least some of the major genes involved in IES elimination have expression patterns that can be distinguished from a subset of those likely to be involved in meiosis. One of the meiotic genes showed correlated expression with *EMA1*, a gene encoding a putative RNA helicase that has been shown to function in IES elimination [26] and another (TTHERM_459230, similar to DMC1 a protein responsible for strand exchange in meiotic recombination) showed correlated expression with *TWI1.* Interestingly, expression of *CnjB* was highly correlated with expression of 7 of these meiotic genes. While it is not clear whether this correlated expression between genes involved in meiosis and IES elimination is coincidental, because these processes are occurring concurrently, or whether some genes function in both processes, a possible function of *CnjB* in meiosis clearly warrants further investigation.

**Table 9:** Correlation coefficient between 25 meiotic genes and 5 IES elimination genes.

	*PDD1* c	*DCL1* c	*TWI1* c	*EMA1* c	*CjnB*
**TTHERM_00115410** a	0.49	0.28	0.73	0.68	0.76
**TTHERM_00636920** a	0.65	0.21	0.84	0.74	***0.92***
**TTHERM_00194810** a *	0.54	0.26	0.79	0.69	0.81
**TTHERM_00426230** a *	0.57	0.18	0.78	0.66	0.81
**TTHERM_00557810** a	0.69	0.53	0.88	***0.91***	***0.94***
**TTHERM_00564430** a	0.62	0.29	0.84	0.79	***0.93***
**TTHERM_00825440** a *	0.71	0.38	0.88	0.85	***0.95***
**TTHERM_01016020** a	0.67	0.44	0.86	0.79	0.85
**TTHERM_00150000** a	0.38	0.26	0.56	0.50	0.53
**TTHERM_00237490** a	0.75	0.27	0.86	0.71	0.84
**TTHERM_00294810** a	0.65	0.27	0.77	0.72	0.84
**TTHERM_00127000** a	0.61	0.55	0.72	0.76	0.76
**TTHERM_00011650** b	0.49	0.13	0.64	0.62	0.78
**TTHERM_00295920** b	0.68	0.27	0.89	0.77	***0.92***
**TTHERM_00297160** b	0.56	0.18	0.73	0.68	0.83
**TTHERM_00425970** b	0.58	0.20	0.68	0.64	0.79
**TTHERM_00459230** b	0.70	0.32	***0.94***	0.79	***0.92***
**TTHERM_00624870** b	0.54	0.14	0.70	0.66	0.83
**TTHERM_00684590** b	0.64	0.28	0.83	0.79	***0.92***
**TTHERM_01008650** b	0.52	0.22	0.74	0.63	0.74
**TTHERM_01179960** b	0.52	0.11	0.70	0.62	0.82
**TTHERM_00160570** b	0.56	0.20	0.63	0.61	0.75
**TTHERM_01030000** b	0.51	0.33	0.73	0.67	0.73
**TTHERM_00343420** b	0.40	0.02	0.58	0.47	0.68
**TTHERM_00382290** b	0.61	0.07	0.76	0.59	0.81

a candidate meiotic genes from TGD.

b candidate meiotic genes from Mochizuki *et al.* 2008 [67].

* overlap between ^a^ and ^b^.

c The gene ID of *PDD1, DCL1, TWI1, EMA1* and *CjnB* are TTHERM_00125280, TTHERM_00284230, TTHERM_01161040, TTHERM_ 00088150 and TTHERM_01091290 respectively.

The correlation coefficients greater than 0.9 are indicated in bold and italics.

#### Identification of candidate genes involved in the later stages of IES elimination

The early and mid stages of IES elimination, those involving the production of scnRNAs, the scnRNA-mediated mechanisms that protect MDSs and the targeting of IESs for elimination have been better studied than the later stages in DNA elimination involving the enzymatic mechanisms that actually remove the IESs and rejoin their flanking sequences. As a result, while a number of genes with known functions in these earlier stages have been identified (eg, *DCL1*, *TWI1*, *EZL1*, *EMA1*) only a single gene (*LIA1*) that functions after IESs have been targeted for H3 K9 methylation has been identified [73]. The studies described above indicate that genes involved in meiosis and in the RNAi-mediated process of IES targeting are likely to have specific patterns of expression in earlier stages of conjugation. Therefore, it seemed reasonable to turn this approach around to identify candidate genes involved in late stages of IES elimination by searching for genes with distinctive patterns of expression in the later stages of conjugation.

We observed a number of genes whose expression was limited to, or was highly induced, late in conjugation, peaking at 10–14 hr. When we examined these genes for properties that might be associated with DNA rearrangement, the most interesting one (TTHERM_01107220) showed strong similarity only to a protein found in *Paramecium tetraurelia* and to a human piggyBac transposable element. This gene also contained a domain found on the C-terminal arm of Ku70/Ku80, a conserved heterodimer that binds to DNA double strand breaks. These features strongly suggest its involvement in IES elimination. We then identified all of the genes whose expression pattern were highly correlated with TTHERM_01107220. To our surprise, 85 genes (R>0.9) fulfilled this criterion (Table S8). Preliminary annotation of these genes indicates that they have highly diverse functions (eg., multiple kinases, TPR domain proteins, WD domain proteins, zinc finger domain proteins, proteins with cyclic nucleotide binding domains and cell surface proteins), suggesting that a number of novel processes, in addition to IES removal, occur specifically in the late stages of conjugation. It is important to emphasize that these genes are not simply ones that are required for starvation or for return to vegetative growth since their induced expression is highly specific to the late stages of conjugation and is not found in the other physiological stages we have examined. One of the genes expressed late in conjugation, TTHERM_00427480, contained a domain similar (E = 2.1e-19) to a conserved Endonuclease/Exonuclease/Phosphatase domain (PF03372) as well as strong similarity (E = 1.0e-40) to a protein in *Paramecium*, another ciliate that undergoes DNA elimination. Again, these properties make it a good candidate for having a function in IES elimination. Interestingly, this gene is one of 3 highly similar, tandemly arranged genes (TTHERM_00427470, 00427480 and_00427490). Although all 3 genes are induced late in conjugation, only TTHERM_00427480 is expressed specifically during this period while the other 2 are also expressed at lower levels in growing and starved cells. Thus, this approach has yielded at least 2 strong candidates for genes involved in the later stages of IES elimination as well as identifying a large number of new genes having unexpectedly diverse functions late in conjugation. These genes warrant further investigation.

#### Conjugation-induced/specific transcription factors

**Figure 16:**
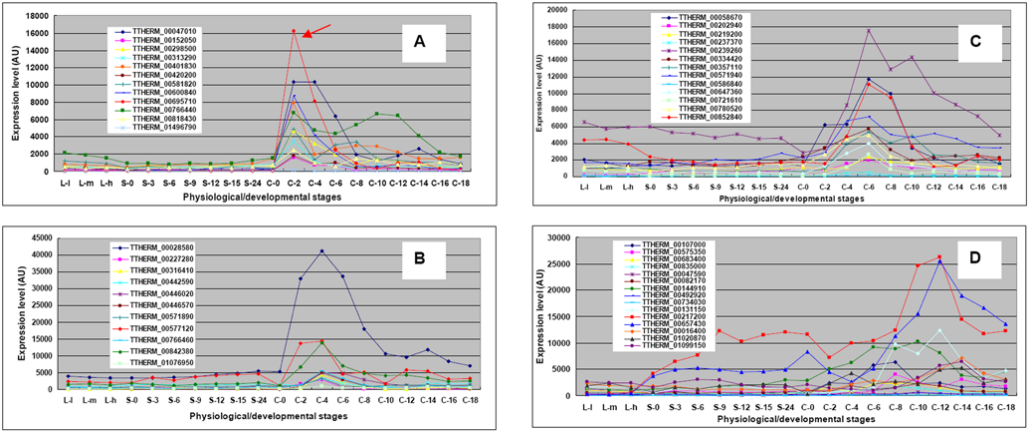
Fifty-one Conjugation-induced/specific transcription factors of *Tetrahymena* with the expressed peak at 2 hr (A), 4 hr (B), 6 hr (C), and 8–14 hr (D) postmixing. Stages are as described in Materials and Methods.

Little is known about transcription factors (TFs) in *Tetrahymena*, and no specific TFs have been associated with specific physiological or developmental stages. We examined the expression of 112 genes identified in TGD as transcription factor orthologs. When the search condition was set up to identify conjugation induced/specific genes (Max_C>2×Max_L and Max_C>2×Max_S), one gene (TTHERM_00695710), encoding a homolog to the transcription factor E2F2/E2Fc, was conjugation-specific (arrow, Figure 16A) and another fifty genes were conjugation-induced. Most of these genes were up-regulated in early conjugation (12, 11 and 13 genes with expression peaks at 2, 4 and 6 hr respectively; Figure 16A, B and C), and 15 genes were induced late in conjugation (4, 5, 3 and 3 genes with the expression peaks at 8, 10, 12 and 14 hr respectively Figure 16D).

Within the 51 conjugation-induced transcription factors (Table S9), there are a number of gene families. Nine genes encode homologs of components of transcription initiation factor TFIIH, a multi-protein complex that is part of the RNA polymerase ll transcription pre-initiation complex. TTHERM_00420200 encodes an ortholog of polypeptide 3/Tfb4p, TTHERM_00313290 encodes an ortholog of Tfb2p and TTHERM_00152050 encodes an ortholog of Ssl1p. These components of TFIIH are thought to interact with each other and show a high correlation coefficient of expression (Table 10). TTHERM_00028580 encodes a protein homologous to Spt5, a transcription factor that functions in transcription elongation and contains a KOW domain thought to bind RNA. All four of these genes are likely to be involved in regulating RNA polymerase II transcription and all show a high correlation of expression with at least one subunit of RNA polymerase II (Table 10). TTHERM_00401830 and 00818430 with high correlation coefficient of expression encode orthologs of XPB proteins, which are components of TFIIH and are responsible for DNA helicase activity during nucleotide excision repair (NER). Four members of this group (TTHERM_01496790, 00227280, 00058670, 00144910) show similarities to serine/threonine protein kinases involved in transcription initiation and show different peaks of transcription activity at 2, 4, 6 and 10 hr respectively.

**Table 10:** Correlation coefficient between nine homologs of component of transcription initiation factor (TFIIH), a homolog of Spt5 and 3 subunits of RNA polymerase II.

	_00313290	_00420200	_00152050	_01496790	_00227280	_00058670	_00144910	_00401830	_00818430	_00028580
_00313290	1									
_00420200	0.88	1								
_00152050	***0.94***	***0.98***	1							
_01496790	0.89	0.78	0.85	1						
_00227280	0.57	0.60	0.67	0.56	1					
_00058670	0.30	0.629	0.51	0.30	0.36	1				
_00144910	0.18	0.45	0.35	0.19	0.17	0.73	1			
_00401830	0.89	0.89	0.89	0.83	0.41	0.56	0.54	1		
_00818430	0.79	0.78	0.78	0.74	0.38	0.51	0.57	***0.94***	1	
_00028580	0.60	0.82	0.78	0.58	0.76	0.81	0.62	0.67	0.62	1
RPB1 a	−0.27	−0.24	−0.34	−0.32	−0.453	−0.33	−0.29	−0.29	−0.18	−0.41
RPB2 a	0.69	0.89	0.85	0.63	0.78	0.72	0.59	0.74	0.67	***0.97***
RPB3 a	***0.96***	***0.95***	***0.98***	0.87	0.71	0.46	0.27	0.88	0.78	0.76

a The gene IDs of RPB1, RPB2 and RPB3 are TTHERM_00047550, TTHERM_ 00077230 and 16.m05348, respectively.

The correlation coefficients greater than 0.9 are indicated in bold and italics.

Transcription factors E2F2 (TTHERM_00695710), E2F3 (TTHERM_01099150), Dp-1 (TTHERM_00107000) and Dp-2 (TTHERM_00047010, TTHERM_00016400) belong to the *E2F* transcription factor family, which plays a pivotal role in the regulation of cell proliferation and in anti-proliferative processes such as apoptosis and senescence. Correlation coefficient analyses indicate the expression of E2F2 (TTHERM_00695710) and Dp-2 (TTHERM_00047010) and of E2F3 (TTHERM_01099150) and Dp-2 (TTHERM_00016400) are highly correlated (Table 11), suggesting they may reside in distinct complexes that are involved in the regulation of transcription during early and late conjugation, respectively.

**Table 11:** Correlation coefficient between components of E2F family.

	_00047010	_00016400	_00107000	_01099150	_00695710	_01076950	_00721610
_00047010	1						
_00016400	0.06	1					
_00107000	0.48	0.29	1				
_01099150	−0.2	***0.80***	−0.20	1			
_00695710	***0.90***	−0.1	0.26	−0.30	1		
_01076950	0.66	0.58	0.44	0.16	0.40	1	
_00721610	0.48	0.22	0.73	−0.20	0.13	0.40	1

The correlation coefficients greater than 0.8 are indicated in bold and italics.

Myb family transcription factors play regulatory roles in developmental processes, such as cell division and chromosome partitioning. There are six conjugation-induced Myb family transcription factors. All except TTHERM_00131150, showed expression peaks at 4 or 6 hr, which suggests they may have functions in the control of the meiotic and postzygotic nuclear divisions.

Three UVH6-related transcription factors show different peak times of expression in early conjugation, as do 3 PHD finger- and 3 CPP-related factors. Two of three JMJ-related factors are co-expressed at 6 hr while a third one shows peak expression at 12 hr. Interestingly, two different TATA-binding proteins (TTHERM_00575350 and 00082170) are induced late in conjugation, probably in association with the development of the new MAC.

Although a detailed analysis of the genes regulated by specific transcripts is beyond the scope of the studies described here, we sought to determine whether co-expression analyses could identify candidate genes likely to be regulated by a particular TF. One gene, TTHERM_0044670, encoding a gene with weak homology to the CCAAT-binding transcription factor (Interpro ID: IPR003958) Hap5a/NF-Y [74], showed a particularly striking induction peaking at ∼4 hr of conjugation. Using this gene as the template to search for co-expressed genes, 141 genes were identified whose R values were >0.9 (Table S10), These included: 16 genes with DNA/RNA binding domains or associated with DNA damage/repair; 13 DNA polymerase, ligase, primase or helicase genes; 4 AAA family ATPases; 5 replication factor C genes; 9 genes involved in chromosome segregation, partitioning or minichromosome maintenance; 3 histone acetyltransferases or meythltransferases; *GTU1,* encoding gamma-tubulin; *CNA1*, which localizes to peripheral centromeres in micronuclei and *TAP45*, a component of telomerase holoenzyme. Thus, most of the genes that are co-expressed with this gene are ones that likely function in nuclei during conjugation. Preliminary analyses of partial knockout of this gene indicate that at least some of these co-transcribed genes are indeed regulated by NF-Y (Lifang Feng and WM, unpublished observations).

The above studies demonstrate that different types of TFs and even different members of the same TF family can show different, stage-specific patterns of expression. These observations suggest that most, if not all, of the large, stage-specific increases in transcription we have described that occur during conjugation are caused by highly regulated increases in specific transcription factors.

In addition to the expression of conjugation-specific genes from the parental macronucleus during conjugation, a number of specific, non-genic transcriptions are required for the RNAi-mediated process of DNA elimination. The current model for this process requires bi-directional transcription of both strands of sequences in the micronuclear genome early in conjugation to produce large non-genic, double-stranded RNAs that are cleaved by a dicer-like protein (Dcl1p) into 28nt scnRNAs. Based on the localization of subunit Rpb3p to the micronucleus at this time [75], this transcription is likely performed by RNA Polymerase II. Recent studies have also demonstrated extensive non-genic transcription from both the parental macronucleus that likely produces transcripts involved in the RNAi scanning process as well as transcription of IES sequences in the developing MAC to serve as targets for the scnRNAs in the later stages of elimination [26]. It will be of great interest to determine if any of the conjugation-induced transcription factors play a role in any of these IES-elimination-associated transcriptional processes during conjugation.

### Summary

We have established and validated the first platform for genome-wide microarray analysis of gene expression in a ciliated protozoan, using the eukaryotic model organism *T. thermophila*. These studies provide baseline data for expression of all predicted *Tetrahymena* open reading frames during growth, nutrient starvation and sexual conjugation. We have demonstrated that most of the ∼27,000 open reading frames are transcribed at one or more of the physiological/developmental stages of the *Tetrahymena* life cycle and shown that all stages, especially the sexual process of conjugation, are characterized by dramatic changes in patterns of gene expression. Our analyses have demonstrated that co-expression of genes during conjugation can identify proteins that participate in the same process and have identified a number of candidate genes likely to function during distinct stages of the genome reorganization that accompanies differentiation of the somatic macronucleus from the germline micronucleus. We have also identified an unexpectedly large number of genes whose specific expression in late conjugation indicates that, in addition to DNA elimination, chromosome fragmentation and rDNA amplification, a number of heretofore unrecognized developmental processes occur specifically during late conjugation.

## Materials and Methods

### Strains and culture conditions

Wild-type cell lines B2086 and CU428 of *Tetrahymena thermophila* were provided by Dr. P.J. Bruns, Cornell University, Ithaca, NY, (now available through the National *Tetrahymena* Stock Center, http://tetrahymena.vet.cornell.edu/index.html). Both of these cell lines have inbred strain B genetic background, as does cell line SB210, the source of the MAC genome sequence used to design the microarray probes. Cells were grown in SPP medium [76] at 30°C. For microarray analyses of growing cells, we studied CU428 cells at three different densities that, for convenience are referred to as low (L-l), medium (L-m) and high (L-h). These correspond respectively to ∼1×10^5^ cells/ml, in which, under our culture conditions cells are in true logarithmic growth; ∼3.5×10^5^ cells/ml which corresponds to cells in the decelleratory stage of culture growth and ∼1×10^6^ cells/ml, which corresponds to cells nearly in stationary phase, before appreciable cell death has occurred. For starvation, CU428 cells at ∼2×10^5^ cells/ml were collected, washed and starved at 2×10^5^ cells/ml in 10 mM Tris (pH 7.5); samples were collected at0, 3, 6, 9, 12, 15 and 24 hours(referred to as S-0, S-3, S-6, S-9, S-12, S-15 and S-24). For conjugation, equal volumes of B2086 and CU428 cells that had been starved for 18 hours in 10 mM Tris (pH 7.5) at 2×10^5^ cells/ml, were mixed, and samples were collected at 0, 2, 4, 6, 8, 10, 12, 14, 16 and 18 hours after mixing (referred to as C-0, C-2, C-4, C-6, C-8, C-10, C-12, C-14, C-16 and C-18). The overall similarities in gene expression (see Results and Discussion), and the levels of H1 phosphorylation [77] and the cytological stages [17] were used to assay the repeatability of independent preparations of starvation and conjugation samples, respectively.

### Isolation of Total RNA

Qiashredder spin columns were used for homogenization followed by total RNA extraction using the RNeasy Protect Cell Mini Kit (Qiagen, Valenica, CA) according to manufacturer's instructions. The concentration of total RNA was determined using a NanoDrop ND-1000 spectrophotometer (NanoDrop Technologies, Rockland, DE) and RNA integrity was verified using a Bioanalyzer 1000 (Agilent, Palo Alto, CA).

### Sample labeling

cDNA synthesis and Cy 3 labeling was performed by Roche NimbleGen Systems, Inc. as described previously [78]. Briefly, equal amounts of total RNA for each sample were converted to double-stranded cDNA using the SuperScript II cDNA Conversion Kit (Invitrogen, Carlsbad, CA). Because this method uses an oligo dT primer, RNAs lacking polyA tails are likely to be under-represented.

### Genome data and probe design

A total of 28,064 *T. thermophila* sequences from cell line SB210 (inbred strain B genetic background) obtained at The Institute of Genome Research (TIGR; now known as the J. Craig Venter Institute; http://www.tigr.org/tdb/e2k1/ttg/) including 27,055 predicted protein-coding genes, non-protein-coding RNA and tRNA genes were used to construct high-density *T. thermophila* genome-wide oligonucleotide DNA microarrays. Only results from the putative protein coding genes are discussed here. For each of the sequences, 13 or 14 unique 60-mer oligonucleotide probes were designed by Roche NimbleGen Systems using a multi-step approach to select probes with optimal predicted hybridization characteristics. To the extent possible, probes were evenly distributed over the length of gene models, although efforts to maximize mismatches among closely related genes resulted in some probe clustering. All probes were designed as “perfect match” oligonucleotides (oligos).

Since the Tetrahymena macronuclear genome sequence became available, three versions of Tetrahymena gene annotation were released, in 2005, 2006 and 2008, respectively. The TGD (Tetrahymena Genome Database) website (http://www.ciliate.org/) uses the 2005 version, which designates every predicted gene with the TTHERM_XXXXXX gene ID. In the slightly improved 2006 gene predictions, some genes and their 2005 IDs were changed. For example, one predicted gene in the 2005 version (TTHERM_00299870) was separated into two predicted genes, one retaining the old ID (TTHERM_00299870) while the other one was given a new ID (eg, 3691.m01119, for *WAG1* in Table 5), lacking a TTHERM designation. Our microarray was designed based on the 2006 gene annotations, before the 2008 version was available. Thus, while most of the genes we have studied have a TTHERM designation that can be found in TGD, some do not. For genes lacking a TTHERM_ID in the microarray results, the gene sequence can be retrieved by downloading and searching the cDNA sequence file (TTA1_08302006.cdna) or protein sequence file (TTA1_08302006.pep) from ftp://ftp.tigr.org/pub/data/Eukaryotic_Projects/t_thermophila/annotation_dbs/interim_annotation_release_08313006/. In the recent 2008 version (ftp://ftp.tigr.org/pub/data/Eukaryotic_Projects/t_thermophila/annotation_dbs/final_release_oct2008/), all genes ID without a TTHERM_ ID in the 2006 version have been designated with a new and unique TTHERM_ ID (e.g. *WAG1* with the 2006 ID of 3691.m01119 is now 3691.m01119 TTHERM_00299879). Thus, while most of the 2006 gene designations used in the microarray correspond to the 2005 designations and can be retrieved from TGD, gene IDs unique to the 2006 or 2008 versions cannot be used to search the TGD website. However, the gene sequences retrieved from any of the databases can be used to find the chromosomal coordinates in TGD.

### Microarray synthesis, hybridization, and staining

The custom *T. thermophila* genome-wide oligonucleotide DNA microarrays were manufactured by Roche NimbleGen Systems, Inc. using the maskless photolithography method described previously [79], [80]. Each oligo synthesized represented a 16 μm×16 μm feature on the hybridization surface of the microarray; there were 385,000 features within a 17.4 mm×13 mm array area. Hybridization, staining, and processing of arrays were performed by Roche NimbleGen Systems as previously described [81].

For each growing and starved *Tetrahymena* sample, hybridizations were performed on three independent microarrays (e.g. L1, L2 and L3; S1, S2 and S3). For analysis of conjugation, hybridizations were performed on two independent microarrays (e.g. C1 and C2). Except where methodological reproducibility was analyzed, each individual microarray represents a separate experiment; total RNA was isolated from independent cell cultures, then independently converted to cDNA, labeled and hybridized.

### Data extraction and analysis

Arrays were scanned by Roche NimbleGen using a GenePix 4000B microarray scanner (Molecular Devices, Sunnyvale, CA) and the data were extracted using NimbleScan software. Array normalization was performed using the quantile normalization method [82]. Normalized expression values for the individual probes were used to obtain the expression values for a given open reading frame (ORF) by using the robust multiarray average (RMA) procedure as previously described by Irizarry et al [39]. Finally, the data were analyzed based on the RMA-processed expression values (RMA calls).

### Basic analyses

The *r* ^2^, fold changes, *p* values and heat maps were calculated using ArrayStar software, version 2.0 (DNASTAR, Inc, Madison, WI). In order to identify putative genes with significant expression changes, an F-test (ANOVA) corrected for multiple testing by the False Discovery Rate (FDR) method was performed for each experiment. Putative genes for which the *p* value was less than 0.05 were considered as differentially expressed.

Gene annotations in the tables were based on an initial search of TGD (http://www.ciliate.org/genomedata.shtml) with the gene ID indicated by the Roche-NimbleGen array design. If a hit to a gene of known function was obtained, the annotation was used (E value is “0.0”). If a hit to a hypothetical protein (ORF) was obtained, the predicted protein sequence was retrieved and used in a Blastp search of the non-redundant proteins in the NCBI database (http://www.ncbi.nlm.nih.gov/BLAST/). Putative proteins lacking significant similarity to any known protein were listed only as a “Predicted *Tetrahymena* ORF” (E value as “-”). ORFs having similarity to a protein encoded by a related *Tetrahymena* gene or to a protein encoded in another organism were indicated as such. In cases with similarity either to a putative *Tetraymena* protein or to one in another species, the sequence with the lower e value was listed.

To search for non-transcribed gene models, we used a program of our own design, compiled with the Intel Visual Fortran Compiler, version 6.5 (Compaq Computer Corporation, Houston, TX). It was designed to calculate the maximum and minimum expression values of putative ORFs in the growth (Max_L and Min_L), starvation (Max_S and Min_S) and conjugation (Max_C and Min_C) samples respectively.

### Background subtraction

Background levels of hybridization were determined by including, as negative controls, 4308 randomly generated sequence oligo probes that did not correspond to any *Tetrahymena* genome sequence but were of comparable length and GC content to the experimental probes on each array. These probes served as a measure of non-specific, “methodological” background binding, i.e., due to preparation of samples, array manufacture and processing. Once this “methodological background” was measured, we determined how many multiples of this background should be subtracted in order to correct for the additional background due to weak hybridization of fortuitously similar sequence to the *Tetrahymena* probes, according to the approach described in Wei [38]. A family of curves was obtained by subtracting, from the signal intensity of every probe, increasing multiples of the negative control signal intensity. The level of subtraction at which the distribution converged to a robust profile, which retains no hint of the very large peak of non-specific hybridization observed in the unsubtracted distribution, was determined from the graph. The resulting value was then subtracted from every probe in every sample.

### Identifying co-expressed genes

Genes with coordinate expression patterns during conjugation are candidates for participating in the same developmental process. Groups of genes co-expressed with each of several genes with different experimentally determined function were identified based on Pearson correlation coefficients (R*)*, derived by comparing their patterns of expression using all data from growing, starved and conjugating cells. R values were calculated using Excel_Tool_Data Analysis_correlation coefficient. Correlation coefficients with a value of more than 0.9 were considered indicative of co-expression.

### Estimating number of genes that are false positives for gene expression

Estimates of the genome-wide fraction of expressed genes can be inflated by false positives, i.e., genes that, while not expressed, show above background signal intensity because their probes cross-hybridize with the transcript of a closely related gene. To estimate the fraction of false positives, we examined families of recently duplicated genes in the *T. thermophila* genome (Table S10 in [35]), and identified expressed genes within each family. We analyzed a sample of 474 genes belonging to every one of the 16 families containing 27–100 members, regardless of whether the family is functionally annotated or includes only “hypothetical” genes. We then clustered co-expressed genes within each family, on the assumption that a non-expressed gene showing cross-hybridization with another gene should show highly correlated expression (R>0.9) with it. We base this threshold on the idea that binding energies between a given probe and transcript should be constant from array to array and condition to condition, subject only to methodological variation. Such variation was low in these arrays, with R values>0.95 (see Results and Discussion section). Thus R>0.90 seems to be a conservatively low threshold for apparent co-expression due to cross-hybridization. Genes were included in a cluster if they show co-expression with at least one other member of the cluster.

We then estimated the maximum fraction of false positives by further assuming that only genes showing a probe-transcript sequence identity greater than at least 70% (at most 18/60 mismatches) could show cross-hybridization above background, based on the findings of Hughes *et al.* [40] The probe that made the highest contribution to the gene signal intensity was used for the sequence identity measurement. For every co-expressed gene pair, the maximum value of the reciprocal probe-transcript sequence identity was used. When a group of *n* genes showed both co-expression and >70% probe-transcript sequence identity in at least some pairwise comparisons, it was assumed that at least one gene in the group had to be a truly expressed gene and *n-1* was the conservative false positive count used for this cluster. The final fraction of false positives was multiplied by 0.4, to correct for the genome-wide percentage of genes in families (of at least two members), which is 40%. As indicated in the foregoing, and further discussed under Results and Discussion, these assumptions likely overestimate the fraction of false positives.

### Accession Numbers

Microarray data have been deposited with the NCBI Gene Expression Omnibus (http://www.ncbi.nlm.nih.gov/geo) under accession numbers in Table S11.

## Supporting Information

Figure S1 The expression pattern search program compiled by Intel Visual Fortran Compiler, version 6.5.
(0.02 MB PDF)

Table S1 Comparison of variation between the conjugation replicates done in two different laboratories (WM and RP).
(0.07 MB DOC)

Table S2 Multigene families containing candidate non-transcribed genes.
(0.05 MB DOC)

Table S3 Ninety-five constitutively expressed genes whose signal intensities were >250× corrected background.
(0.12 MB DOC)

Table S4 Global analyses of gene expression at specific stages.
(0.03 MB DOC)

Table S5 S5-1.Number of Growth/Starvation/Conjugation-specific genes. S5-2. Number of Growth/Starvation/Conjugation-upregulated genes.
(0.06 MB DOC)

Table S6 Ninety-one Growth-specific genes expressed at levels higher than 2× corrected background.
(0.13 MB DOC)

Table S7 Ninety starvation-specific genes expressed at levels higher than 2× corrected background.
(0.12 MB DOC)

Table S8 Genes co-expressed with putative transposase, TTHERM_01107220.
(0.12 MB DOC)

Table S9 Fifty-one Conjugation-induced/specific transcription factors of Tetrahymena thermophila.
(0.09 MB DOC)

Table S10 NF-Y (TTHERM_00439030) co-expressed genes.
(0.17 MB DOC)

Table S11 The accession numbers of microarray data in this paper as submitted to the NCBI Gene Expression Omnibus.
(0.05 MB DOC)
