# Supplementary material for: Microarray Analyses of Gene Expression during the Tetrahymena thermophila Life Cycle
Source: PLoS One. 2009 Feb 10;4(2):e4429. doi: 10.1371/journal.pone.0004429 (PMC2636879; doi:10.1371/journal.pone.0004429)
Supplement: Figure S1 — The expression pattern search program compiled by Intel Visual Fortran Compiler, version 6.5. (0.02 MB PDF) [file pone.0004429.s001.pdf]

Figure S1. The expression pattern search program compiled by Intel Visual Fortran Compiler, version 6.5.

```
PROGRAM search_gene
   implicit none
   type :: t_data_struct
      Character *30        :: SEQ_ID
      DOUBLE PRECISION,dimension(20) :: timedata
      Character *30      :: SECONDARY_NAME
      Character *30      :: SECONDARY_NAME2
   END type t_data_struct

   type (t_data_struct) :: row_data
   type (t_data_struct),dimension(200) :: buffer
   character *30 :: datafilename
   character *50 :: outputname
   CHARACTER (LEN=30), DIMENSION(22) :: temp
   double precision :: max_firstpart
   double precision :: max_L,max_s,max_c,min_L,min_s,min_c
   integer :: i,idnum,ios,ic,k
   ! read datafilename
      write (*,*) "Please input your data file name:"
      read *, datafilename
      open(UNIT=20, FILE=trim(datafilename), STATUS="OLD", IOSTAT=ios)
      if (ios>0) then
            print *, "Cannot find that file!"
            stop
      else
            outputname = trim(datafilename)//"_stf.txt"
            open(UNIT=30, FILE=trim(outputname), STATUS="REPLACE", IOSTAT=ios)

            open(UNIT=50, FILE="LOG.txt",STATUS="REPLACE", IOSTAT=ios)
      endif
   ! load data row by row
   read(UNIT=20, FMT=*) temp
   !write (*,*) temp
   !write(UNIT=30,FMT='(22(a30,1X))') temp

   i=1
   idnum=0
   ic=0
   ios=0
   do
```

```fortran
read (20,FMT=*,IOSTAT=ios) row_data
!write(*,FMT='(3(a30),4(f10.5),a100)') row_data
if (ios/=0) then

        write(*,*) "end of file"
        write(*,*) "total data set:",idnum
        write(*,*) "NO. satisfying conditions:", ic
        write(*,*) "NO. regular:", idnum-ic

        write(50,*) "end of file"
        write(50,*) "total data set:",idnum
        write(50,*) "NO. satisfying conditions:", ic
        write(50,*) "NO. regular:", idnum-ic

        close(20)
        close(30)
        close(50)
        exit
endif
idnum=idnum+1
max_firstpart=0d0
! calculate max and min for L part
max_L=row_data%timedata(1)
min_L=row_data%timedata(1)
do i=1,3
      if (row_data%timedata(i)>max_L) then
            max_L=row_data%timedata(i)
      elseif (row_data%timedata(i)<min_L) then
            min_L=row_data%timedata(i)
      endif
enddo
! calculate max and min for s part
max_s=row_data%timedata(4)
min_s=row_data%timedata(4)
do i=4,10
      if (row_data%timedata(i)>max_s) then
            max_s=row_data%timedata(i)
      elseif (row_data%timedata(i)<min_s) then
            min_s=row_data%timedata(i)
      endif
enddo
! calculate max and min for c part
max_c=row_data%timedata(11)
```

```
        min_c=row_data%timedata(11)
        do i=11,20
            if (row_data%timedata(i)>max_c) then
                max_c=row_data%timedata(i)
            elseif (row_data%timedata(i)<min_c) then
                min_c=row_data%timedata(i)
            endif
        enddo
        ! now specify conditions
         if (max_L<99.and.max_s<99.and.max_c<99) then
                                        ic=ic+1
                        write (30,'(A30,1X,20(F20.5,1X),A30,1X,A30)') row_data
                                endif

    enddo
    pause
END Program search_gene
```
