# Supplementary material for: Microarray Analyses of Gene Expression during the Tetrahymena thermophila Life Cycle
Source: PLoS One. 2009 Feb 10;4(2):e4429. doi: 10.1371/journal.pone.0004429 (PMC2636879; doi:10.1371/journal.pone.0004429)
Supplement: Table S1 — Comparison of variation between the conjugation replicates done in two different laboratories (WM and RP). (0.07 MB DOC) [file pone.0004429.s002.doc]

**Table S1. Comparison of variation between the conjugation replicates done in two different laboratories (WM and RP).**

|  |  | RP | | | | | | | | | | |
| --- | --- | --- | --- | --- | --- | --- | --- | --- | --- | --- | --- | --- |
|  | Samples | 0h | 15min | 2h | 4h | 6h | 8h | 10h | 12h | 14h | 16h | 18h |
|  | 0h-1 | 0.92 | 0.88 | 0.79 | 0.68 | 0.7 | 0.73 | 0.79 | 0.83 | 0.82 | 0.83 | 0.84 |
|  | 2h-1 | 0.7 | 0.73 | 0.9 | 0.87 | 0.78 | 0.75 | 0.74 | 0.78 | 0.82 | 0.77 | 0.75 |
|  | 4h-1 | 0.69 | 0.7 | 0.79 | 0.89 | 0.89 | 0.79 | 0.75 | 0.77 | 0.81 | 0.82 | 0.79 |
|  | 6h-1 | 0.67 | 0.68 | 0.74 | 0.8 | 0.92 | 0.9 | 0.81 | 0.79 | 0.79 | 0.81 | 0.83 |
|  | 8h-1 | 0.69 | 0.69 | 0.72 | 0.68 | 0.79 | 0.89 | 0.88 | 0.86 | 0.84 | 0.82 | 0.86 |
|  | 10h-1 | 0.74 | 0.75 | 0.78 | 0.69 | 0.76 | 0.85 | 0.91 | 0.92 | 0.88 | 0.85 | 0.86 |
|  | 12h-1 | 0.73 | 0.77 | 0.8 | 0.72 | 0.77 | 0.86 | 0.9 | 0.94 | 0.95 | 0.87 | 0.87 |
|  | 14h-1 | 0.77 | 0.78 | 0.81 | 0.75 | 0.79 | 0.83 | 0.88 | 0.91 | 0.93 | 0.91 | 0.9 |
|  | 16h-1 | 0.8 | 0.79 | 0.8 | 0.73 | 0.79 | 0.84 | 0.88 | 0.9 | 0.91 | 0.92 | 0.94 |
| WM | 18h-1 | 0.81 | 0.79 | 0.77 | 0.68 | 0.74 | 0.82 | 0.86 | 0.89 | 0.89 | 0.89 | 0.92 |
|  | 0h-2 | 0.87 | 0.85 | 0.78 | 0.66 | 0.65 | 0.67 | 0.73 | 0.78 | 0.77 | 0.76 | 0.78 |
|  | 2h-2 | 0.72 | 0.75 | 0.91 | 0.85 | 0.77 | 0.74 | 0.74 | 0.78 | 0.82 | 0.77 | 0.76 |
|  | 4h-2 | 0.71 | 0.73 | 0.84 | 0.9 | 0.87 | 0.78 | 0.76 | 0.78 | 0.81 | 0.8 | 0.79 |
|  | 6h-2 | 0.66 | 0.69 | 0.77 | 0.83 | 0.9 | 0.86 | 0.77 | 0.77 | 0.78 | 0.78 | 0.79 |
|  | 8h-2 | 0.69 | 0.7 | 0.76 | 0.75 | 0.83 | 0.9 | 0.87 | 0.85 | 0.83 | 0.81 | 0.84 |
|  | 10h-2 | 0.72 | 0.74 | 0.78 | 0.72 | 0.78 | 0.88 | 0.9 | 0.91 | 0.88 | 0.83 | 0.85 |
|  | 12h-2 | 0.73 | 0.76 | 0.79 | 0.7 | 0.75 | 0.84 | 0.89 | 0.92 | 0.91 | 0.85 | 0.85 |
|  | 14h-2 | 0.77 | 0.78 | 0.81 | 0.73 | 0.76 | 0.83 | 0.89 | 0.91 | 0.93 | 0.89 | 0.88 |
|  | 16h-2 | 0.81 | 0.81 | 0.82 | 0.73 | 0.76 | 0.82 | 0.87 | 0.9 | 0.91 | 0.9 | 0.91 |
|  | 18h-2 | 0.81 | 0.8 | 0.8 | 0.7 | 0.73 | 0.8 | 0.86 | 0.89 | 0.89 | 0.88 | 0.9 |

Numbers represent r^2^ values. Highest r^2^ values are highlighted.
