# Supplementary material for: Microarray Analyses of Gene Expression during the Tetrahymena thermophila Life Cycle
Source: PLoS One. 2009 Feb 10;4(2):e4429. doi: 10.1371/journal.pone.0004429 (PMC2636879; doi:10.1371/journal.pone.0004429)
Supplement: Table S2 — Multigene families containing candidate non-transcribed genes. (0.05 MB DOC) [file pone.0004429.s003.doc]

**Table S2. Multigene families containing candidate non-transcribed genes.**

| **Gene description (multigene family)** | **Number of genes** |
| --- | --- |
| ABC transporter family protein | 72 |
| AMP-binding enzyme family protein | 112 |
| ATPase | 41 |
| BdrK | 10 |
| Bowman-Birk serine protease inhibitor family protein | 25 |
| Calpain family cysteine protease containing protein | 22 |
| Cation channel family protein | 82 |
| Chlamydial polymorphic outer membrane protein repeat containing protein | 70 |
| Cyclic nucleotide-binding domain containing protein | 151 |
| Cytochrome P450 family protein | 11 |
| Dual specificity phosphatase, catalytic domain containing protein | 20 |
| EF hand family protein | 9 |
| EGF-like domain containing protein | 19 |
| Eukaryotic aspartyl protease family protein | 21 |
| Glutathione S-transferase, N-terminal domain containing protein | 24 |
| Helicase | 11 |
| Insect antifreeze protein | 45 |
| Ku70/Ku80 beta-barrel domain containing protein | 16 |
| Leishmanolysin family protein | 16 |
| Leucine Rich Repeat family protein | 80 |
| Major Facilitator Superfamily protein | 23 |
| Neurohypophysial hormones, N-terminal Domain containing protein | 84 |
| Oxidoreductase | 16 |
| Papain family cysteine protease containing protein | 20 |
| Phosphatidylserine decarboxylase family protein | 15 |
| Protein kinase domain containing protein | 212 |
| Rab1/ *RAB2E/ RAB42/ RAB43/* Ras | 13 |
| Regulator of chromosome condensation (RCC1) | 9 |
| REJ domain containing protein | 33 |
| Sec1 family protein | 30 |
| Serine protease | 9 |
| SF-assemblin/beta giardin family protein | 16 |
| small GTP-binding protein domain containing protein | 26 |
| Surface protein with furin-like cysteine repeats | 25 |
| Tlr 1Fp/2Fp4Rp/5Rp/5Fp/6Rp/6Fp/7Rp protein | 66 |
| TPR Domain containing protein | 129 |
| Transposase family protein | 15 |
| Ubiquitin family protein | 33 |
| Ubiquitin-conjugating enzyme family protein | 24 |
| von Willebrand factor type A domain containing protein | 10 |
| YLP motif family protein | 11 |
| zinc finger family protein | 73 |
