# Supplementary material for: Microarray Analyses of Gene Expression during the Tetrahymena thermophila Life Cycle
Source: PLoS One. 2009 Feb 10;4(2):e4429. doi: 10.1371/journal.pone.0004429 (PMC2636879; doi:10.1371/journal.pone.0004429)
Supplement: Table S3 — Ninety-five constitutively expressed genes whose signal intensities were >250× corrected background. (0.12 MB DOC) [file pone.0004429.s004.doc]

**Table S3. Ninety-five constitutively expressed genes whose signal intensities were > 250X corrected background.**

| **Gene ID** | **Gene annotation^*^** | **E value** |
| --- | --- | --- |
| TTHERM_02358030 | Unnamed protein product [Vitis vinifera] | 0.029 |
| TTHERM_00471090 | 40S ribosomal protein S7-like [Ixodes scapularis] | 3e-20 |
| TTHERM_00636970 | 60s Acidic ribosomal protein | 5e-147 |
| TTHERM_00943010 | 60S ribosomal protein L31, putative | 1e-59 |
| TTHERM_00365340 | AAA family ATPase, CDC48 subfamily protein | 0 |
| TTHERM_00729250 | Aldehyde dehydrogenase (NAD) family protein | 0 |
| TTHERM_00630470 | *ARP1* | 0 |
| TTHERM_00585260 | ATP synthase beta chain | 0 |
| TTHERM_00571860 | ATP synthase F1, alpha subunit family protein | 0 |
| TTHERM_00558620 | *ATU1* | 0 |
| TTHERM_00836580 | *BTU2* | 0 |
| TTHERM_00630500 | *CAM1* | 1e-47 |
| TTHERM_02625250 | CARD15-like protein, putative | 6e-10 |
| TTHERM_01472440 | CARD15-like protein, putative | 3e-08 |
| TTHERM_01623860 | CARD15-like protein, putative | 8e-08 |
| TTHERM_01146030 | *CAT1* | 0 |
| TTHERM_00329720 | CHCH domain containing | 1e-147 |
| TETRA00S0001536 | Cytochrome P450 like_TBP [Citrullus lanatus] | 5e-09 |
| TTHERM_00938820 | *EEF2* | 0. |
| TTHERM_00128320 | Eukaryotic aspartyl protease family protein | 0 |
| TTHERM_00434000 | Eukaryotic aspartyl protease family | 2e-11 |
| TTHERM_00216010 | *FTT18* | 5e-141 |
| TTHERM_00160770 | *FTT49* | 6e-77 |
| TTHERM_00056140 | Glutaminyl-tRNA synthetase family protein | 0 |
| TTHERM_00378890 | *GRL5* | 0 |
| TTHERM_01055600 | *GRL8* | 0 |
| TTHERM_00016170 | *HHT3* | 7e-73 |
| TTHERM_00016200 | *HHT4* | 2e-72 |
| TTHERM_00105110 | *HSP70A* | 0 |
| TTHERM_00158520 | *HSP82* | 0 |
| TTHERM_00735340 | Inorganic H+ pyrophosphatase | 0 |
| TTHERM_00218790 | Inorganic pyrophosphatase | 0 |
| TTHERM_00491130 | J immobilization antigen, putative | 1e-20 |
| TTHERM_01151500 | KH domain containing protein | 1e-139 |
| TTHERM_00577240 | L1P family of ribosomal proteins | 4e-122 |
| TTHERM_01773710 | Leucine Rich Repeat family protein | 4e-08 |
| TTHERM_00439150 | Predicted Tetrahymena ORF ^a^ | － |
| TTHERM_00193160 | Predicted Tetrahymena ORF ^a^ | － |
| TTHERM_01363600 | Predicted Tetrahymena ORF ^a^ | － |
| TTHERM_02649280 | Predicted Tetrahymena ORF ^a^ | － |
| TTHERM_01327880 | Predicted Tetrahymena ORF ^a^ | － |
| TTHERM_00426260 | Predicted Tetrahymena ORF ^a^ | － |
| TTHERM_00760310 | Papain family cysteine protease | 0 |
| TTHERM_00079450 | Papain family cysteine protease containing protein | 0 |
| TTHERM_00628610 | Papain family cysteine protease containing protein | 0 |
| TTHERM_00641150 | Papain family cysteine protease containing protein | 0 |
| TTHERM_00161130 | Papain family cysteine protease containing protein | 0 |
| TTHERM_00590130 | *PHP2* | 6e-112 |
| 1126.m00019 ^b^ | PMCA19 [Paramecium tetraurelia] | 2.1 |
| TTHERM_01339590 | PREDICTED: similar to zinc finger protein 609 [Canis familiaris] | 0.40 |
| TTHERM_00795690 | Protein kinase domain containing protein | 0.027 |
| TTHERM_00220990 | Protein kinase domain containing protein. | 0 |
| TTHERM_00686220 | Putative aminotransferase [Nocardia farcinica IFM 10152 | 1.4 |
| TTHERM_01339580 | Putative ankyrin repeat protein [Dictyostelium discoideum AX4] | 1.6 |
| TTHERM_00442200 | Putative GroEL-like chaperonine protein [Pseudomonas phage EL] | 2.4 |
| TTHERM_00316280 | *RAB1A* | 3e-114 |
| TTHERM_01113100 | Receptor of activated protein kinase C 1A, component of 40S small  ribosomal subunit [Ostreococcus lucimarinus CCE9901] | 5e-76 |
| TTHERM_00149300 | Ribosomal family S4e containing protein | 0 |
| TTHERM_00476670 | Ribosomal L18ae protein family protein | 7e-112 |
| TTHERM_00736480 | Ribosomal L18p/L5e family protein | 0 |
| TTHERM_00339620 | Ribosomal L40e family | 7e-95 |
| TTHERM_01053000 | Ribosomal protein L10.e containing | 8e-123 |
| TTHERM_00028740 | Ribosomal protein L11, RNA binding domain containing protein | 4e-81 |
| TTHERM_00578640 | Ribosomal protein L13e containing protein | 1e-114 |
| TTHERM_01289110 | Ribosomal protein L15 | 1e-113 |
| TTHERM_00476640 | Ribosomal protein L24e containing | 2e-83 |
| TTHERM_00085180 | Ribosomal protein L32 containing protein | 6e-72 |
| TTHERM_00075670 | Ribosomal protein L37Ae [Timarcha balearica] | 3e-29 |
| TTHERM_00812780 | Ribosomal protein L38e [Plasmodium falciparum 3D7] | 4e-05 |
| TTHERM_00333210 | Ribosomal protein L4/L1 family protein | 0 |
| TTHERM_00773340 | Ribosomal protein L7Ae containing | 6e-162 |
| TTHERM_00522630 | Ribosomal protein S2 containing protein | 2e-97 |
| TTHERM_00686150 | Ribosomal protein S27a containing | 1e-103 |
| TTHERM_00434070 | Ribosomal protein S4 containing protein | 9e-84 |
| TTHERM_00193740 | Ribosomal protein S5 containing | 2e-161 |
| TETRA00S0016288 | Ribosomal protein S6A (S10A) (rp9) (YS4) [Pichia stipitis CBS6054] | 4e-69 |
| TTHERM_00716240 | Ribosomal Proteins L2, C-terminal domain containing protein | 7e-126 |
| TTHERM_00047480 | Ribosomal S3Ae family protein | 3e-118 |
| TTHERM_00488340 | *RPL29* | 7e-51 |
| TTHERM_00497880 | *RPL3* | 0 |
| TTHERM_00189080 | Secretory pathway GDP dissociation inhibitor, putative | 0 |
| TTHERM_02141640 | Senescence-associated protein [Brugia malayi] | 8e-50 |
| TTHERM_00606960 | *SerH* | 0 |
| TTHERM_00655820 | Translation elongation factor EF-1, subunit alpha. | 0 |
| TTHERM_00227770 | Translation initiation factor eIF-5A family protein | 2e-120 |
| 5.m05449 ^b^ | Transporter 43 [Natronomonaspharaonis DSM 2160] | 0.85 |
| TTHERM_00077240 | *TTU3* | 0 |
| TTHERM_01513230 | Tubulin-tyrosine ligase family protein | 2e-08 |
| TTHERM_01853030 | Tubulin-tyrosine ligase family | 2e-08 |
| TTHERM_01748600 | Tubulin-tyrosine ligase family protein | 2e-08 |
| TTHERM_01100290 | Tubulin-tyrosine ligase family | 2e-07 |
| TTHERM_01184200 | Tubulin-tyrosine ligase family protein | 3e-07 |
| TTHERM_00531900 | Ubiquitin-conjugating enzyme family | 1e-100 |
| TTHERM_00535320 | Unnamed protein product [Tetraodon nigroviridis] | 0.033 |
| TTHERM_00653620 | V-type ATPase, C subunit family protein | 1e-77 |

**^*^** Similarity is to a *Tetrahymena thermophila* gene unless another species is indicated.

Footnotes a and b are as in Table 5.
