# Supplementary material for: Microarray Analyses of Gene Expression during the Tetrahymena thermophila Life Cycle
Source: PLoS One. 2009 Feb 10;4(2):e4429. doi: 10.1371/journal.pone.0004429 (PMC2636879; doi:10.1371/journal.pone.0004429)
Supplement: Table S4 — Global analyses of gene expression at specific stages. (0.03 MB DOC) [file pone.0004429.s005.doc]

**Table S4. Global analyses of gene expression at specific stages.**

| **Search Conditions^*^** | **Number of Genes** |
| --- | --- |
| **Not expressed during both growth and starvation**  Max_L < 99 and Max_S < 99 | 8039 |
| **Expressed during both growth and starvation**  Max_L > 99 and Max_S > 99 | 16120 |
| **Expressed during growth but not starvation**  Max_L > 99 and Max_S < 99 | 678 |
| **Expressed during starvation but not growth**  Max_L < 99 and Max_S > 99 | 2227 |
| **Expressed during starvation and conjugation**  Max_L < 99; Max_C > 99 and Max_S > 99 (S-3 > 99) | 1866 (1118) |
| **Expressed only during conjugation**  Max_L < 99 and Max_S < 99; Max_C > 99 | 2153 |

* Max_L, Max_S and Max_C were the maximum signal intensity during Log growth, Starvation and Conjugation respectively.
