# Supplementary material for: Microarray Analyses of Gene Expression during the Tetrahymena thermophila Life Cycle
Source: PLoS One. 2009 Feb 10;4(2):e4429. doi: 10.1371/journal.pone.0004429 (PMC2636879; doi:10.1371/journal.pone.0004429)
Supplement: Table S5 — S5-1.Number of Growth/Starvation/Conjugation-specific genes. S5-2. Number of Growth/Starvation/Conjugation-upregulated genes. (0.06 MB DOC) [file pone.0004429.s006.doc]

**Table S5-1. Number of Growth/Starvation/Conjugation-specific genes.**

|  | **Search conditions^*^** | **Number of Genes** |
| --- | --- | --- |
| **Growth**  **-specific** | Max_S <99 and Max_C < 99  Max_L > 99 | 206 |
|  | Max_S <99 and Max_C < 99  Max_L >2×99 | 91 |
|  | Max_S < 99 and Max_C < 99  Max_L > 5×99 | 31 |
|  | Max_S <99 and Max_C < 99  Max_L >10×99 | 13 |
|  | Max_S <99 and Max_C < 99  Max_L > 50×99 | 0 |
|  | Max_S < 99 and Max_C < 99  Max_L >100×99 | 0 |
| **Starvation -specific** | Max_L < 99 and Max_C < 99  Max_S > 99 | 361 |
|  | Max_L < 99 and Max_C < 99  Max_S >2×99 | 90 |
|  | Max_L < 99 and Max_C < 99  Max_S > 5×99 | 13 |
|  | Max_L < 99 and Max_C < 99  Max_S>10×99 | 5 |
|  | Max_L < 99 and Max_C < 99  Max_S>50×99 | 0 |
|  | Max_L < 99 and Max_C < 99  Max_S >100×99 | 0 |
| **Conjugation**  **-specific** | Max_L < 99 and Max_S < 99  Max_C >99 | 2153 |
|  | Max_L < 99 and Max_S < 99  Max_C>2×99 | 1068 |
|  | Max_L < 99 and Max_S < 99  Max_C > 5×99 | 503 |
|  | Max_L < 99 and Max_S < 99  Max_C > 10×99 | 331 |
|  | Max_L < 99 and Max_S < 99  Max_C > 50×99 | 146 |
|  | Max_L < 99 and Max_S < 99  Max_C> 100×99 | 94 |

* Max_L, Max_S and Max_C were the maximum signal intensity during Log growth, Starvation and Conjugation respectively.

**Table S5-2. Number of Growth/Starvation/Conjugation-upregulated genes.**

|  | **Search conditions*** | **Number of Genes** |
| --- | --- | --- |
| **Growth -upregulated genes** | Max_S > 99 and Max_C > 99  Max_L > 2×Max_S and Max_L > 2×Max_C | 1049 |
|  | Max_S > 99 and Max_C > 99  Max_L > 5×Max_S and Max_L > 5×Max_C | 155 |
|  | Max_S > 99 and Max_C > 99  Max_L >10×Max_S and Max_L > 10×Max_C | 46 |
|  | Max_S > 99 and Max_C > 99  Max_L > 50×Max_S and Max_L > 50×Max_C | 0 |
|  | Max_S > 99 and Max_C > 99  Max_L >100×Max_S and Max_L >100×Max_C | 0 |
| **Starvation**  **-upregulated genes** | Max_L > 99  Max_S > 2×Max_L | 3270 |
|  | Max_L > 99  Max_S > 5×Max_L | 616 |
|  | Max_L > 99  Max_S > 10×Max_L | 229 |
|  | Max_L > 99  Max_S > 50×Max_L | 33 |
|  | Max_L > 99  Max_S > 100×Max_L | 14 |
| **Conjugation**  **-upregulated genes** | Max_S > 99  Max_C > 2×Max_S | 6092 |
|  | Max_S > 99  Max_C > 5×Max_S | 1753 |
|  | Max_S > 99  Max_C > 10×Max_S | 714 |
|  | Max_S > 99  Max_C > 50×Max_S | 83 |
|  | Max_S > 99  Max_C > 100×Max_S | 30 |

* Max_L, Max_S and Max_C were the maximum signal intensity during Log growth, Starvation and Conjugation respectively.
