# Supplementary material for: Microarray Analyses of Gene Expression during the Tetrahymena thermophila Life Cycle
Source: PLoS One. 2009 Feb 10;4(2):e4429. doi: 10.1371/journal.pone.0004429 (PMC2636879; doi:10.1371/journal.pone.0004429)
Supplement: Table S6 — Ninety-one Growth-specific genes expressed at levels higher than 2× corrected background. (0.13 MB DOC) [file pone.0004429.s007.doc]

**Table S6. Ninety-one Growth-specific genes expressed at levels higher than 2X corrected background.**

**Cluster a (35 genes)**

| **Gene ID** | **Gene annotation^*^** | **E value** |
| --- | --- | --- |
| TTHERM_00675720 | PREDICTED: hypothetical protein XP_538672 [Canis familiaris] | 0.28 |
| TTHERM_00171880 | Predicted Tetrahymena ORF ^a^ | - |
| TTHERM_00821880 | RNA-binding domain protein | 8e-157 |
| TTHERM_01009880 | Hemocyanin isoform 1 [Nucula nucleus] | 1.6 |
| TTHERM_01014730 | Ankyrin repeat protein, putative [Trichomonas vaginalis G3] | 2e-26 |
| TTHERM_00251150 | PREDICTED: similar to SEC63 homolog (S. cerevisiae) [Bos taurus] | 3.4 |
| TTHERM_01078050 | CG5131 CG5131-PA, isoform A [Drosophila melanogaster] | 0.004 |
| TTHERM_00600660 | Esterase YqiA [Vibrio harveyi HY01] | 5e-25 |
| 3686.m00957 ^b^ | AGAP008432-PA [Anopheles gambiae str. PEST] | 3e-05 |
| TTHERM_00338150 | AGAP005979-PA [Anopheles gambiae str. PEST] | 0.062 |
| TTHERM_00221080 | Predicted Tetrahymena ORF ^a^ | - |
| TTHERM_00446060 | F13M7.11 [Arabidopsis thaliana] | 1e-48 |
| TTHERM_00437520 | TPR Domain containing protein | 0 |
| TTHERM_00520890 | ATPase, histidine kinase-, DNA gyrase B-, and HSP90-like domain | 2.0 |
| TTHERM_00522560 | Diacylglycerol kinase, putative | 0 |
| TTHERM_00477030 | Predicted Tetrahymena ORF ^a^ | - |
| TTHERM_00560100 | PREDICTED: similar to sperm-specific sodium proton exchanger [Equus caballus] | 0.14 |
| TTHERM_00578850 | SCO1/SenC family protein | 0 |
| TTHERM_00661500 | Mitochondrial carrier protein | 9e-177 |
| TTHERM_00086830 | Protein-L-isoaspartate(D-aspartate) O-methyltransferase (PCMT) |  |
| TTHERM_00122460 | Methyltransferase type 11 [Caldicellulosiruptor saccharolyticus DSM 8903] | 3e-20 |
| TTHERM_00954260 | Serpin, serine protease inhibitor. | 0 |
| TTHERM_01006460 | Elongation factor-1 alpha [Isohypsibius elegans] | 0.67 |
| TTHERM_01125220 | ARID/BRIGHT DNA binding domain containing protein [Plasmodium vivax SaI-1] | 3.9 |
| TTHERM_01289180 | Predicted Tetrahymena ORF ^a^ | - |
| TTHERM_00035690 | TPR Domain containing protein | 0 |
| TTHERM_01020700 | ABC1 family protein. Sequence similarity to the ABC1 protein kinase | 0 |
| TTHERM_00699790 | REJ domain containing protein | 0 |
| TTHERM_01108630 | W05G11.6b [Caenorhabditis elegans] | 1.3 |
| TTHERM_00392890 | PREDICTED: similar to CG3184-PA [Tribolium castaneum] | 3e-21 |
| TTHERM_00085310 | Oxidoreductase, short chain dehydrogenase/reductase family protein | 1e-148 |
| TTHERM_00448770 | Alkaline phosphatase [Streptomyces coelicolor A3(2)] | 3.4 |
| TTHERM_00643490 | ubiE/COQ5 methyltransferase-related | 8e-175 |
| TTHERM_01075770 | Unnamed protein product [Vitis vinifera] | 0.13 |
| TTHERM_01034440 | Multicystatin (MC) | 3e-06 |

**Cluster b (10 genes)**

| **Gene ID** | **Gene annotation ^*^** | **E value** |
| --- | --- | --- |
| TTHERM_00697570 | PREDICTED: phosphodiesterase 3B, cGMP-inhibited [Macaca mulatta] | 1.0 |
| TTHERM_00885830 | Cyclic nucleotide-binding domain containing protein | 0 |
| TTHERM_01015980 | Beta-hydroxybutyryl-CoA dehydrogenase [Butyrivibrio fibrisolvens] | 0.35 |
| TTHERM_01088000 | REJ domain containing protein [Tetrahymena thermophila SB210] | 2e-164 |
| TTHERM_01125160 | Nucleotide-sugar transporter | 9e-47 |
| TTHERM_00408820 | N-acetyltransferase [Plesiocystis pacifica SIR-1] | 2e-07 |
| TTHERM_00417990 | Insect antifreeze protein | 0.0 |
| TTHERM_01600640 | REJ domain containing | 1e-161 |
| TTHERM_00561540 | Eukaryotic aspartyl protease family protein | 0 |
| TTHERM_00257180 | Os01g0142200 [Oryza sativa (japonica cultivar-group)] | 0.025 |

**Cluster c (7 genes)**

| **Gene ID** | **Gene annotation ^*^** | **E value** |
| --- | --- | --- |
| TTHERM_00794010 | MATE efflux family | 0 |
| TTHERM_00939190 | Predicted Tetrahymena ORF ^a^ | - |
| TTHERM_01166300 | Predicted Tetrahymena ORF ^a^ | - |
| TTHERM_01197150 | ABC1 family protein. Sequence similarity to the ABC1 protein kinase | 0 |
| TTHERM_00051710 | CG1 protein [Plasmodium falciparum] | 0.99 |
| TTHERM_00485980 | Helicase conserved C-terminal domain containing protein | 0 |
| TTHERM_00840040 | AGAP008857-PA [Anopheles gambiae str. PEST] | 0.001 |

**Cluster d (8 genes)**

| **Gene ID** | **Gene annotation ^*^** | **E value** |
| --- | --- | --- |
| TTHERM_00798140 | Major Facilitator Superfamily protein | 0 |
| TTHERM_00802360 | Transmembrane amino acid transporter protein | 0 |
| TTHERM_00895790 | Predicted protein histone methylation protein DOT1 [Ostreococcus ucimarinus CCE9901] | 2e-10 |
| TTHERM_00034920 | ABC transporter family protein | 0 |
| 3723.m00730 ^b^ | Glutathione S-transferase Mu | 2e-117 |
| TTHERM_00543570 | EGF-like domain containing protein | 0 |
| TTHERM_00569180 | Resolvase [Vibrionales bacterium SWAT-3] | 1.7 |
| TTHERM_00572180 | MAPEG family protein | 4e-99 |

**Cluster e (6 genes)**

| **Gene ID** | **Gene annotation ^*^** | **E value** |
| --- | --- | --- |
| TTHERM_00798150 | Oxalate/Formate Antiporter protein | 0 |
| TTHERM_00903970 | Cytochrome P450 family | 8e-08 |
| TTHERM_01188340 | Transmembrane amino acid transporter protein | 0 |
| TTHERM_00569210 | Glutathione S-transferase, N-terminal domain containing protein | 0 |
| TTHERM_01002680 | HAD-superfamily subfamily IIA hydrolase, TIGR01456, CECR5 containing protein | 2e-34 |
| TTHERM_00343640 | Predicted protein [Coprinopsis cinerea okayama7#130] | 0.51 |

**Cluster f (9 genes)**

| **Gene ID** | **Gene annotation ^*^** | **E value** |
| --- | --- | --- |
| TTHERM_00820740 | Pectinacetylesterase family protein | 0 |
| TTHERM_01006530 | Neurohypophysial hormones, N-terminal Domain containing protein | 0 |
| TTHERM_01079230 | Predicted Tetrahymena ORF ^a^ | - |
| TTHERM_00600070 | ABC transporter family protein | 0 |
| TTHERM_00559790 | S1/P1 Nuclease | 8e-26 |
| TTHERM_01403830 | Ku70/Ku80 beta-barrel domain containing protein | 2e-38 |
| TTHERM_00075660 | Predicted Tetrahymena ORF ^a^ | - |
| TTHERM_00305480 | Predicted Tetrahymena ORF ^a^ | - |
| TTHERM_00312030 | Predicted Tetrahymena ORF ^a^ | - |

**Cluster g (12 genes)**

| **Gene ID** | **Gene annotation ^*^** | **E value** |
| --- | --- | --- |
| TTHERM_00885790 | Acyl transferase domain containing protein | 0 |
| TTHERM_00904010 | Protein kinase domain containing | 3e-06 |
| TTHERM_00029910 | Predicted Tetrahymena ORF ^a^ | - |
| TTHERM_01146060 | Polypeptide deformylase family protein | 0 |
| TTHERM_01307940 | Ubiquitin family protein | 7e-15 |
| TTHERM_00723520 | Transcriptional regulator, LysR family [Acidovorax avenae subsp. citrulli AAC00-1] | 1.5 |
| TTHERM_00473230 | Glutathione S-transferase, N-terminal domain containing | 2e-24 |
| TTHERM_00502350 | EF hand family protein | 0 |
| TTHERM_00448990 | Predicted Tetrahymena ORF ^a^ | - |
| TTHERM_00656070 | Hydrolase, NUDIX family protein | 3e-85 |
| TTHERM_00157980 | Protein kinase domain containing protein | 2e-06 |
| TTHERM_00433650 | TPR Domain containing protein. Sequence similarity to a protein kinase family unique to Tetrahymena thermophila | 0 |

**Cluster h (4 genes)**

| **Gene ID** | **Gene annotation ^*^** | **E value** |
| --- | --- | --- |
| TTHERM_00196340 | PREDICTED: similar to importin beta-3 [Nasonia vitripennis] | 1e-05 |
| TTHERM_01144970 | Neurohypophysial hormones, N-terminal Domain containing protein | 5e-42 |
| TTHERM_00344080 | ABC transporter family protein | 0 |
| TTHERM_00842570 | Predicted Tetrahymena ORF ^a^ | 0 |

Footnotes *, a and b as in Table S3
