# Supplementary material for: Microarray Analyses of Gene Expression during the Tetrahymena thermophila Life Cycle
Source: PLoS One. 2009 Feb 10;4(2):e4429. doi: 10.1371/journal.pone.0004429 (PMC2636879; doi:10.1371/journal.pone.0004429)
Supplement: Table S7 — Ninety starvation-specific genes expressed at levels higher than 2× corrected background. (0.12 MB DOC) [file pone.0004429.s008.doc]

**Table S7. Ninety starvation-specific genes expressed at levels higher than 2X corrected background.**

Cluster a (9 genes)

| **Gene ID** | **Gene annotation^*^** | **E value** |
| --- | --- | --- |
| TTHERM_00225750 | ADP-ribosylation factor family protein | 6e-25 |
| TTHERM_01141630 | ADP-ribosylation factor family protein | 2e-25 |
| TTHERM_00123660 | Predicted Tetrahymena ORF ^a^ | - |
| TTHERM_00013260 | Predicted Tetrahymena ORF ^a^ | - |
| TTHERM_00616080 | Predicted Tetrahymena ORF ^a^ |  |
| TTHERM_00378410 | Predicted Tetrahymena ORF ^a^ | - |
| TTHERM_00430050 | Predicted Tetrahymena ORF ^a^ | - |
| TTHERM_00450860 | Ubiquitin carboxyl-terminal hydrolase family protein | 0 |
| TTHERM_00450910 | Ubiquitin carboxyl-terminal hydrolase family protein | 3e-54 |

Cluster b (13 genes)

| **Gene ID** | **Gene annotation ^*^** | **E value** |
| --- | --- | --- |
| TTHERM_00292110 | 14-3-3 protein | 6e-154 |
| TTHERM_00414540 | ABC transporter family protein | 0 |
| TTHERM_00695840 | Cyclic nucleotide-binding domain containing protein | 6e-38 |
| TTHERM_00620900 | Lyase 2 | 6.0 |
| TTHERM_00834900 | Neurohypophysial hormones, N-terminal Domain containing protein | 6e-40 |
| TTHERM_00516410 | Neurohypophysial hormones, N-terminal Domain containing protein | 3e-11 |
| TTHERM_00698610 | Predicted Tetrahymena ORF ^a^ | - |
| TTHERM_00249590 | Predicted Tetrahymena ORF ^a^ | - |
| TTHERM_01301780 | Predicted Tetrahymena ORF ^a^ | - |
| TTHERM_00951810 | Protein kinase domain containing protein | 1e-59 |
| TTHERM_00951820 | Protein kinase domain containing protein | 4e-55 |
| TTHERM_00128700 | TPR Domain containing protein | 0 |
| TTHERM_00956490 | Translation initiation factor eIF-5A family protein | 6e-50 |

Cluster c (34 genes)

| **Gene ID** | **Gene annotation ^*^** | **E value** |
| --- | --- | --- |
| TTHERM_01151460 | CARD15-like protein-related | 4e-148 |
| 139.m00942 ^b^ | Cyclic nucleotide-binding domain containing protein | 3e-10 |
| TTHERM_00639930 | DNA-directed DNA polymerase [Methylobacterium hloromethanicum CM4] | 2.9 |
| TTHERM_01299640 | Eukaryotic-type carbonic anhydrase family protein | 2e-25 |
| TTHERM_00257060 | Helicase conserved C-terminal domain containing protein | 2e-07 |
| TTHERM_00249670 | L-asparaginase, type I [Shewanella pealeana ATCC 700345] | 1.4 |
| TTHERM_01576270 | Leucine Rich Repeat family protein | 4e-09 |
| TTHERM_00461810 | Leucine Rich Repeat family protein | 1e-09 |
| TTHERM_00835410 | Neurohypophysial hormones, N-terminal Domain containing protein | 9e-11 |
| TTHERM_00974090 | Neurohypophysial hormones, N-terminal Domain containing protein | 1e-30 |
| TTHERM_01250140 | Neurohypophysial hormones, N-terminal Domain containing protein | 1e-12 |
| TTHERM_01122760 | Predicted Tetrahymena ORF ^a^ | - |
| TTHERM_00527130 | Predicted Tetrahymena ORF ^a^ | - |
| TTHERM_00429980 | Predicted Tetrahymena ORF ^a^ | - |
| TTHERM_00324350 | Polyketide synthetase domain protein [Pseudomonas syringae pv.  phaseolicola 1448A] | 0.96 |
| TTHERM_00713000 | Polyprotein [East Asian Passiflora virus] | 0.74 |
| TTHERM_00820750 | Protein kinase domain containing protein | 4e-20 |
| TTHERM_01076930 | Protein kinase domain containing protein | 2e-08 |
| TTHERM_01122750 | Protein kinase domain containing protein | 0.003 |
| TTHERM_01146020 | Protein kinase domain containing protein | 4e-06 |
| TTHERM_00515260 | Protein kinase domain containing protein | 1e-08 |
| TTHERM_00571730 | Protein kinase domain containing protein | 5e-23 |
| TTHERM_00581720 | Protein kinase domain containing protein | 2e-21 |
| 79.m01548 ^b^ | Protein kinase domain containing protein | 6e-10 |
| 32.m02511 ^b^ | Rab2 | 1e-19 |
| TTHERM_00951850 | SLEI family protein | 3e-31 |
| TTHERM_00128980 | Small GTP-binding protein, putative [Trypanosoma brucei] | 2e-04 |
| TTHERM_00939010 | Steroidogenic acute regulatory protein [Gadus morhua] | 8e-06 |
| TTHERM_01002700 | Syntaxin-73, putative | 7e-10 |
| TTHERM_01138270 | TPR Domain containing protein | 5e-27 |
| TTHERM_01676220 | TPR Domain containing protein | 2e-07 |
| TTHERM_00666410 | TRAF-type zinc finger family protein | 0 |
| TTHERM_00257050 | Type III restriction enzyme, res subunit family protein | 7e-04 |
| TTHERM_00297190 | Uncharacterized ACR, COG1434 family protein | 5e-106 |

Cluster d (14 genes)

| **Gene ID** | **Gene annotation ^*^** | **E value** |
| --- | --- | --- |
| TTHERM_00148930 | 3'5'-cyclic nucleotide phosphodiesterase family protein | 0 |
| TTHERM_00494730 | EF hand family protein | 1e-08 |
| TTHERM_00561220 | EF hand family protein. Sequence similarity to a protein kinase family unique to *Tetrahymena thermophila* | 0 |
| TTHERM_01009860 | Leishmanolysin family protein | 0 |
| TTHERM_00794220 | Leucine Rich Repeat family protein | 0 |
| TTHERM_00233020 | Predicted Tetrahymena ORF ^a^ | - |
| TTHERM_00129730 | Predicted Tetrahymena ORF ^a^ | - |
| TTHERM_00647000 | Predicted Tetrahymena ORF ^a^ | - |
| TTHERM_00077810 | Predicted Tetrahymena ORF ^a^ | - |
| TTHERM_00122470 | Predicted Tetrahymena ORF ^a^ | - |
| TTHERM_00561750 | Protein of unknown function DUF262 [Anabaena variabilis ATCC  29413] | 0.27 |
| TTHERM_00257090 | Putative transmembrane protein [Pedobacter sp. BAL39] | 1.6 |
| TTHERM_00490530 | UvrD/REP helicase family protein | 0 |
| TTHERM_01323720 | Zinc finger in N-recognin family protein | 4e-55 |

Cluster e (20 genes)

| **Gene ID** | **Gene annotation ^*^** | **E value** |
| --- | --- | --- |
| TTHERM_00440510 | Eukaryotic aspartyl protease family protein. Sequence similarity to a protein kinase family unique to Tetrahymena thermophila | 0 |
| TTHERM_00561380 | F35E12.4 [Caenorhabditis elegans] | 9.0 |
| TTHERM_00588860 | F-box domain containing protein | 1e-42 |
| TTHERM_00825140 | Histidine kinase internal region [Clostridium phytofermentans ISDg] | 9.5 |
| TTHERM_01029900 | M-phase phosphoprotein 11 [Culex pipiens quinquefasciatus] | 8.8 |
| TTHERM_00600750 | Myosin head domain protein | 2e-12 |
| TTHERM_00401870 | Neurohypophysial hormones, N-terminal Domain containing protein | 2.0 |
| TTHERM_01013120 | Predicted Tetrahymena ORF ^a^ | - |
| TTHERM_00048780 | Predicted Tetrahymena ORF ^a^ | - |
| TTHERM_00300060 | Predicted Tetrahymena ORF ^a^ | - |
| TTHERM_00355480 | Predicted Tetrahymena ORF ^a^ | - |
| TTHERM_00561390 | Predicted Tetrahymena ORF ^a^ | - |
| TTHERM_00678070 | Predicted Tetrahymena ORF ^a^ | - |
| TTHERM_00836710 | Predicted Tetrahymena ORF ^a^ | - |
| TTHERM_00490960 | Predicted Tetrahymena ORF ^a^ | - |
| TTHERM_00491120 | Predicted Tetrahymena ORF ^a^ | - |
| TTHERM_00581570 | Predicted Tetrahymena ORF ^a^ | - |
| TTHERM_00484730 | Putative ATP-dependent RNA helicase [Shewanella benthica KT99] | 0.55 |
| TTHERM_00142240 | SRP54-type protein, GTPase domain containing protein | 0 |
| TTHERM_00999110 | UbiE/COQ5 methyltransferase, putative | 5e-79 |

Footnotes *, a and b as in Table S3.
