# Supplementary material for: Microarray Analyses of Gene Expression during the Tetrahymena thermophila Life Cycle
Source: PLoS One. 2009 Feb 10;4(2):e4429. doi: 10.1371/journal.pone.0004429 (PMC2636879; doi:10.1371/journal.pone.0004429)
Supplement: Table S8 — Genes co-expressed with putative transposase, TTHERM_01107220. (0.12 MB DOC) [file pone.0004429.s009.doc]

**Table S8. Genes co-expressed with putative transposase, TTHERM_01107220.**

| **Gene ID** | **R value** | **Gene annotation ^*^** | **E value** |
| --- | --- | --- | --- |
| TTHERM_01107220 | 1 | Transposase [Heliothis virescens] | 6e-22 |
| TTHERM_00672060 | 0.996 | Predicted Tetrahymena ORF ^a^ | - |
| TTHERM_00185830 | 0.994 | p28 protein | 6e-05 |
| TTHERM_00947380 | 0.984 | Predicted Tetrahymena ORF ^a^ | - |
| TTHERM_00424710 | 0.977 | Protein kinase domain containing protein | 1e-25 |
| TTHERM_00317460 | 0.975 | Predicted Tetrahymena ORF ^a^ | - |
| 22.m02619 ^b^ | 0.975 | 5'-nucleotidase/2' 3'-cyclic phosphodiesterase and related esterases-like  protein [Epulopiscium sp. 'N.t. morphotype B'] | 9e-06 |
| TTHERM_00035670 | 0.974 | Serine carboxypeptidase S28 family protein | - |
| TTHERM_00066910 | 0.9734 | CAMK family protein kinase [Trichomonas vaginalis G3] | 8e-16 |
| TTHERM_00201750 | 0.972 | Predicted Tetrahymena ORF ^a^ | - |
| TTHERM_01251270 | 0.971 | TPR repeat domain protein [Thiomicrospira crunogena XCL-2] | 2.1 |
| TTHERM_00962160 | 0.970 | Cyclin, N-terminal domain containing protein | 0 |
| TTHERM_00427480 | 0.969 | Endonuclease/Exonuclease/phosphatase family protein | 0 |
| TTHERM_00142430 | 0.968 | RING Zn finger-containing protein [Dictyostelium discoideum AX4] | e-07 |
| TTHERM_01026380 | 0.968 | Predicted Tetrahymena ORF ^a^ | - |
| TTHERM_00925580 | 0.968 | CHP-rich zinc finger protein-like [Oryza sativa Japonica Group] | 6e-05 |
| TTHERM_01369770 | 0.966 | Cytochrome P450 family protein | 0 |
| TTHERM_00196580 | 0.963 | Predicted Tetrahymena ORF ^a^ | - |
| TTHERM_00717910 | 0.962 | Serine/threonine kinase 36 [Homo sapiens] | 4.00E-09 |
| TTHERM_00129970 | 0.960 | Lpx1p [Saccharomyces cerevisiae] | 0.33 |
| TTHERM_00616370 | 0.957 | Cyclic nucleotide-binding domain containing protein | 1e-18 |
| TTHERM_01197010 | 0.955 | Ubiquitin carboxyl-terminal hydrolase family protein | 3e-132 |
| TTHERM_00095520 | 0.954 | TPR Domain containing protein | 8e-48 |
| TTHERM_00800250 | 0.9544 | COG0838: NADH:ubiquiNone oxidoreductase subunit 3 (chain A) [Magnetospirillum  magnetotacticum MS-1] | 1.1 |
| TTHERM_00201680 | 0.954 | *RAB48* | 4e-107 |
| TTHERM_00188740 | 0.950 | Cyclic nucleotide-binding domain containing protein | 1e-74 |
| TTHERM_00633600 | 0.949 | Kinesin motor domain containing protein | 5e-40 |
| TTHERM_00363110 | 0.948 | Immobilization antigen LD, putative | 8e-58 |
| TTHERM_00463580 | 0.948 | Major facilitator superfamily protein | 6e-04 |
| TTHERM_00049050 | 0.947 | Predicted Tetrahymena ORF ^a^ | - |
| TTHERM_01006610 | 0.944 | L-fucose isomerase [Akkermansia muciniphila ATCC BAA-835] | 3.5 |
| TTHERM_00310070 | 0.942 | Metal ion binding / oxidoreductase [Arabidopsis thaliana] | 6.3 |
| TTHERM_00962170 | 0.942 | Predicted Tetrahymena ORF ^a^ | - |
| TTHERM_00728920 | 0.942 | Cyclic nucleotide-binding domain containing protein | 2e-07 |
| TTHERM_00149800 | 0.939 | LIM domain containing protein | 1e-05 |
| TTHERM_01137160 | 0.939 | Cell surface immobilization antigen SerH5-related Neurohypophysial hormones | 3e-83 |
| TTHERM_00912250 | 0.938 | ABC transporter family protein | 2e-159 |
| TTHERM_00812810 | 0.938 | Predicted Tetrahymena ORF ^a^ | - |
| TTHERM_00052490 | 0.936 | Novel protein containing WD domains [Mus musculus] | 1e-90 |
| TTHERM_01415190 | 0.935 | Repeated sequence found in lipoprotein LPP containing protein | 7e-14 |
| TTHERM_00489470 | 0.933 | Neurohypophysial hormones, N-terminal Domain containing protein | 2e-15 |
| TTHERM_00818310 | 0.933 | Protein of unknown function DUF885 [Shewanella woodyi ATCC 51908] | 4.0 |
| TTHERM_00227830 | 0.931 | Glycosyl hydrolases family 38 protein | 0 |
| TTHERM_00079670 | 0.931 | Papain family cysteine protease containing protein [Tetrahymena thermophila SB210] | 0 |
| TTHERM_00158240 | 0.931 | Tetratricopeptide domain protein [Bacillus cereus subsp. cytotoxis NVH 391-98] | 2.1 |
| TTHERM_01205240 | 0.930 | Predicted Tetrahymena ORF ^a^ | - |
| TTHERM_01035590 | 0.930 | Leucine Rich Repeat family protein | 1e-37 |
| TTHERM_00774830 | 0.929 | Protein kinase domain containing protein | 7e-45 |
| TTHERM_00083470 | 0.929 | Predicted Tetrahymena ORF ^a^ | - |
| TTHERM_01154640 | 0.928 | WD domain, G-beta repeat protein | 0 |
| TTHERM_00085110 | 0.927 | Phosphatidylinositol-specific phospholipase C | 5e-33 |
| TTHERM_00594240 | 0.927 | DHHC zinc finger domain containing protein | 5e-08 |
| TTHERM_00654170 | 0.926 | Predicted Tetrahymena ORF ^a^ | - |
| TTHERM_00354770 | 0.924 | Predicted Tetrahymena ORF ^a^ | - |
| TTHERM_00318750 | 0.922 | Regulator of Presynaptic Morphology family member (rpm-1) [Caenorhabditis elegans] | 5e-72 |
| TTHERM_00237570 | 0.921 | WD-40 repeat [Trichodesmium erythraeum IMS101] | 2e-25 |
| TTHERM_00238850 | 0.921 | Phosphatidylinositol-specific phospholipase C [Homo sapiens] | 6e-78 |
| TTHERM_00718080 | 0.920 | Surface protein with furin-like cysteine repeats, putative [Paramecium tetraurelia strain d4-2] | 0.001 |
| TTHERM_01141610 | 0.920 | Serine carboxypeptidase family protein | 1e-66 |
| TTHERM_00138590 | 0.919 | IPT/TIG domain containing protein | 5e-109 |
| TTHERM_00616720 | 0.918 | WW domain containing protein | 2e-24 |
| TTHERM_00091700 | 0.917 | Predicted Tetrahymena ORF ^a^ | - |
| TTHERM_00323140 | 0.917 | Neurohypophysial hormones, N-terminal Domain containing protein  High cysteine membrane protein Group 2 [Giardia lamblia ATCC 50803] | 7e-75 |
| TTHERM_00035430 | 0.916 | ABC transporter family protein | 6e-76 |
| TTHERM_00628500 | 0.913 | WD domain, G-beta repeat protein | 0 |
| TTHERM_00734120 | 0.913 | Protein kinase domain containing protein | 5e-41 |
| TTHERM_00997800 | 0.913 | Cytochrome c oxidase, subunit IIc [Sphingomonas sp. SKA58] | 1.0 |
| TTHERM_02424110 | 0.912 | Matrilin-2 precursor –related | 7e-75 |
| TTHERM_00247090 | 0.912 | SF-assemblin/beta giardin family protein | 3e-25 |
| TTHERM_00046530 | 0.910 | Cyclic nucleotide-binding domain containing protein. Sequence similarity to a protein kinase family unique to Tetrahymena thermophila | 0 |
| TTHERM_00417940 | 0.910 | Phosphoesterase family protein | 1e-79 |
| TTHERM_00630360 | 0.910 | Zinc finger domain, LSD1 subclass family protein | 3e-31 |
| TTHERM_00189140 | 0.910 | Leucine rich repeat containing 49, isoform CRA_a [Mus musculus] | 2e-41 |
| TTHERM_00532140 | 0.910 | Phosphatidylserine decarboxylase family protein | 1e-25 |
| TTHERM_00317110 | 0.909 | Eukaryotic-type carbonic anhydrase family protein | 0.004 |
| TTHERM_00355440 | 0.908 | Protein kinase domain containing protein. Sequence similarity to a protein kinase family unique to Tetrahymena thermophila | 2e-26 |
| TTHERM_00046900 | 0.908 | TLD family protein, Histidine acid phosphatase family protein | 2e-48 |
| TTHERM_00694440 | 0.907 | Zinc finger domain, LSD1 subclass family protein [Tetrahymena thermophila SB210] | - |
| TTHERM_00016550 | 0.906 | Steroid dehydrogenase [Culex pipiens quinquefasciatus] | 1.2 |
| TTHERM_00410190 | 0.904 | Protein kinase domain containing protein | 1e-14 |
| TTHERM_00784410 | 0.903 | EF hand family protein, putative calcium/calmodulin dependent protein kinase [Zea mays] | 1e-09 |
| TTHERM_00735380 | 0.903 | Phage head-tail adaptor, putative family protein [Tetrahymena thermophila SB210] | 4e-40 |
| TTHERM_00312900 | 0.902 | Leucine Rich Repeat family protein | 0 |
| TTHERM_01001490 | 0.901 | SD07741p [Drosophila melanogaster] | 1e-17 |
| TTHERM_00310060 | 0.900 | Surface protein with furin-like cysteine repeats, putative [Paramecium tetraurelia strain d4-2] | 4e-33 |
| TTHERM_00695880 | 0.900 | Etratricopeptide TPR_2 repeat protein [Herpetosiphon aurantiacus ATCC 23779] | 3e-05 |

Footnotes *, a and b as in Table S3.
