# Supplementary material for: Microarray Analyses of Gene Expression during the Tetrahymena thermophila Life Cycle
Source: PLoS One. 2009 Feb 10;4(2):e4429. doi: 10.1371/journal.pone.0004429 (PMC2636879; doi:10.1371/journal.pone.0004429)
Supplement: Table S9 — Fifty-one Conjugation-induced/specific transcription factors of Tetrahymena thermophila. (0.09 MB DOC) [file pone.0004429.s010.doc]

**Table S9. Fifty-one Conjugation-induced/specific transcription factors of *Tetrahymena thermophila***

| **Expression peak (hr)** | **Gene ID** | **Description of homologs in TGD**  **(http://db.ciliate.org/cgi-bin/search/textSearch?query=transcription+factor&type=homolog)** |
| --- | --- | --- |
| 2 | TTHERM_00313290 | **transcription factor**-related  TFIIH and nucleotide excision repair factor 3 complexes subunit (Tfb2), putative [Neosartorya fischeri NRRL 181] 2e-11 |
| 2 | TTHERM_00152050 | basic **transcription factor** 2, 44kD subunit-related  Transcription factor Ssl1 family protein component of the TFIIH |
| 2 | TTHERM_00420200 | General transcription factor IIH, polypeptide 3 |
| 2 | TTHERM_01496790 | Serine/threonine protein kinase, subunit of the **transcription factor** TFIIH; involved in transcription initiation at RNA polymerase II promoters |
| 4 | TTHERM_00227280 | Serine/threonine protein kinase, subunit of the **transcription factor** TFIIH; involved in transcription initiation at RNA polymerase II promoters |
| 6 | TTHERM_00058670 | Serine/threonine protein kinase, subunit of the **transcription factor** TFIIH; involved in transcription initiation at RNA polymerase II promoters |
| 10 | TTHERM_00144910 | Serine/threonine protein kinase, subunit of the **transcription factor** TFIIH; involved in transcription initiation at RNA polymerase II promoters |
| 2 | TTHERM_00401830 | XPB proteins are involved in both DNA repair and transcription, they are component of the **transcription factor** IIH (TFIIH) and are responsible for DNA helicase activity during nucleotide (nt) excision repair (NER). Complementation assays in yeast rad25 mutant strains suggest the involvement of AtXPB2 in DNA repair. |
| 2 | TTHERM_00818430 | XPB proteins are involved in both DNA repair and transcription, they are component of the **transcription factor** IIH (TFIIH) and are responsible for DNA helicase activity during nucleotide (nt) excision repair (NER). Complementation assays in yeast rad25 mutant strains suggest the involvement of AtXPB2 in DNA repair. |
| 4 | TTHERM_00028580 | KOW domain-containing **transcription factor** family protein |
|  | | |
|  | | |
|  | | |
| 2 | TTHERM_00047010 | DPB-1 **transcription factor**, putative (DPB) |
| 14 | TTHERM_00016400 | DPB-1 **transcription factor**, putative (DPB) |
| 8 | TTHERM_00107000 | PREDICTED: similar to **transcription factor** Dp-1 (E2F dimerization partner 1) (DRTF1-polypeptide-1) (DRTF1) isoform 1 |
| 14 | TTHERM_01099150 | E2F **transcription factor**-3 (E2F3) |
| 2 | TTHERM_00695710 | E2FC; E2F **transcription factor**-2 (E2F2) / **transcription factor** E2Fc (E2Fc) |
| 4 | TTHERM_01076950 | **transcription factor**, putative / E2F-like repressor E2L2 (E2L2) |
| 6 | TTHERM_00721610 | **transcription factor**, putative / E2F-like repressor E2L2 (E2L2) |
|  | | |
| 6 | TTHERM_00334420 | myb family **transcription factor** |
| 6 | TTHERM_00357110 | myb family **transcription factor** |
| 12 | TTHERM_00131150 | myb family **transcription factor** |
| 4 | TTHERM_00571890 | myb family **transcription factor** (MYB98) |
| 4 | TTHERM_00842380 | similar to myb family **transcription factor** [Arabidopsis thaliana] |
| 6 | TTHERM_00202940 | similar to myb-related **transcription factor** (CCA1) [Arabidopsis thaliana] |
|  | | |
| 2 | TTHERM_00298500 | DNA repair protein / **transcription factor** protein (UVH6) |
| 4 | TTHERM_00316410 | DNA repair protein / **transcription factor** protein (UVH6) |
| 6 | TTHERM_00586840 | DNA repair protein / **transcription factor** protein (UVH6) |
|  | | |
| 2 | TTHERM_00766440 | PHD finger **transcription factor**, putative |
| 4 | TTHERM_00446020 | Similar to fetal Alzheimer antigen isoform 1; bromodomain and PHD domain **transcription factor**; nucleosome remodeling factor, large subunit; fetal Alz-50 reactive clone 1 |
| 6 | TTHERM_00219200 | Similar to fetal Alzheimer antigen isoform 1; bromodomain and PHD domain **transcription factor**; nucleosome remodeling factor, large subunit; fetal Alz-50 reactive clone 1 |
|  | | |
| 2 | TTHERM_00600840 | **transcription factor**-like related cluster  Tesmin/TSO1-like CXC domain containing protein  transcription factor CPP [Lotus japonicus] |
| 4 | TTHERM_00766460 | Putative **transcription factor** related cluster transcription factor CPP [Lotus japonicus] |
| 4 | TTHERM_00577120 | Putative **transcription factor** related cluster transcription factor CPP [Lotus japonicus] |
|  | | |
| 6 | TTHERM_00239260 | similar to zinc finger (C2H2 type) family protein / **transcription factor** jumonji (jmj) family protein [Arabidopsis thaliana] |
| 12 | TTHERM_00657430 | **transcription factor** umonji (jmj) family protein / zinc finger (C5HC2 type) family protein |
| 6 | TTHERM_00780520 | **transcription factor** jumonji (jmjC) domain-containing protein |
|  | | |
| 6 | TTHERM_00647360 | Transcription regulatory protein SNF5 (SWI/SNF complex component SNF5) (**transcription factor** TYE4). |
| 12 | TTHERM_00217200 | Transcription regulatory protein SNF5 (SWI/SNF complex component SNF5) (**transcription factor** TYE4). |
| 14 | TTHERM_01020870 | Transcription regulatory protein SNF5 (SWI/SNF complex component SNF5) (**transcription factor** TYE4). |
|  | | |
| 4 | TTHERM_00446570 | similar to CCAAT-box binding **transcription factor** Hap5a, putative [Arabidopsis thaliana] |
| 6 | TTHERM_00852840 | similar to CCAAT-box binding **transcription factor** Hap5a, putative [Arabidopsis thaliana] |
|  | | |
| 8 | TTHERM_00683400 | Splice Isoform 1 of General **transcription factor** 3C polypeptide 3 |
| 2 | TTHERM_00581820 | Splice Isoform 2 of General **transcription factor** 3C polypeptide 5 |
|  | | |
| 8 | TTHERM_00575350 | TATA-binding protein, general **transcription factor** that interacts with other factors to form the preinitiation complex at promoters, essential for viability |
| 10 | TTHERM_00082170 | TATA-binding protein, general **transcription factor** that interacts with other factors to form the preinitiation complex at promoters, essential for viability |
|  | | |
| 6 | TTHERM_00571940 | Nuclear response regulator and **transcription factor**, part of a branched two-component signaling system; required for optimal induction of heat-shock genes in response to oxidative stress; involved in osmoregulation |
| 4 | TTHERM_00442590 | Toxoplasma gondii **transcription factor** IIIB subunit, putative |
| 6 | TTHERM_00237370 | **transcription factor** IIB |
| 8 | TTHERM_00835000 | heat shock factor protein 4 (HSF4) / heat shock **transcription factor** 4 (HSTF4) |
| 10 | TTHERM_00734030 | MADS-box **transcription factor** FBP28 related cluster |
| 10 | TTHERM_00492920 | warA homeodomain (HOX) containing protein putative homeobox **transcription factor** |
| 10 | TTHERM_00047590 | Similar to CA0180|CaSPT20 Candida albicans CaSPT20 **transcription factor** related cluster |
