# Supplementary material for: Microarray Analyses of Gene Expression during the Tetrahymena thermophila Life Cycle
Source: PLoS One. 2009 Feb 10;4(2):e4429. doi: 10.1371/journal.pone.0004429 (PMC2636879; doi:10.1371/journal.pone.0004429)
Supplement: Table S10 — NF-Y (TTHERM_00439030) co-expressed genes. (0.17 MB DOC) [file pone.0004429.s011.doc]

**Table S10. NF-Y (TTHERM_00439030) co-expressed genes.**

| **Gene ID** | **R value** | **Gene annotation ^*^** | **E value** |
| --- | --- | --- | --- |
| TTHERM_00439030 | 0.99 | Retinoblastoma protein [Notophthalmus viridescens] | 3e-14 |
| TTHERM_00841200 | 0.99 | Kinesin motor domain containing protein | 0 |
| TTHERM_01443840 | 0.99 | TPA: transcription factor protein [Ciona intestinalis] | 0.004 |
| TTHERM_00704040 | 0.99 | MT-A70 family protein | 0 |
| TTHERM_01179960 | 0.99 | XPG I-region family protein | 0 |
| TTHERM_00483610 | 0.99 | ATPase, AAA family protein | 0 |
| TTHERM_00309890 | 0.99 | Predicted Tetrahymena ORF ^a^ | - |
| TTHERM_01027700 | 0.99 | *NRK9* | 0 |
| TTHERM_00426340 | 0.99 | Predicted Tetrahymena ORF ^a^ | - |
| TTHERM_00051920 | 0.99 | Predicted Tetrahymena ORF ^a^ | - |
| TTHERM_00683180 | 0.989 | DNA repair helicase |  |
| TTHERM_00614820 | 0.99 | DNA polymerase alpha-primase complex B subunit [Pichia stipitis CBS 6054] | 5e-18 |
| TTHERM_00213600 | 0.99 | Replication factor C 5 [Mus musculus] | 1e-76 |
| TTHERM_00592670 | 0.98 | TonB-dependent receptor [Flavobacterium johnsoniae UW101] | 0.28 |
| 47.m02288 ^b^ | 0.98 | Predicted Tetrahymena ORF ^a^ | - |
| TTHERM_00716000 | 0.98 | Predicted Tetrahymena ORF ^a^ | - |
| TTHERM_00444760 | 0.98 | Predicted Tetrahymena ORF ^a^ | - |
| TTHERM_00161180 | 0.98 | Replication factor C subunit, putative | 0.0 |
| TTHERM_00048980 | 0.98 | DBP2 [Arabidopsis thaliana] | 8e-35 |
| TTHERM_00058860 | 0.98 | Predicted Tetrahymena ORF ^a^ | - |
| TTHERM_00985110 | 0.98 | Predicted Tetrahymena ORF ^a^ | - |
| TTHERM_00426330 | 0.98 | Predicted Tetrahymena ORF ^a^ | - |
| TTHERM_00243710 | 0.98 | Eukaryotic-type DNA primase, large subunit family protein | 0 |
| TTHERM_00670150 | 0.98 | Predicted Tetrahymena ORF ^a^ | - |
| TTHERM_00762900 | 0.98 | ATPase, AAA family protein | 0 |
| TTHERM_00420910 | 0.98 | WD repeat and HMG-box DNA binding protein 1 isoform 2 [Homo sapiens] | 3e-19 |
| TTHERM_00399190 | 0.98 | Receptor-type adenylate cyclase GRESAG 4, putative [Trypanosoma  brucei TREU927] | 2.2 |
| TTHERM_01122690 | 0.98 | GA21324-PA [Drosophila pseudoobscura] | 0.42 |
| TTHERM_00773360 | 0.98 | Predicted Tetrahymena ORF ^a^ | - |
| TTHERM_00146340 | 0.98 | Core histone H2A/H2B/H3/H4 family protein | 4e-105 |
| 82.m01571 ^b^ | 0.98 | Predicted Tetrahymena ORF ^a^ | - |
| TTHERM_00399200 | 0.97 | Zinc finger (C3HC4-type RING finger) family protein [Arabidopsis  thaliana] | 0.085 |
| TTHERM_00576750 | 0.97 | Predicted Tetrahymena ORF ^a^ | - |
| TTHERM_00730330 | 0.97 | Microrchidia [Homo sapiens] | 5.0 |
| TTHERM_00105150 | 0.97 | RAN binding protein 5, isoform CRA_b [Mus musculus] | 1e-64 |
| TTHERM_00048880 | 0.97 | Predicted Tetrahymena ORF ^a^ | - |
| TTHERM_00292160 | 0.97 | FGGY family of carbohydrate kinases, N-terminal domain containing protein | 0 |
| TTHERM_00145680 | 0.97 | TPR Domain containing protein | 0 |
| 21.m02759 ^b^ | 0.97 | Deoxyuridine 5'-triphosphate nucleotidohydrolase, putative | 1e-90 |
| TTHERM_00684560 | 0.97 | Protein kinase domain containing protein | 1e-31 |
| TTHERM_01041990 | 0.97 | Predicted Tetrahymena ORF ^a^ | - |
| TTHERM_00083360 | 0.97 | *TAP45* telomerase associated protein | 0 |
| TTHERM_00670160 | 0.97 | Predicted Tetrahymena ORF ^a^ | - |
| TTHERM_00313280 | 0.97 | SNF2 family N-terminal domain containing protein | 0 |
| TTHERM_00371110 | 0.97 | Ran-binding protein 7, putative [Brugia malayi] | 0.11 |
| TTHERM_00147540 | 0.97 | mCG132433, isoform CRA_a [Mus musculus] | 16 |
| TTHERM_00857820 | 0.97 | Predicted Tetrahymena ORF ^a^ | - |
| 3720.m00655 ^b^ | 0.97 | Ku P70 DNA helicase | 0 |
| TTHERM_00825470 | 0.97 | Protein kinase domain containing protein | 0 |
| 3712.m00915 ^c^ | 0.96 | Diacylglycerol kinase catalytic domain | 2e-69 |
| TTHERM_00571880 | 0.96 | DNA primase, eukaryotic-type, small subunit, putative family protein | 0 |
| TTHERM_00378760 | 0.96 | Predicted Tetrahymena ORF ^a^ | - |
| TTHERM_00975340 | 0.96 | Predicted Tetrahymena ORF ^a^ | - |
| TTHERM_00840020 | 0.96 | Similar to hypothetical protein (predicted), isoform CRA_a [Rattus  norvegicus] | 1e-52 |
| TTHERM_01049190 | 0.96 | Predicted Tetrahymena ORF ^a^ | - |
| TTHERM_00188610 | 0.96 | Predicted Tetrahymena ORF ^a^ | - |
| TTHERM_00825530 | 0.96 | Origin recognition complex subunit 5 [Mus musculus] | 3e-11 |
| TTHERM_00974310 | 0.96 | Predicted Tetrahymena ORF ^a^ | - |
| TTHERM_00780750 | 0.96 | ATPase, AAA family protein | 0 |
| TTHERM_00581740 | 0.96 | Predicted Tetrahymena ORF ^a^ | - |
| 3696.m00954 ^b^ | 0.96 | Putative signal transduction protein containing EAL and modified  HD-GYP domains [Marinomonas sp. MED121] | 3.1 |
| TTHERM_00939110 | 0.96 | BRCA1 C Terminus (BRCT) domain | 0 |
| TTHERM_00431250 | 0.96 | mRNA capping enzyme, large subunit family protein | 0 |
| TTHERM_00684720 | 0.96 | Exonuclease family protein | 0 |
| THERM_00578960 | 0.96 | HE65 [Antheraea pernyi nucleopolyhedrovirus] |  |
| TTHERM_00765120 | 0.96 | Predicted Tetrahymena ORF ^a^ | - |
| TTHERM_00703910 | 0.96 | MCM2/3/5 family protein | 0 |
| TTHERM_00530720 | 0.96 | Nucleoporin autopeptidase | 0 |
| TTHERM_00492860 | 0.96 | Predicted Tetrahymena ORF ^a^ | - |
| TTHERM_00558280 | 0.96 | Predicted Tetrahymena ORF ^a^ | - |
| TTHERM_00773400 | 0.96 | A-T [Homo sapiens] | 3.2 |
| TTHERM_00556700 | 0.96 | Predicted Tetrahymena ORF ^a^ | - |
| TTHERM_00624870 | 0.95 | ERCC4 domain containing protein | 0 |
| TTHERM_00444430 | 0.95 | Predicted Tetrahymena ORF ^a^ | - |
| TTHERM_00158030 | 0.95 | Predicted Tetrahymena ORF ^a^ | - |
| TTHERM_00502480 | 0.95 | EF hand family protein | 2e-90 |
| TTHERM_01080600 | 0.95 | Nucleoporin autopeptidase | 1e-11 |
| TTHERM_00448570 | 0.95 | MCM2/3/5 family protein | 0 |
| TTHERM_01004990 | 0.95 | Predicted Tetrahymena ORF ^a^ | - |
| TTHERM_00266400 | 0.95 | Thioesterase family protein | 1e-55 |
| TTHERM_00101160 | 0.95 | Nuclear pore protein [Entamoeba histolytica HM-1:IMSS] | 1e-34 |
| TTHERM_00637610 | 0.95 | Similar to 1200003M09Rik protein (predicted), isoform CRA_b [Rattus  norvegicus] | 5e-04 |
| TTHERM_01087940 | 0.95 | Methyltransferase, UbiE/COQ5 family protein | 1e-21 |
| TTHERM_00933140 | 0.95 | Predicted Tetrahymena ORF ^a^ | - |
| TTHERM_00558470 | 0.95 | TPR Domain containing protein | 0.042 |
| TTHERM_00726470 | 0.94 | WGR domain containing protein | 0 |
| TTHERM_01230100 | 0.94 | 2-octaprenyl-6-methoxyphenyl hydroxylase [Fulvimarina pelagi HTCC2506] | 3.5 |
| TTHERM_00572090 | 0.94 | F-box domain containing protein | 0 |
| TTHERM_00540340 | 0.94 | Predicted Tetrahymena ORF ^a^ | - |
| TTHERM_00439320 | 0.94 | Ribosomal protein L5 [Stigeoclonium helveticum] |  |
| TTHERM_00424700 | 0.94 | DNA polymerase catalytic subunit | 0 |
| TTHERM_00529980 | 0.94 | Josephin family protein | 0 |
| TTHERM_00554270 | 0.94 | MCM2/3/5 family protein | 0 |
| TTHERM_00004840 | 0.94 | Predicted Tetrahymena ORF ^a^ | - |
| TTHERM_00497240 | 0.94 | Predicted Tetrahymena ORF ^a^ | - |
| 13.m04920 ^b^ | 0.94 | ABC transporter ATP-binding protein [Bacteroides fragilis YCH46] | 7.7 |
| TTHERM_00974300 | 0.94 | Replication factor C complex, putative [Brugia malayi] | 2e-05 |
| 117.m01266 ^c^ | 0.94 | F-box protein At4g09920 | 2.1 |
| TTHERM_00338300 | 0.93 | OSJNBa0022H21.19 [Oryza sativa (japonica cultivar-group)] | 5e-09 |
| TTHERM_00865150 | 0.93 | WGR domain containing protein | 0 |
| TTHERM_00335700 | 0.93 | importin alpha [Pagrus major] | 2e-11 |
| TTHERM_00647510 | 0.93 | Predicted Tetrahymena ORF ^a^ | - |
| TTHERM_00483530 | 0.93 | Protein kinase domain containing protein. Sequence similarity to the PEK protein kinase family | 0 |
| TTHERM_00382290 | 0.93 | Mnd1 family protein | 0 |
| TTHERM_00522820 | 0.93 | Predicted Tetrahymena ORF ^a^ | - |
| TTHERM_00277550 | 0.93 | MCM2/3/5 family protein | 0 |
| TTHERM_00394660 | 0.93 | Predicted Tetrahymena ORF ^a^ | - |
| TTHERM_00842380 | 0.93 | Myb-like DNA-binding domain containing protein | 0 |
| TTHERM_00277530 | 0.93 | CDT1 protein [Homo sapiens] | 4e-13 |
| TTHERM_00193420 | 0.93 | PHD finger family protein [Arabidopsis thaliana] | 1e-10 |
| TTHERM_00011650 | 0.93 | Mating-type switching protein swi10, putative | 0 |
| TTHERM_00245200 | 0.93 | RNA binding motif protein 35A isoform 2 [Homo sapiens] | 7e-34 |
| 3697.m01708 ^b^ | 0.92 | ADR143Wp [Ashbya gossypii ATCC 10895] | 0.35 |
| TTHERM_00621200 | 0.92 | Armadillo/beta-catenin-like repeat family protein | 0 |
| 16.m05342 ^b^ | 0.92 | Predicted Tetrahymena ORF ^a^ | - |
| TTHERM_00681750 | 0.92 | Poly polymerase and DNA-Ligase Zn-finger region family protein | 0 |
| TTHERM_00371130 | 0.92 | Predicted Tetrahymena ORF ^a^ | - |
| TTHERM_00492970 | 0.92 | ImpA [Hemiselmis andersenii] | 4e-08 |
| TTHERM_00670140 | 0.92 | Predicted Tetrahymena ORF ^a^ | - |
| TTHERM_00441860 | 0.92 | Predicted Tetrahymena ORF ^a^ | - |
| 91.m01832 ^b^ | 0.92 | Predicted Tetrahymena ORF ^a^ | - |
| TTHERM_00684490 | 0.92 | DNA repair helicase (rad3) | 0 |
| TTHERM_00962200 | 0.92 | Importin-beta N-terminal domain containing protein | 0 |
| TTHERM_00276060 | 0.92 | Chromosome segregation 1-like [Danio rerio] | 1e-44 |
| TTHERM_00079520 | 0.92 | *GTU1* | 0 |
| TTHERM_00046760 | 0.91 | GA15208-PA [Drosophila pseudoobscura] | 2e-36 |
| TTHERM_00082190 | 0.91 | BTB/POZ domain containing protein [Trichomonas vaginalis G3] | 2.6 |
| TTHERM_00091620 | 0.91 | Nucleoporin Nup100/Nsp100 [Plasmodium yoelii yoelii str. 17XNL] | 3e-09 |
| TTHERM_00576810 | 0.91 | Predicted Tetrahymena ORF ^a^ | - |
| TTHERM_00442300 | 0.91 | Anti-silencing protein, ASF1-like containing protein | 3e-145 |
| TTHERM_01014700 | 0.91 | Histone deacetylase family protein | 0 |
| TTHERM_00865050 | 0.91 | Origin recognition complex, subunit 1 [Gallus gallus] | 1e-57 |
| TTHERM_00821890 | 0.91 | Predicted Tetrahymena ORF ^a^ | - |
| TTHERM_00467830 | 0.91 | Phage head-tail adaptor, putative family protein | 1e-61 |
| TTHERM_01107420 | 0.91 | Proliferating cell nuclear antigen (pcna) | 6e-147 |
| 3.m10039 ^b^ | 0.91 | Predicted Tetrahymena ORF ^a^ | 0 |
| TTHERM_01044400 | 0.91 | Integral membrane protein DUF6 containing protein | 0 |
| TTHERM_00387050 | 0.91 | DNA ligase IV | 0 |
| TTHERM_00475350 | 0.901 | Kinetochore protein Hec1 (Kinetochore-associated protein 2) | 9e-07 |
| TTHERM_00636920 | 0.901 | DNA polymerase family B containing protein | 0 |
| TTHERM_00001140 | 0.901 | Transporter, EamA family [Bacillus cereus ATCC 10987] | 2.2 |

Footnotes *, a and b as in Table S3.
