# Supplementary material for: Microarray Analyses of Gene Expression during the Tetrahymena thermophila Life Cycle
Source: PLoS One. 2009 Feb 10;4(2):e4429. doi: 10.1371/journal.pone.0004429 (PMC2636879; doi:10.1371/journal.pone.0004429)
Supplement: Table S11 — The accession numbers of microarray data in this paper as submitted to the NCBI Gene Expression Omnibus. (0.05 MB DOC) [file pone.0004429.s012.doc]

**Table S11. The accession numbers of microarray data in this paper as submitted to the NCBI Gene Expression Omnibus.**

| **Accession Title**  **Number** |
| --- |
| GSE11300 Series: Genome-Wide Microarray Analysis Reveals Massive  Changes in Gene Expression During Conjugation in *Tetrahymena*  *thermophila* |
| GPL6759 Platform: *Tetrahymena thermophila* genome-wide oligonucleotide  microarray |
| GSM283687 Tetrahymena_L1-l |
| GSM283690 Tetrahymena_L1-m |
| GSM283691 Tetrahymena_L1-h |
| GSM284355 Tetrahymena_L2-l |
| GSM284357 Tetrahymena_L2-m |
| GSM284360 Tetrahymena_L2-h |
| GSM284362 Tetrahymena_L3-l |
| GSM284363 Tetrahymena_L3-m |
| GSM284364 Tetrahymena_L3-h |
| GSM285363 Tetrahymena_S1-0 |
| GSM285542 Tetrahymena_S1-3 |
| GSM285543 Tetrahymena_S1-6 |
| GSM285544 Tetrahymena_S1-9 |
| GSM285545 Tetrahymena_S1-12 |
| GSM285546 Tetrahymena_S1-15 |
| GSM285547 Tetrahymena_S1-24 |
| GSM285554 Tetrahymena_S2-0 |
| GSM285555 Tetrahymena_S2-3 |
| GSM285556 Tetrahymena_S2-6 |
| GSM285557 Tetrahymena_S2-9 |
| GSM285558 Tetrahymena_S2-12 |
| GSM285559 Tetrahymena_S2-15 |
| GSM285560 Tetrahymena_S2-24 |
| GSM285561 Tetrahymena_S3-0 |
| GSM285562 Tetrahymena_S3-3 |
| GSM285563 Tetrahymena_S3-6 |
| GSM285564 Tetrahymena_S3-9 |
| GSM285565 Tetrahymena_S3-12 |
| GSM285566 Tetrahymena_S3-15 |
| GSM285567 Tetrahymena_S3-24 |
| GSM285570 Tetrahymena_C1-0 |
| GSM285572 Tetrahymena_C1-2 |
| GSM285574 Tetrahymena_C1-4 |
| GSM285575 Tetrahymena_C1-6 |
| GSM285576 Tetrahymena_C1-8 |
| GSM285578 Tetrahymena_C1-10 |
| GSM285579 Tetrahymena_C1-12 |
| GSM285580 Tetrahymena_C1-14 |
| GSM285582 Tetrahymena_C1-16 |
| GSM285583 Tetrahymena_C1-18 |
| GSM285586 Tetrahymena_C2-0 |
| GSM285587 Tetrahymena_C2-2 |
| GSM285588 Tetrahymena_C2-4 |
| GSM285589 Tetrahymena_C2-6 |
| GSM285590 Tetrahymena_C2-8 |
| GSM285591 Tetrahymena_C2-10 |
| GSM285592 Tetrahymena_C2-12 |
| GSM285593 Tetrahymena_C2-14 |
| GSM285595 Tetrahymena_C2-16 |
| GSM285596 Tetrahymena_C2-18 |
